# Dysregulation of Iron Metabolism-Linked Genes at Myocardial Tissue and Cell Levels in Dilated Cardiomyopathy

**DOI:** 10.3390/ijms24032887

**Published:** 2023-02-02

**Authors:** Ilaria Massaiu, Jeness Campodonico, Massimo Mapelli, Elisabetta Salvioni, Vincenza Valerio, Donato Moschetta, Veronika A. Myasoedova, Maria Domenica Cappellini, Giulio Pompilio, Paolo Poggio, Piergiuseppe Agostoni

**Affiliations:** 1 Centro Cardiologico Monzino, IRCCS, 20138 Milan, Italy; 2 Dipartimento di Scienze Farmacologiche e Biomolecolari, Università degli Studi di Milano, 20122 Milan, Italy; 3 UOC General Medicine, Fondazione IRCCS Ca’ Granda Ospedale Maggiore Policlinico, 20122 Milan, Italy; 4 Department of Clinical Sciences and Community Health, University of Milan, 20122 Milan, Italy; 5 Department of Biomedical, Surgical and Dental Sciences, University of Milan, 20122 Milan, Italy

**Keywords:** iron metabolism dysregulation, DCM, heart failure, bulk RNA-seq, snRNA-seq, DEG analysis

## Abstract

In heart failure, the biological and clinical connection between abnormal iron homeostasis, myocardial function, and prognosis is known; however, the expression profiles of iron-linked genes both at myocardial tissue and single-cell level are not well defined. Through publicly available bulk and single-nucleus RNA sequencing (RNA-seq) datasets of left ventricle samples from adult non-failed (NF) and dilated cardiomyopathy (DCM) subjects, we aim to evaluate the altered iron metabolism in a diseased condition, at the whole cardiac tissue and single-cell level. From the bulk RNA-seq data, we found 223 iron-linked genes expressed at the myocardial tissue level and 44 differentially expressed between DCM and NF subjects. At the single-cell level, at least 18 iron-linked expressed genes were significantly regulated in DCM when compared to NF subjects. Specifically, the iron metabolism in DCM cardiomyocytes is altered at several levels, including: (1) imbalance of Fe^3+^ internalization (*SCARA5* down-regulation) and reduction of internal conversion from Fe^3+^ to Fe^2+^ (*STEAP3* down-regulation), (2) increase of iron consumption to produce hemoglobin (*HBA1/2* up-regulation), (3) higher heme synthesis and externalization (*ALAS2* and *ABCG2* up-regulation), (4) lower cleavage of heme to Fe^2+^, biliverdin and carbon monoxide (*HMOX2* down-regulation), and (5) positive regulation of hepcidin (*BMP6* up-regulation).

## 1. Introduction

Heart failure (HF) is defined as one of the main clinical and public health burdens with multifactorial symptoms and high worldwide prevalence. Dilated cardiomyopathy (DCM) is one of the leading causes of HF characterized by left ventricle dilation and systolic dysfunction in the absence of known abnormal loading conditions or significant coronary artery disease [1,2]. Heart transplantation (HTx) and left ventricular assist device (LVAD) implantation remain the most common surgical options to treat DCM patients, despite the limitations to treating the end-stage of disease and the numerous criticisms on life impact and long-term outcomes [3,4]. Therefore, new therapeutic strategies are continuously explored.

DCM is regarded as a multiple disease entity linked to a number of genetic, nutritional, inflammatory, infective, metabolic, and environmental causes [5]. Thus, due to the heterogeneity and complexity of this multi-faced disease, a deeper understanding of the underlying biological mechanisms would pave the way for new precision medicine strategies. In fact, with advances in next-generation sequencing (NGS) technologies and big data analysis, it will likely be possible in the future to individualize specific gene patterns and thus guide therapy and improve prognosis.

Among the metabolic processes associated with HF, iron metabolism disorders, ranging from iron deficiency (ID) to iron overload, are gaining attention [6,7,8,9]. The understanding of iron mechanisms involved in HF at the systemic level is being addressed; however, at the cardiac level, they remain elusive.

Iron is an essential micronutrient for a wide range of physiological processes, including oxygen transport and storage, cardiac and skeletal muscle metabolism, mitochondrial respiration, protein synthesis, and degradation [10,11,12,13]. At the same time, iron has the potential to be toxic if present in excess because it catalyzes the generation of reactive radicals that can damage DNA, protein, and lipids [14]. Consequently, systemic and cellular iron homeostasis need to be tightly controlled. More than 70% of body iron is present as heme within hemoglobin (or myoglobin) of developing erythroblasts and mature erythrocytes; it is used to bind oxygen and transport it to tissues [13,15]. Circulating iron, mainly associated with transferrin (TF), is delivered to erythrocytes and other cells in the body via specific uptake mechanisms [13]. TF-bound iron binds its receptor, transferrin receptor protein 1 (TfR1), leading the iron internalization by endocytosis. While the non-TF-bound form is taken up through divalent metal transporter 1 (DMT1), which is ubiquitously present on the surface of cells. In addition, the non-TF-bound iron can be transported by voltage-gated calcium channels of cardiomyocytes under iron overload conditions [16]. In the endosome, ferric iron (Fe^3+^) is reduced to ferrous iron (Fe^2+^) by the STEAP (six-transmembrane epithelial antigen of the prostate) family of metalloreductases and then released into the cytoplasm by DMT1 [10,13]. Once in the cytoplasm, Fe^2+^ is utilized for heme synthesis by erythroid 5-aminolevulinic acid synthase (ALAS2), stored in iron-ferritin complexes, taken up by mitochondria for the synthesis of heme and iron-sulfur (Fe-S) clusters, or exported out of the cell via ferroportin [9]. The mechanisms regulating systemic iron homeostasis involve predominantly hepcidin and ferroportin, which work together to regulate the flow of iron from cells into the systemic circulation. Hepcidin, a small peptide hormone synthesized mainly by hepatocytes, interacts with ferroportin on target cells in a negative feedback fashion. In particular, it blocks intestinal absorption of iron and removes iron from the circulation, trapping it in enterocytes, hepatocytes, and macrophages [8,17]. The heart has the second-highest expression levels of hepcidin, which acts as an autocrine protein to regulate iron levels in cardiomyocytes [9,18]. Moreover, a post-translation regulation of cellular iron metabolism is achieved via iron response elements (IREs) and iron regulatory proteins (IRPs). Specifically, under conditions of iron deficiency, IRE/IRPs interactions promote the uptake of iron by stabilizing transferrin receptor (*TFRC*) and *DMT1* mRNAs against the degradation and preventing its sequestration and efflux, inhibiting the translation of ferritin, ferroportin, and ALAS2 [19]. 

Systemic ID is one of the most frequent comorbidities in patients with HF, with an observed prevalence reaching 58% [20]. Systemic ID carries a relevant prognostic value on top of the usual cardiac risk factors, irrespective of the presence of anemia [21,22]. In the last decade, different clinical trials demonstrated the benefit of iron supplementation with ferric carboxymaltose in patients with ID and HF [23], highlighting the central role of iron metabolism in multiple systemic mechanisms responsible for HF progression [22,24]. In particular, it has been shown that intravenous iron supplementation improves the quality of life and exercise capacity, and reduces re-hospitalization in patients with HF and ID [23,25]. On the other hand, myocardial ID (MID) in HF, which is clinically associated with greater adverse remodeling including interstitial fibrosis and cardiac hypertrophy, remains largely unexplored [26]. MID is poorly related to systemic iron homeostasis biomarkers, so that whether MID may be part of the cause of systemic ID or a consequence is unknown [27]. Although with lower prevalence with respect to systemic ID, iron overload can lead to cardiomyopathy and, ultimately, HF via oxidative damage [28]. Recently, the link between iron overload and cardiovascular disease was attributed to ferroptosis [29,30]. Ferroptosis is an iron-dependent form of programmed cell death involving lipid hydroperoxide accumulation [31]. Both in-vitro and in-vivo evidence supports the pathophysiological role of ferroptosis on many forms of cardiovascular dysfunctions [32]. Indeed, many aspects of iron metabolism, such as its absorption, storage, and utilization, have important roles in regulating ferroptosis. 

Albeit iron molecular mechanisms have been deeply investigated in different physiological and pathological processes, only a few genes have been studied at the myocardial cell level, and a comprehensive evaluation of DCM is lacking [30]. Accordingly, through a quantitative analysis of public myocardial gene expression data at tissue (bulk RNA sequencing) and cell (single-nucleus RNA sequencing) levels, we aim to evaluate the possible dysregulation of iron metabolism-linked genes in DCM. Therefore, we proceeded in a step-wise fashion evaluating at first the reliability of a large bulk RNA-seq dataset, created from the collection of public data, by confirming the main gene players involved in DCM pathophysiology. Thus, we analyzed the iron metabolism-related gene abnormalities in DCM both at myocardial tissue and at single-cell levels.

## 2. Results

### 2.1. Bulk RNA-Seq Analysis in DCM

**Figure 1:**
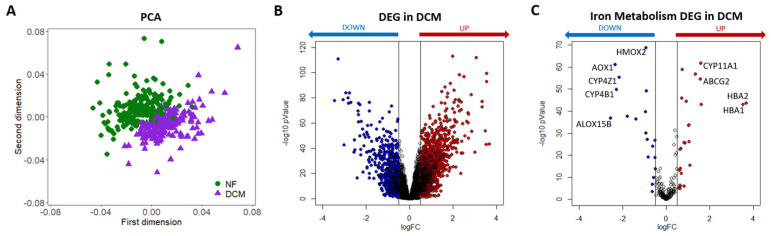
Differentially expressed genes in heart failure (HF). (**A**) Principal component analysis (PCA) of RNA-seq data. Each dot represents one sample. Green circles: non-failing subjects (NF). Purple triangles: dilated cardiomyopathy (DCM) patients. (**B**) Volcano plot showing the results of the differential expression analysis of DCM with respect to NF, on the entire set of genes. (**C**) Volcano plot showing just the iron-linked genes were significantly regulated in DCM. The genes with |log(FC)| ≥ 0.5 and adjusted *p* Value < 0.05 are regarded as the differentially expressed genes (DEGs) in DCM in comparison to the NF group. In particular, red and blue dots denote significantly up-regulated (adjusted *p* Value < 0.05 and log(FC) ≥ 0.5) and down-regulated (adjusted *p* Value < 0.05 and log(FC) ≤ −0.5) genes in DCM, respectively. The empty dots represent the unchanged genes.

Considering the gene expression analysis of DCM vs. non-failed (NF) groups, each composed of 218 samples, two well-defined clusters were highlighted, representing their distances through principal component analysis (PCA, Figure 1A). In particular, we identified 2397 DEGs, of which 1768 and 629 genes were up- and down-regulated, respectively (Figure 1B and Supplementary Table S1). Among the top significantly regulated genes, we found numerous well-established hallmarks of heart dysfunction [31], confirming the validity of the comparison. Indeed, the most DCM-linked genes, revealed by our statistical analysis, were hemoglobin subunit alpha 1 (*HBA1*, logFC = 3.52), hemoglobin subunit alpha 2 (*HBA2*, logFC = 3.68), hemoglobin subunit beta (*HBB*, logFC = 3.49), secreted frizzled-related protein (*SFRP4*, logFC = 3.54), natriuretic peptide A (*NPPA*, logFC = 3.00) and B (*NPPB*, logFC = 2.37), as well, interleukin-1 receptor-like protein 1 (*IL1RL1*, logFC = −3.44), alpha–1 antichymotrypsin (*SERPINA3*, logFC = −3.28), myosin heavy chain 6 (*MYH6*, logFC = −2.92), tubulin alpha 3E and 3D (*TUBA3E*, logFC = −2.82 and *TUBA3D*, logFC = −2.80), all with adj *p* Value < 0.001.

### 2.2. Implication of Iron Metabolism at Cardiac Tissue Level in DCM (Bulk RNA-Seq Data)

From the bulk RNA-seq analysis of DCM vs. NF samples, we identified 223 expressed genes among the 272 iron metabolism-linked genes collected from MSigDB [32]. Specifically, 44 genes were differentially regulated, being 25 up- and 19 down-regulated (Figure 1C).

Among the top 15 up- and down-regulated iron-linked genes in DCM (Table 1), some important protagonists of iron regulation were identified, such as *HBA1* and *HBA2* (the two highest up-regulated genes), heme oxygenase 1 and 2 (*HMOX1*, logFC = −0.64 and *HMOX2*, logFC = −0.93), ATP binding cassette Subfamily G member 2 (*ABCG2*, logFC = 1.56), six-transmembrane epithelial antigen of prostate 3 and 4 (*STEAP3*, logFC = −0.91 and *STEAP4*, logFC = −0.88), and *TFRC* (logFC= −0.83), all with adj *p* Value < 0.05.

### 2.3. Implication of Iron Metabolism at Cardiac Cell Level in DCM (snRNA-Seq Data)

**Figure 2:**
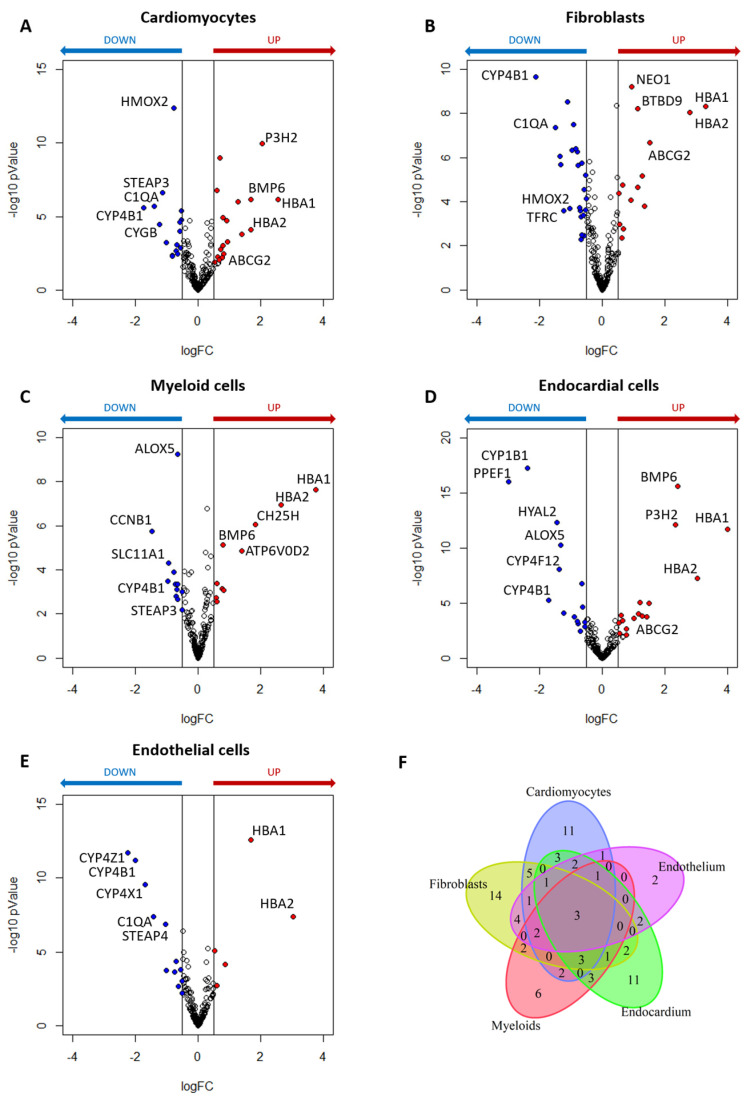
Differentially expressed iron-linked genes analysis for specific cell types in dilated cardiomyopathy (DCM) patients in comparison to the donors (NF). (**A**) Volcano plot for visualization of differential expressed genes (DEGs) in cardiomyocytes. (**B**) Volcano plot for visualization of DEGs in fibroblasts. (**C**) Volcano plot for visualization of DEGs in myeloid cells. (**D**) Volcano plot for visualization of DEGs in endocardial cells. (**E**) Volcano plot for visualization of DEGs in endothelial cells. (**F**) Venn diagram exhibited the overlapping of the DEGs among the five cell types. Red and blue dots denote significantly up-regulated (adjusted *p* Value < 0.05 and log(FC) ≥ 0.5) and down-regulated (adjusted *p* Value < 0.05 and log(FC) ≤ −0.5) genes in DCM, respectively. The empty dots represent the unchanged genes.

Using the public single-nucleus RNA sequencing (snRNA-seq) data [33], the relevance of iron-linked genes was explored across the 14 major cardiac cell types identified from 26 NF and 12 DCM patients. Among the 272 iron-linked genes, 95 of them were expressed at least in one of the NF or DCM cell populations with a percentage of expression ≥ 20% (Supplementary Figure S1). Then, we focused the differential expression analysis on the five cell types showing the greatest differences in gene expression, namely cardiomyocytes, fibroblasts, myeloid, endocardial, and endothelial cells (Supplementary Table S2). Specifically, 34 iron-linked DEGs were found in cardiomyocytes, 18 of which up- and 16 down-regulated (Figure 2A), 38 were identified in fibroblasts, 14 up- and 24 down-regulated (Figure 2B), 22 DEGs were in myeloid cells, ten up- and 12 down-regulated (Figure 2C), 31 in endocardial cells, 16 up- and 15 down-regulated (Figure 2D), and 18 in endothelial cells, and 6 up- and 12 down-regulated (Figure 2E). Of these differentially regulated iron-linked genes emerging across the five populations, a significant amount was shared (Figure 2F). Of note, *HBA1*, *HBA2*, and Cytochrome P450 Family 4 Subfamily B Member 1 (*CYP4B1*) genes were significantly regulated in all the considered cell populations; the first two up-regulated while the last one down-regulated.

## 3. Discussion

In the present study, we showed that several genes controlling iron-related mechanisms are altered in DCM both at myocardial tissue and cell level, leading, as a final result, to reduced availability of iron in the left ventricle. At the cardiac tissue level, 44 iron-linked genes were found differentially regulated, while at the single-cell level, considering the five cell populations with a high iron-linked gene expression pattern, several differentially expressed iron-linked genes were identified and, of those, 3 were common to all cell types.

In the last few years, the benefit of iron supplementation in HF patients was largely documented in a number of randomized trials, but the underlying intrinsic pathophysiological mechanisms are yet to be defined. Of note, successful iron supplementation was documented mostly with i.v. ferric carboxymaltose at high doses [8].

We dedicated our work specifically to DCM and not HF in general because more data are available in DCM patients and to reduce the number of confounder elements likely elicited by other forms of HF such as those related to myocardial ischemia/infarction. Moreover, it is acknowledged that NF is a mixture of healthy and non-cardiac subjects representing a reliable control group for DCM patients.

The comprehensive evaluation of gene expression data represents a successful tool to increase knowledge on the metabolic processes involved in DCM. We performed a two steps DEG analysis on bulk RNA-seq data collected from the entire cardiac tissue of NF and DCM patients. First, the reliability of our results was confirmed by evaluating all the different expressed genes in DCM and comparing them to previously reported data [12]. Indeed, numerous genes previously reported as important markers in HF, such as *SERPINA3*, aquaporin 4 (*AQP4)*, *MYH6*, *NPPA*, and *NPPB*, resulted as highly regulated in our analysis. Moreover, at the cardiac tissue level, extracellular matrix remodeling and inflammation were regarded as the principal processes underlying cardiac dysfunctions [34]. The second step was dedicated to iron gene metabolism regulation at the whole cardiac tissue level in DCM. From overall myocardial tissue analysis, we found that several iron-linked genes were up- or down-regulated, including some key players in iron metabolism regulation. Of note, the specific function of some iron-linked genes found as up- or down-regulated is still unknown.

To better understand iron gene abnormalities in the myocardium of DCM patients, we deepened the analysis from tissue to the single-cell level, observing consistent results. As already known, the myocardium is composed of multiple cell types. From the 14 cell populations identified in the myocardium tissue, we focused our attention on cardiomyocytes and on cell types that showed the greatest differences in gene expression, namely fibroblasts, myeloid, endocardial, and endothelial cells. Specifically, the iron-metabolism- linked genes whose abnormalities have a recognized role in iron metabolism were investigated. We showed an evident dysregulation of iron metabolism for these specific cell types in DCM ventricles (Table 2).

**Figure 3:**
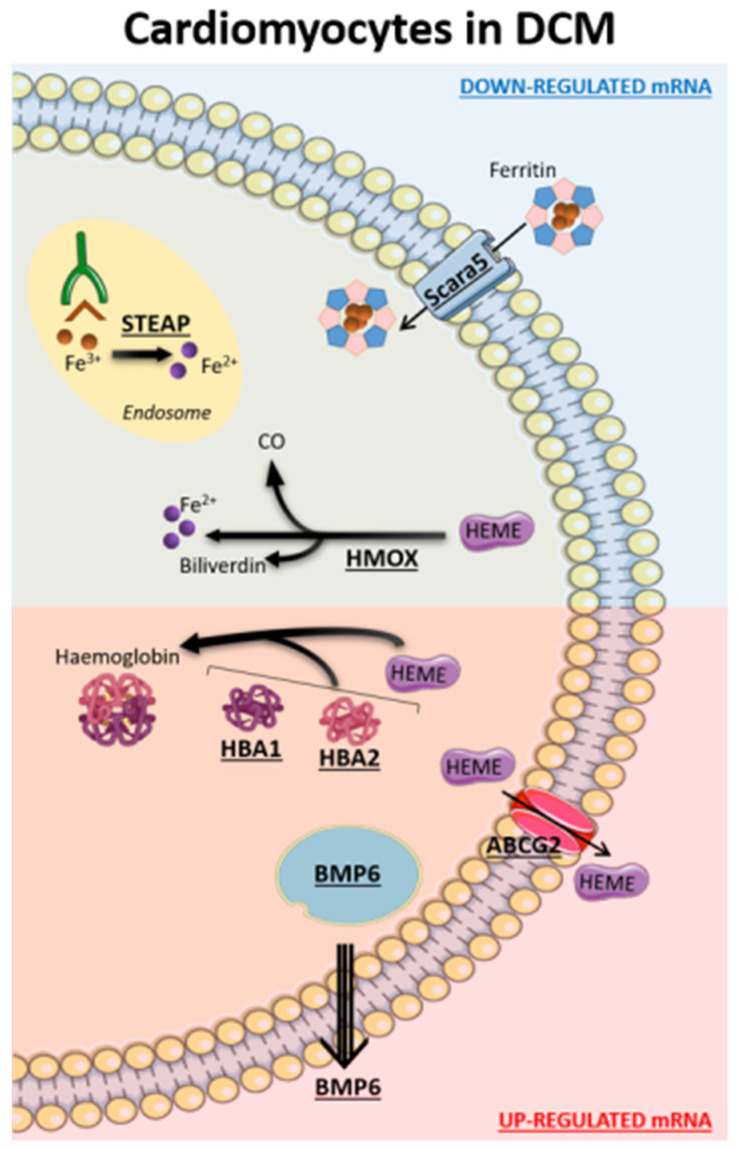
Iron-linked affected genes in dilated cardiomyopathy (DCM) cardiomyocytes. Down-regulated proteins are represented in the region with the blue background, while the up-regulated ones are in the region with the red background. ATP binding cassette subfamily G member 2 (ABCG2), delta-aminolevulinic synthase 2 (ALAS2), bone morphogenetic protein 6 (BMP6), hemoglobin subunit alpha 1/2 (HBA1/2), heme oxygenase 1/2 (HMOX1/2), scavenger receptor class A member 5 (SCARA5), solute carrier family 25 member 28 (SLC25A28), six-transmembrane epithelial antigen of the prostate 3/4 (STEAP3/4).

The main iron abnormalities in DCM cardiomyocytes, depicted in Figure 3, lead to a significant reduction of intracellular Fe^2+^ availability and heme accumulation. One of the major regulatory sites for iron homeostasis is represented by *SCARA5* because it binds and internalizes ferritin, which allows Fe^3+^ entrance and its transport to the endosome. Here, Fe^3+^ is released and reduced to Fe^2+^ by *STEAP3* [35]. Of note, decreased *SCARA5* expression in cardiomyocytes, leading to iron deficiency, was previously shown in failing human hearts [36]. Similarly, *STEAP3* gene, essential in iron homeostasis, apoptosis, and inflammation, was reported as a key negative regulator of cardiac hypertrophy [37]. In our analyses, both the ferritin receptor *SCARA5* and the ferrireductase *STEAP3* were down-regulated, corroborating the iron metabolism dysregulation in the DCM context.

A complex setting arose for heme homeostasis in DCM cardiomyocytes. Heme is an essential co-factor involved in multiple biological processes, including the transport and storage of oxygen, but it is considered toxic at high concentrations because it catalyzes oxidation and breakdown of protein and DNA, and damages membrane-bound organelles [38]. DCM cardiomyocytes are characterized by an increased expression of *HBA1/2* and *HBB* genes, encoding alpha and beta globin chains, respectively, which are strongly associated with an increase in heme utilization. Indeed, one heme group is associated with each chain via its ferrous ion and a molecule of hemoglobin is formed. Interestingly, high expression levels of these genes, induced by oxidative stress, were previously correlated with HF development and arrhythmia genesis [39]. Recently, Khechaduri et al. [40] observed, in addition to a significant increase in heme levels in HF, a high expression of *ALAS2*, a rate-limiting enzyme in heme synthesis. They also demonstrated that overexpression of *ALAS2* and the resulting accumulation of heme levels were associated with excess production of reactive oxygen species and cell death in HF. Accordingly, we identified a significant up-regulation of *SLC25A28* and *ALAS2* genes in DCM cardiomyocytes, which together are involved in Fe^2+^ mitochondrial internalization and heme synthesis. In addition, we observed a lower utilization of heme, by *HMOX2*, for the synthesis of Fe^2+^, carbon monoxide (CO), and biliverdin, which are anti-inflammatory and antioxidant molecules. Of note, numerous studies showed the impact of the constitutive *HMOX2* expression, together with *HMOX1*, in cardiovascular disease [30]. In particular, a *HMOX2* deficiency was reported to be associated with a high expression of gp91phox (*NOX2*), interleukin 1 beta (*IL1B*), and interleukin 6 (*IL6*) in endothelial cells (EC), responsible for oxidative stress induction as well as pro-inflammatory phenotype [41]. Moreover, limited *HMOX2* activity promotes apoptosis [42] and causes the delayed wound healing response [43]. All these mechanisms highlight the dangerous effects of high intracellular levels of heme. However, cells are able to activate compensatory mechanisms to reduce toxic levels induced by highly expressed proteins. In this regard, we found that *ABCG2* up-regulation, actively exporting heme, could be one of these compensatory processes decreasing heme levels in DCM cardiomyocytes, thus trying to rebalance the iron metabolism homeostasis.

Finally, we observed an increased expression of bone morphogenetic protein 6 gene (*BMP6)* which is a key endogenous regulator of hepcidin expression [44]. Of note, hepcidin serum levels mostly correlate with iron serum levels, and it has been shown that hepcidin plays a pivotal role in the systemic iron metabolism. However, the role of systemic hepcidin in DCM, at cardiac tissue or cell levels, is at present unknown [45].

Similarly, the other cell populations in DCM patients are characterized by numerous gene-linked iron abnormalities (Supplementary Figure S2 and Table 2). All analyzed cells except endocardial cells show down-regulation of *SCARA5* and, therefore, likely a reduced ferritin intracellular availability, and all but fibroblast show a down-regulation of *STEAP3* and therefore a lower reduction of Fe^3+^ to Fe^2+^. Fibroblast but not myeloid, endocardial and endothelial cells show down-regulation of *HMOX* gene. Moreover, all these four cells have an increased heme production shown by *HBA* up-regulation as well as up-regulation of heme transporter at membrane cell level in all cells but fibroblast. Similarly, *BMP6* was up-regulated in all cells but fibroblast. Consequently, a reduced Fe^2+^ availability is not limited to the cardiomyocytes, but it is shared by several cardiac cells.

Of note, out of 20 genes linked to ferroptosis, the type of cell death induced by iron accumulation, we found only 6 of them linked to DCM [29]. Ferritin (*FTH1*), which plays a central role in iron metabolism by storing excess cellular iron and, therefore, in preventing iron-induced ferroptosis [46], was down-regulated in DCM. Similarly, a lower expression of iron chaperone poly(rC)-binding protein 1 (*PCBP1*), binging and delivering Fe^2+^ to ferritin and preventing ferroptosis, characterized cardiomyocytes under DCM condition. Moreover, we found an increased mitochondrial iron uptake in DCM patients (*CISD1/2* were down-regulated and *SLC25A28* up-regulated), which were reported as processes promoting ferroptosis [47,48,49]. On the other hand, the setting of the last two modulators characterizing DCM prevents iron accumulation and subsequent ferroptosis. Indeed, prominin 2 (*PROM2*), an iron exporter, and nuclear receptor coactivator 4 (*NCOA4*), responsible for the degradation of iron-saturated ferritin, were, respectively, up-regulated and down-regulated in DCM. Thus, from our data, we could not confirm that ferroptosis plays a significant role in DCM disease.

A few study limitations should be acknowledged. First, we analyzed only DCM patients and our data cannot be extrapolated to other forms of HF. Second, data were obtained by hearts of patients with severe HF, so that cardiomyocytes’ iron pathways in patients with less advanced HF are unknown. Third, a direct confirmation of our findings in myocardial specimens is needed. Fourth, the present analysis deals with intramyocardial iron-related genes expression, but it is acknowledged that this is only a small part of the all-body iron metabolism and of iron metabolism impairment in HF. Fifth, patients’ demographic data, comorbidities, or other variables (such as iron deficiency or overload presence) that can influence gene expression levels were not available. Finally, our data could not provide any causative information regarding the cardiac iron imbalance found in HF. Indeed, we cannot exclude that iron deficiency in the heart, in some cases, could be primary rather than secondary.

## 4. Materials and Methods

### 4.1. Data Collection

**Figure 4:**
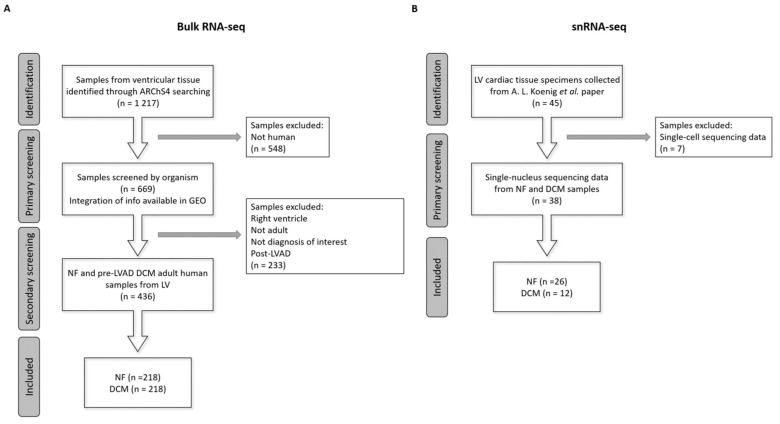
Flow chart of data collection. (**A**) Bulk RNA sequencing (RNA-seq) data of 1217 samples from ventricular tissue were retrieved from ARChS4 database. The primary screening was performed based on the organism, and 669 human samples were selected. Additional information for all samples were manually searched in GEO database and used for the secondary screening. In particular, we finally extracted the data of 436 adult non-failed (NF) and dilated cardiomyopathy (DCM) patients from the left ventricle (LV) before left ventricular assist device (pre-LVAD) implantation. (**B**) Single-cell and single-nucleus RNA sequencing (snRNA-seq and snRNA-seq) data from 45 LV cardiac tissue specimens were extracted from the paper of Koenig et al. [33]. The screening was performed based on the sequencing approach and 38 samples analyzed through snRNA-seq were selected. The latter were composed of 26 NF and DCM patients.

Bulk RNA-seq data of ventricular tissue samples (n = 1217) were collected from ARChS4 database [50]. It is a web resource that provides the majority of published RNA-seq data from human and mouse, starting from the alignment of available FASTQ files from RNA-seq experiments from the Gene Expression Omnibus (GEO). Among them, 548 not human samples were filtered out. Then, additional information regarding each sample was manually searched through the sample accession number (GSM) in the GEO database [51]. In particular, we integrated information on the period of age (prenatal, childhood, adulthood), ventricle (left and right), diagnosis, LVAD implantation, and then performed a secondary screening. Data from LVAD patients were excluded due to the possible interference of LVAD with left ventricle mechanics and chronic LVAD-induced hemolysis, thus affecting iron balance [52]. Hence, 436 specimens from adult NF (n = 218) individuals and DCM (n = 218) patients were employed in the study (Figure 4A and Supplementary Table S3).

Transcriptional signatures of the different cell types detected in left ventricle cardiac healthy and diseased tissues (n = 45) were reported in a recently published work [33]. We extracted the samples processed through snRNA-seq, consisting of 26 NF and 12 DCM patients (Figure 4B). 

A list of genes related to iron metabolism was created based on the molecular signatures database (MSigDB) [13]. In particular, 19 gene sets (Gene Ontology terms (GO): 0005381, 0005506, 0006826, 0006879, 0008198, 0008199, 0010039, 0010040, 0010041, 0033212, 0034755, 0034756, 0034759, 0055072, 0060586, 0071281, 0097577, 0098711, and 1901678) were assembled. Once removing overlapping genes, we obtained an iron metabolism-linked list containing 272 genes (Supplementary Table S4).

### 4.2. Gene Expression Analysis

The raw bulk RNA-seq data were imported into the R software v4.1.1 and the DaMiRseq package v2.6.0 [53] was used to convert the data into log2 counts per million mapped reads (CPM). To avoid expression noise, only genes with more than 5 reads in at least 50% of the total samples were retained. To remove the effects of surrogate variables, such as those related to differences in processing techniques, batches and technical experience of the laboratory where RNA-seq were processed, the matrix of expression values was adjusted by the DaMiR.SVadjust function [53]. 

Thereafter the snRNA-seq data were analyzed using the Seurat v4.1.0 package for R, as indicated by Wang et al. [54]. Finally, for both bulk and snRNA-seq analyses, we focused our attention to the iron metabolism-linked genes. 

### 4.3. Statistical Analysis

Employing the constructed large bulk RNA-seq dataset, we compared DCM patients with NF subjects. The expression data across these two groups were explored by PCA [55], while differential gene expression analysis was carried out to deeply characterize the differences between the groups. This analysis on bulk RNA-seq data was performed on normalized data using the limma package v3.50.3 [56], adjusting for the “Study” effect. The Benjamini–Hochberg procedure was used to control the false discovery rate (FDR).

Differential gene expression analysis using snRNA-seq data was performed using DESeq2 package v1.34.0 for R. A gene expression matrix was extracted from Seurat object for cardiomyocytes, fibroblasts, myeloid, endocardial, and endothelial cells, and normalized using DESeq2 by estimating size factors, in order to obtain an output similar to a traditional bulk sequencing experiment. As performed by Koenig et al. [33], pairwise comparisons were carried out between DCM and NF using the Wald test.

For both analyses, the genes with an absolute value of log2 fold-change (|logFC|) > 0.5 and FDR adjusted *p* Value (adj *p* Value) < 0.05, in DCM group vs. NF, were considered differentially expressed (DEGs). In particular, genes with logFC > 0.5 were defined as up-regulated (UP), while with logFC < −0.5 as down-regulated (DOWN).

## 5. Conclusions

In conclusion, the present study provides significant progress on the knowledge of iron metabolism changes in DCM disease, which likely have a clinical meaning. In this regard, the most relevant is that our data show that regulation of intracellular iron homeostasis is dysfunctional in DCM patients’ cardiac tissues, supporting, but not providing, the hypothesis of a direct effect of iron in the disease progression. Indeed, the presence of iron-dependent cell death has been recently identified as a key pathophysiological player in the development of cardiovascular diseases, including HF [57]. Thus, we can suggest why, even in the absence of anemia, both systemic and myocardial ID has a negative role in HF progression in DCM. Indeed, an improvement in iron metabolism at the cardiac level could represent a key mechanism that explains the prognostic benefit of iron supplementation at the systemic level. However, further studies on the regulation of iron metabolism in a comprehensive cohort of DCM patients with and without iron deficiency are needed to infer specific clinical implications.

## Figures and Tables

**Table 1:** Top 15 up- and down-regulated iron-linked genes obtained from bulk RNA-seq analysis in DCM compared to NF group.

	Gene Symbol	Gene Name	logFC	Adj *p* Value
**UP-REGULATED GENES**	*HBA2*	Hemoglobin Subunit Alpha 2	3.7	1.9 × 10^−42^
	*HBA1*	Hemoglobin Subunit Alpha 1	3.5	6.0 × 10^−42^
	*P3H2*	Prolyl 3–Hydroxylase 2	1.6	7.5 × 10^−42^
	*CYP11A1*	Cytochrome P450 Family 11 Subfamily A Member 1	1.6	4.0 × 10^−60^
	*ABCG2*	ATP Binding Cassette Subfamily G Member 2	1.6	4.2 × 10^−53^
	*HAAO*	3–Hydroxyanthranilate 3,4–Dioxygenase	1.3	2.9 × 10^−55^
	*ALOX15*	Arachidonate 15–Lipoxygenase	1.1	2.9 × 10^−15^
	*SNCA*	Synuclein Alpha	1.0	5.8 × 10^−33^
	*CYP4F3*	Cytochrome P450 Family 4 Subfamily F Member 3	1.0	1.3 × 10^−25^
	*P3H3*	Prolyl 3–Hydroxylase 3	1.0	1.1 × 10^−32^
	*CYP2J2*	Cytochrome P450 Family 2 Subfamily J Member 2	0.9	2.0 × 10^−43^
	*ATP6V1G2*	ATPase H+ Transporting V1 Subunit G2	0.9	5.2 × 10^−25^
	*CH25H*	Cholesterol 25–Hydroxylase	0.8	3.2 × 10^−6^
	*HPX*	Hemopexin	0.8	2.9 × 10^−25^
	*ARHGAP1*	Rho GTPase Activating Protein 1	0.7	2.7 × 10^−57^
**DOWN–REGULATED GENES**	*EGLN1*	Egl–9 Family Hypoxia Inducible Factor 1	−0.6	1.6 × 10^−23^
	*CYP1A1*	Cytochrome P450 Family 1 Subfamily A Member 1	−0.6	7.7 × 10^−4^
	*HMOX1*	Heme Oxygenase 1	−0.6	5.7 × 10^−7^
	*TFRC*	Transferrin Receptor	−0.8	6.7 × 10^−19^
	*STEAP4*	STEAP4 Metalloreductase	−0.9	1.2 × 10^−26^
	*STEAP3*	STEAP3 Metalloreductase	−0.9	6.4 × 10^−48^
	*HMOX2*	Heme Oxygenase 2	−0.9	4.8 × 10^−67^
	*ALOX5*	Arachidonate 5–Lipoxygenase	−0.9	2.7 × 10^−29^
	*NECTIN1*	Nectin Cell Adhesion Molecule 1	−1.0	1.1 × 10^−38^
	*SLC11A1*	Solute Carrier Family 11 Member 1	−1.4	1.6 × 10^−35^
	*PPEF1*	Protein Phosphatase With EF-Hand Domain 1	−1.8	1.1 × 10^−36^
	*CYP4Z1*	Cytochrome P450 Family 4 Subfamily Z Memb 1	−2.2	6.4 × 10^−54^
	*CYP4B1*	Cytochrome P450 Family 4 Subfamily B Memb 1	−2.3	2.0 × 10^−48^
	*AOX1*	Aldehyde Oxidase 1	−2.3	1.7 × 10^−59^
	*ALOX15B*	Arachidonate 15–Lipoxygenase Type B	−2.6	4.9 × 10^−36^

**Table 2:** Regulation type of main players in the iron metabolism across the five cell populations.

Gene	Function	Cardiomyocytes	Fibroblasts	Myeloid Cells	Endocardial Cells	Endothelial Cells
*ABCG2*	Exports of heme from cells	↑	↑	↑	↑	−
*ALAS2*	The rate-limiting enzyme in heme synthesis	↑	−	−	−	−
*BMP6*	Major mediator in the negative feedback loop between iron and hepcidin	↑	↑	↑	↑	−
*CYBRD1*	Enzymatically reduces Fe^3+^ to Fe^2+^ using ascorbate (vitamin C) as an electron donor	−	−	−	↑	−
*FLVCR2*	Imports heme into the cell	−	↓	−	−	−
*HBA1/2*	Constitutes hemoglobin	↑	↑	↑	↑	↑
*HMOX1/2*	Degrades heme into iron, carbon monoxide, and biliverdin	↓	↓	−	−	−
*NEO1*	Associates with the BMP receptor complex via BMP receptor type Ia	−	↑	−	−	−
*SCARA5*	Binds and internalizes L-ferritin	↓	↓	↓	−	↓
*SLC25A28*	Inner membrane transporter of Fe^2+^ into mitochondria	↑	−	−	−	↑
*SLC39A8*	Takes up unbound Fe^2+^ into the cell	−	−	↓	−	−
*SLC48A1*	Transports heme across the endosomal membrane	−	↓	−	↓	−
*STEAP3/4*	Transmembrane proteins with 4 homologous members	↓	−	↓	↓	↓
*TFRC*	Upon internalization of the Tf-TfR complex by endocytosis	−	↓	−	−	−

Gene symbols were reported together with their function. The upward arrow indicates up-regulation, the downward arrow indicates down-regulation, while the dash means no significant regulation. ATP-binding cassette super-family G member 2 (*ABCG2*), delta-aminolevulinic synthase 2 (*ALAS2*), bone morphogenetic protein 6 (*BMP6*), cytochrome B reductase 1 (*CYBRD1*), feline leukemia virus subgroup C receptor 2 (*FLVCR2*), alpha-globin 1/2 (*HBA1/2*), heme oxygenase 1/2 (*HMOX1/2*), neogenin 1 (*NEO1*), scavenger receptor class A member 5 (*SCARA5*), solute carrier family 25 member 28 (*SLC25A28*), solute carrier family 39 member 8 (*SLC39A8*), solute carrier family 48 member 1 (*SLC48A1*), six-transmembrane epithelial antigen of the prostate 3/4 (*STEAP3/4*), transferrin Receptor (*TFRC*).

## Data Availability

Data is contained in the manuscript and in the supplementary materials.

## Supplementary Materials

The following supporting information can be downloaded at: https://www.mdpi.com/article/10.3390/ijms24032887/s1.

## References

[1] Seferović P.M., Polovina M., Bauersachs J., Arad M., Ben Gal T., Lund L.H., Felix S.B., Arbustini E., Caforio A.L.P., Farmakis D. (2019). Heart failure in cardiomyopathies: A position paper from the Heart Failure Association of the European Society of Cardiology. Eur. J. Heart Fail..

[2] Visseren F.L.J., Mach F., Smulders Y.M., Carballo D., Koskinas K.C., Back M., Benetos A., Biffi A., Boavida J.M., Capodanno D. (2021). 2021 ESC Guidelines on cardiovascular disease prevention in clinical practice. Eur. Heart J..

[3] Miyagawa S., Domae K., Kainuma S., Matsuura R., Yoshioka D., Hata H., Yoshikawa Y., Toda K., Sawa Y. (2018). Long-term outcome of a dilated cardiomyopathy patient after mitral valve surgery combined with tissue-engineered myoblast sheets—Report of a case. Surg. Case Rep..

[4] Tenge T., Roth S., M‘Pembele R., Buse G.L., Boenner F., Ballázs C., Tudorache I., Boeken U., Lichtenberg A., Neukirchen M. (2022). Impact of Left Ventricular Assist Devices on Days Alive and Out of Hospital in Hemodynamically Stable Patients with End-Stage Heart Failure: A Propensity Score Matched Study. Life.

[5] Japp A.G., Gulati A., Cook S.A., Cowie M.R., Prasad S.K. (2016). The Diagnosis and Evaluation of Dilated Cardiomyopathy. J. Am. Coll. Cardiol..

[6] Jankowska E.A., Rozentryt P., Witkowska A., Nowak J., Hartmann O., Ponikowska B., Borodulin-Nadzieja L., Banasiak W., Polonski L., Filippatos G. (2010). Iron deficiency: An ominous sign in patients with systolic chronic heart failure. Eur. Heart J..

[7] Murphy C.J., Oudit G.Y. (2010). Iron-Overload Cardiomyopathy: Pathophysiology, Diagnosis, and Treatment. J. Card. Fail..

[8] Loncar G., Obradovic D., Thiele H., von Haehling S., Lainscak M. (2021). Iron deficiency in heart failure. ESC Heart Fail..

[9] Zhang H., Zhabyeyev P., Wang S., Oudit G.Y. (2019). Role of iron metabolism in heart failure: From iron deficiency to iron overload. Biochim. Biophys. Acta Mol. Basis Dis..

[10] Dev S., Babitt J.L. (2017). Overview of iron metabolism in health and disease. Hemodial. Int..

[11] Vogt A.-C., Arsiwala T., Mohsen M., Vogel M., Manolova V., Bachmann M. (2021). On Iron Metabolism and Its Regulation. Int. J. Mol. Sci..

[12] Mackenzie E.L., Iwasaki K., Tsuji Y. (2008). Intracellular Iron Transport and Storage: From Molecular Mechanisms to Health Implications. Antioxid. Redox Signal..

[13] Muckenthaler M.U., Rivella S., Hentze M.W., Galy B. (2017). A Red Carpet for Iron Metabolism. Cell.

[14] Jomova K., Valko M. (2011). Advances in metal-induced oxidative stress and human disease. Toxicology.

[15] Sukhbaatar N., Weichhart T. (2018). Iron Regulation: Macrophages in Control. Pharmaceuticals.

[16] Kozłowska B., Sochanowicz B., Kraj L., Palusińska M., Kołsut P., Szymański Ł., Lewicki S., Kruszewski M., Załęska-Kocięcka M., Leszek P. (2022). Clinical and Molecular Aspects of Iron Metabolism in Failing Myocytes. Life.

[17] Ghafourian K., Shapiro J.S., Goodman L., Ardehali H. (2020). Iron and Heart Failure: Diagnosis, Therapies, and Future Directions. JACC Basic Transl. Sci..

[18] Ravingerová T., Kindernay L., Barteková M., Ferko M., Adameová A., Zohdi V., Bernátová I., Ferenczyová K., Lazou A. (2020). The Molecular Mechanisms of Iron Metabolism and Its Role in Cardiac Dysfunction and Cardioprotection. Int. J. Mol. Sci..

[19] Zhang D.-L., Ghosh M.C., Rouault T.A. (2014). The physiological functions of iron regulatory proteins in iron homeostasis—An update. Front. Pharmacol..

[20] Anand I.S., Gupta P. (2018). Anemia and Iron Deficiency in Heart Failure: Current Concepts and Emerging Therapies. Circulation.

[21] McDonagh T., Damy T., Doehner W., Lam C.S., Sindone A., Van Der Meer P., Cohen-Solal A., Kindermann I., Manito N., Pfister O. (2018). Screening, diagnosis and treatment of iron deficiency in chronic heart failure: Putting the 2016 European Society of Cardiology heart failure guidelines into clinical practice. Eur. J. Heart Fail..

[22] Campodonico J., Nicoli F., Motta I., De Amicis M.M., Bonomi A., Cappellini M., Agostoni P. (2021). Prognostic role of transferrin saturation in heart failure patients. Eur. J. Prev. Cardiol..

[23] Anker S.D., Comin Colet J., Filippatos G., Willenheimer R., Dickstein K., Drexler H., Lüscher T.F., Bart B., Banasiak W., Niegowska J. (2009). Ferric Carboxymaltose in Patients with Heart Failure and Iron Deficiency. N. Engl. J. Med..

[24] Caravita S., Faini A., Vignati C., Pelucchi S., Salvioni E., Cattadori G., Baratto C., Torlasco C., Contini M., Villani A. (2022). Intravenous iron therapy improves the hypercapnic ventilatory response and sleep disordered breathing in chronic heart failure. Eur. J. Heart Fail..

[25] Jankowska E.A., Kirwan B.-A., Kosiborod M., Butler J., Anker S.D., McDonagh T., Dorobantu M., Drozdz J., Filippatos G., Keren A. (2011). The effect of intravenous ferric carboxymaltose on health-related quality of life in iron-deficient patients with acute heart failure: The results of the AFFIRM-AHF study. Eur. Heart J..

[26] Zhang H., Jamieson K.L., Grenier J., Nikhanj A., Tang Z., Wang F., Wang S., Seidman J.G., Seidman C.E., Thompson R. (2022). Myocardial Iron Deficiency and Mitochondrial Dysfunction in Advanced Heart Failure in Humans. J. Am. Heart Assoc..

[27] Alnuwaysir R.I.S., Hoes M.F., van Veldhuisen D.J., van der Meer P., Beverborg N.G. (2021). Iron Deficiency in Heart Failure: Mechanisms and Pathophysiology. J. Clin. Med..

[28] Berdoukas V., Coates T.D., Cabantchik Z.I. (2015). Iron and oxidative stress in cardiomyopathy in thalassemia. Free. Radic. Biol. Med..

[29] Fang X., Ardehali H., Min J., Wang F. (2022). The molecular and metabolic landscape of iron and ferroptosis in cardiovascular disease. Nat. Rev. Cardiol..

[30] Ayer A., Zarjou A., Agarwal A., Stocker R. (2016). Heme Oxygenases in Cardiovascular Health and Disease. Physiol. Rev..

[31] Zhang H., Huo J., Jiang W., Shan Q. (2020). Integrated microarray analysis to identify potential biomarkers and therapeutic targets in dilated cardiomyopathy. Mol. Med. Rep..

[32] Liberzon A., Birger C., Thorvaldsdottir H., Ghandi M., Mesirov J.P., Tamayo P. (2015). The Molecular Signatures Database (MSigDB) hallmark gene set collection. Cell Syst..

[33] Koenig A.L., Shchukina I., Amrute J., Andhey P.S., Zaitsev K., Lai L., Bajpai G., Bredemeyer A., Smith G., Jones C. (2022). Single-cell transcriptomics reveals cell-type-specific diversification in human heart failure. Nat. Cardiovasc. Res..

[34] Zheng Y., Liu Z., Yang X., Weng S., Xu H., Guo C., Xing Z., Liu L., Wang L., Dang Q. (2022). Exploring Key Genes to Construct a Diagnosis Model of Dilated Cardiomyopathy. Front. Cardiovasc. Med..

[35] Ohgami R.S., Campagna D.R., Greer E.L., Antiochos B., McDonald A., Chen J., Sharp J.J., Fujiwara Y., Barker J.E., Fleming M.D. (2005). Identification of a ferrireductase required for efficient transferrin-dependent iron uptake in erythroid cells. Nat. Genet..

[36] Maeder M.T., Khammy O., dos Remedios C., Kaye D.M. (2011). Myocardial and Systemic Iron Depletion in Heart Failure: Implications for Anemia Accompanying Heart Failure. J. Am. Coll. Cardiol..

[37] Li P.-L., Liu H., Chen G.-P., Li L., Shi H.-J., Nie H.-Y., Liu Z., Hu Y.-F., Yang J., Zhang P. (2020). STEAP3 (Six-Transmembrane Epithelial Antigen of Prostate 3) Inhibits Pathological Cardiac Hypertrophy. Hypertension.

[38] Kumar S., Bandyopadhyay U. (2005). Free heme toxicity and its detoxification systems in human. Toxicol. Lett..

[39] Son G.H., Park S.H., Kim Y., Kim J.Y., Kim J.W., Chung S., Kim Y.-H., Kim H., Hwang J.-J., Seo J.-S. (2014). Postmortem mRNA expression patterns in left ventricular myocardial tissues and their implications for forensic diagnosis of sudden cardiac death. Mol. Cells.

[40] Khechaduri A., Bayeva M., Chang H.-C., Ardehali H. (2013). Heme Levels Are Increased in Human Failing Hearts. J. Am. Coll. Cardiol..

[41] Bellner L., Martinelli L., Halilovic A., Patil K., Puri N., Dunn M.W., Regan R.F., Schwartzman M.L. (2009). Heme Oxygenase-2 Deletion Causes Endothelial Cell Activation Marked by Oxidative Stress, Inflammation, and Angiogenesis. J. Pharmacol. Exp. Ther..

[42] He J.Z., Ho J.J.D., Gingerich S., Courtman D.W., Marsden P.A., Ward M.E. (2010). Enhanced Translation of Heme Oxygenase-2 Preserves Human Endothelial Cell Viability during Hypoxia. J. Biol. Chem..

[43] Lundvig D.M., Scharstuhl A., Cremers N.A., Pennings S.W., te Paske J., van Rheden R., van Run-van Breda C., Regan R.F., Russel F.G., Carels C.E. (2014). Delayed cutaneous wound closure in HO-2 deficient mice despite normal HO-1 expression. J. Cell. Mol. Med..

[44] Andriopoulos B., Corradini E., Xia Y., Faasse S.A., Chen S., Grgurevic L., Knutson M.D., Pietrangelo A., Vukicevic S., Lin H.Y. (2009). BMP6 is a key endogenous regulator of hepcidin expression and iron metabolism. Nat. Genet..

[45] Vela D. (2018). Balance of cardiac and systemic hepcidin and its role in heart physiology and pathology. Lab. Investig..

[46] Fang X., Cai Z., Wang H., Han D., Cheng Q., Zhang P., Gao F., Yu Y., Song Z., Wu Q. (2020). Loss of Cardiac Ferritin H Facilitates Cardiomyopathy via Slc7a11-Mediated Ferroptosis. Circ. Res..

[47] Yuan H., Li X., Zhang X., Kang R., Tang D. (2016). CISD1 inhibits ferroptosis by protection against mitochondrial lipid peroxidation. Biochem. Biophys. Res. Commun..

[48] Kim E.H., Shin D., Lee J., Jung A.R., Roh J.-L. (2018). CISD2 inhibition overcomes resistance to sulfasalazine-induced ferroptotic cell death in head and neck cancer. Cancer Lett..

[49] Zhang Z., Guo M., Shen M., Kong D., Zhang F., Shao J., Tan S., Wang S., Chen A., Cao P. (2020). The BRD7-P53-SLC25A28 axis regulates ferroptosis in hepatic stellate cells. Redox Biol..

[50] Lachmann A., Torre D., Keenan A.B., Jagodnik K.M., Lee H.J., Wang L., Silverstein M.C., Ma’Ayan A. (2018). Massive mining of publicly available RNA-seq data from human and mouse. Nat. Commun..

[51] Edgar R., Domrachev M., Lash A.E. (2002). Gene Expression Omnibus: NCBI gene expression and hybridization array data repository. Nucleic Acids Res..

[52] Sciomer S., Rellini C., Agostoni P., Moscucci F. (2020). A new pathophysiology in heart failure patients. Artif. Organs.

[53] Chiesa M., Colombo G.I., Piacentini L. (2018). DaMiRseq-an R/Bioconductor package for data mining of RNA-Seq data: Normalization, feature selection and classification. Bioinformatics.

[54] Wang L., Yu P., Zhou B., Song J., Li Z., Zhang M., Guo G., Wang Y., Chen X., Han L. (2020). Single-cell reconstruction of the adult human heart during heart failure and recovery reveals the cellular landscape underlying cardiac function. Nat. Cell Biol..

[55] Ringner M. (2008). What is principal component analysis?. Nat. Biotechnol..

[56] Ritchie M.E., Phipson B., Wu D., Hu Y., Law C.W., Shi W., Smyth G.K. (2015). limma powers differential expression analyses for RNA-sequencing and microarray studies. Nucleic Acids Res..

[57] Yu Y., Yan Y., Niu F., Wang Y., Chen X., Su G., Liu Y., Zhao X., Qian L., Liu P. (2021). Ferroptosis: A cell death connecting oxidative stress, inflammation and cardiovascular diseases. Cell Death Discov..

