# Supplementary material for: Dysregulation of Iron Metabolism-Linked Genes at Myocardial Tissue and Cell Levels in Dilated Cardiomyopathy
Source: Int J Mol Sci. 2023 Feb 2;24(3):2887. doi: 10.3390/ijms24032887 (PMC9918212; doi:10.3390/ijms24032887)
Supplement: Supplementary file 1 [file ijms-24-02887-s001.zip › ijms-2149978-supplementary.pdf]

Supplementary Materials

Supplementary Figures

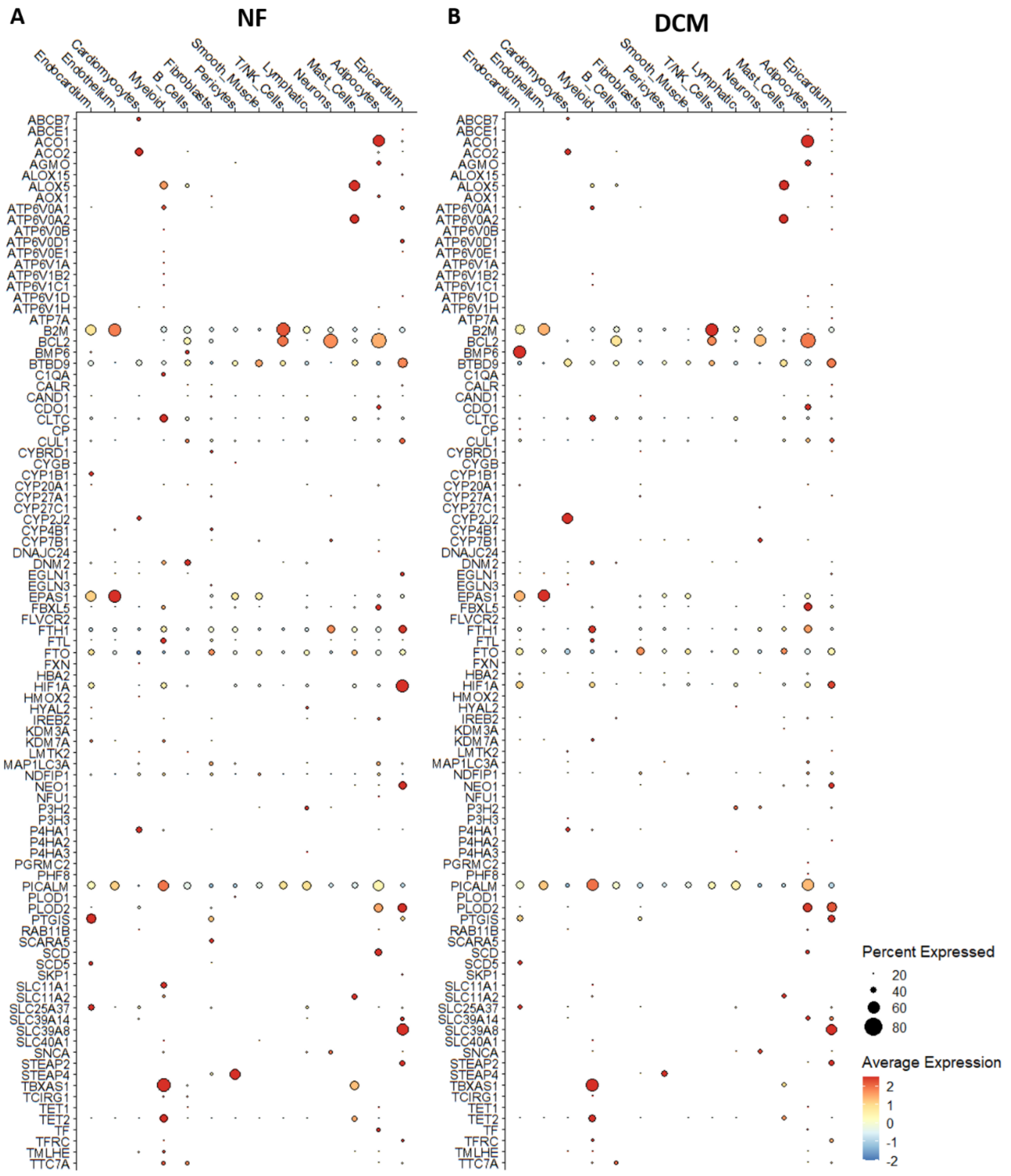

Supplementary Figure S1. Dotplot representations of the gene expression iron linked-genes across each cell type. The size of the dots encodes the percentage of cells expressing a gene (≥ 20%), while the color encodes the average expression level across all cells within a class. (**A**) Expression levels of iron linked-genes for the cell types in donor (NF). (**B**) Expression levels of iron linked-genes for the cell types in dilated cardiomyopathy (DCM) group. Among the 272 total iron linked-genes, just those with a percentage of expression ≥ 20% in at least one cell type of CTRL or DCM group were represented.

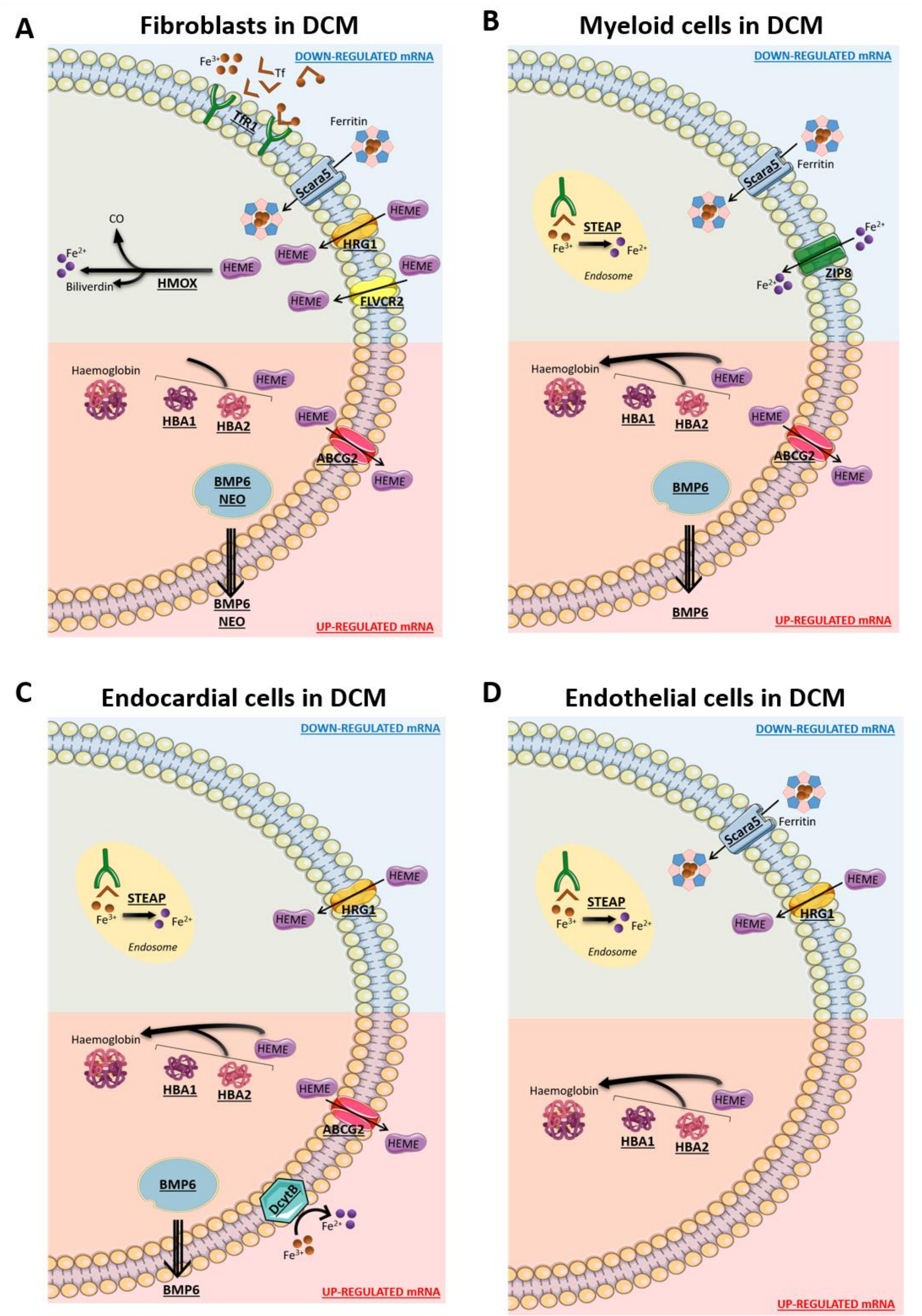

Supplementary Figure S2. Iron-linked affected genes in dilated cardiomyopathy (DCM) for four cell types. (**A**) Up- and down-regulated proteins/genes in DCM fibroblasts. (**B**) Up- and down-regulated proteins/genes in DCM myeloid cells. (**C**) Up- and down-regulated proteins/genes in DCM endocardial cells. (**D**) Up- and down-regulated proteins/genes in DCM endothelial cells.

Supplementary Tables

Table S1. Differentially expressed genes obtained from the comparison of DCM and NF bulk RNA-seq data. pValue was adjusted using Benjamini-Hochberg method.

Gene name	logFC	Average Expression	pValue	adj pValue
HBA2	3.68	1.83	2.54E-44	1.94E-42
SEZ6L	3.55	-0.45	3.39E-100	1.42E-96
SFRP4	3.54	2.56	8.42E-94	2.01E-90
HBA1	3.53	0.78	8.02E-44	6.05E-42
HBB	3.49	3.94	1.13E-54	1.84E-52
PENK	3.35	0.72	9.42E-74	4.38E-71
COL22A1	3.34	-1.09	8.59E-78	6.25E-75
FNDC1	3.07	1.91	1.54E-112	1.29E-108
NPPA	3.00	8.06	7.78E-38	3.82E-36
HAPLN1	2.85	1.03	2.62E-54	4.07E-52
LEFTY2	2.84	-0.33	3.36E-83	3.75E-80
TNMD	2.72	-0.77	5.06E-49	5.51E-47
MXRA5	2.71	3.99	9.31E-68	3.32E-65
APCDD1L	2.70	-1.27	4.37E-69	1.59E-66
FCER1A	2.68	-0.53	5.52E-61	1.29E-58
CRISPLD1	2.64	0.56	3.71E-62	9.70E-60
LYPD1	2.55	-1.90	1.70E-50	2.04E-48
FHAD1	2.50	-0.70	1.32E-97	3.68E-94
GZMH	2.45	-1.86	3.27E-60	7.41E-58
CXCL10	2.43	-0.78	7.37E-37	3.28E-35
KIAA1211	2.40	-2.28	2.79E-46	2.39E-44
LAMP5	2.38	-2.59	2.85E-71	1.22E-68
ARMS2	2.38	-2.42	2.26E-77	1.58E-74
CENPA	2.38	0.70	6.58E-37	2.95E-35
NPPB	2.37	6.09	5.66E-21	7.78E-20
FRZB	2.30	2.99	5.27E-99	1.76E-95
ASPN	2.29	5.31	2.43E-70	9.71E-68
DNAAF3	2.28	3.51	3.58E-78	2.72E-75
LUM	2.27	7.30	1.82E-79	1.69E-76
AQP10	2.23	-2.20	3.03E-74	1.49E-71
CMA1	2.21	-1.70	3.62E-45	2.88E-43
WNT9A	2.21	2.73	1.07E-47	1.07E-45
ZCCHC5	2.18	-1.13	1.06E-61	2.57E-59
ESM1	2.17	-1.74	1.42E-37	6.68E-36
NKG7	2.15	-0.91	2.13E-47	2.03E-45
COMP	2.13	1.31	8.46E-26	1.70E-24
AGTR2	2.13	-2.01	1.02E-40	6.17E-39
SCUBE2	2.12	1.65	2.10E-79	1.85E-76
CD1C	2.12	-0.75	1.06E-45	8.75E-44
PI16	2.10	4.29	6.96E-62	1.71E-59
ANKRD34C	2.07	-0.06	1.83E-53	2.73E-51
PLD4	2.06	-2.49	1.06E-63	3.05E-61
ACE	2.06	2.50	2.47E-69	9.21E-67
SSC5D	2.06	2.22	5.44E-87	9.11E-84
OGN	2.04	4.68	1.40E-58	2.97E-56
CX3CR1	2.04	-0.34	2.82E-45	2.28E-43
HNRNPA1P66	2.04	-2.35	2.47E-41	1.52E-39
TRIL	2.04	1.95	4.01E-77	2.59E-74
TNNI1	2.00	3.24	3.28E-26	6.72E-25
GLIS1	2.00	-2.55	1.79E-47	1.73E-45
SLC16A9	1.99	1.93	4.34E-72	1.96E-69
SMOC2	1.99	5.55	1.18E-113	1.98E-109
GDF6	1.98	-0.42	9.27E-50	1.06E-47
MDK	1.98	1.32	1.14E-57	2.35E-55
TRBC1	1.97	-1.10	3.02E-45	2.42E-43
CTSG	1.96	-0.96	9.75E-41	5.89E-39
APOA1	1.96	4.27	1.94E-40	1.14E-38
SAMD11	1.95	-0.80	7.77E-36	3.22E-34
OGDHL	1.93	1.80	1.85E-57	3.69E-55
XG	1.92	-0.48	1.58E-35	6.38E-34
RGS4	1.91	1.30	5.63E-45	4.45E-43
CHRNA3	1.89	-1.23	2.29E-42	1.54E-40
FREM1	1.89	2.73	4.10E-92	8.57E-89
SYTL5	1.86	-0.61	6.67E-25	1.25E-23
ISLR	1.86	3.71	4.51E-63	1.26E-60
CXCL11	1.85	-2.46	1.12E-25	2.23E-24
COL14A1	1.84	4.88	1.67E-54	2.66E-52
SLC1A7	1.82	0.19	2.75E-52	3.68E-50
NRG1	1.82	-0.78	5.45E-34	1.98E-32
FMOD	1.82	5.15	1.59E-48	1.70E-46
CCL5	1.82	0.94	1.26E-54	2.03E-52
CXCL9	1.82	0.44	9.94E-23	1.58E-21

ATRNL1	1.81	1.39	2.76E-33	9.44E-32
GZMB	1.80	-1.88	2.11E-39	1.15E-37
STAT4	1.79	2.37	1.06E-42	7.30E-41
AEBP1	1.79	5.59	6.24E-52	8.22E-50
COL9A1	1.76	0.03	2.01E-54	3.18E-52
CYS1	1.75	1.43	2.50E-84	2.99E-81
ATP1B4	1.75	0.04	1.16E-27	2.65E-26
FATE1	1.75	-2.55	1.41E-40	8.43E-39
FAP	1.75	2.21	5.51E-40	3.15E-38
GZMA	1.75	-0.77	2.23E-41	1.38E-39
PRSS35	1.74	-2.08	1.35E-35	5.48E-34
GAP43	1.74	0.17	1.26E-27	2.88E-26
NRXN2	1.74	-0.72	2.83E-47	2.62E-45
APLNR	1.74	3.54	4.26E-47	3.88E-45
VGLL2	1.73	-0.87	3.34E-19	3.95E-18
MFAP4	1.73	5.74	1.98E-81	2.07E-78
LSP1	1.73	3.80	5.58E-62	1.42E-59
FCGR3B	1.72	0.53	1.33E-30	3.74E-29
C1QTNF7	1.72	1.86	5.42E-72	2.39E-69
PRF1	1.72	-0.31	6.23E-47	5.61E-45
MKRN2OS	1.71	0.27	3.98E-34	1.46E-32
NRK	1.71	2.89	1.01E-64	3.14E-62
RP11-481H12.1	1.70	-2.49	2.33E-39	1.26E-37
LTB	1.68	-2.35	5.90E-24	1.02E-22
OXER1	1.68	-1.11	4.25E-57	8.38E-55
MYOC	1.68	2.82	8.29E-36	3.42E-34
LRRC15	1.68	-1.45	1.75E-30	4.90E-29
PRKAG3	1.67	-2.41	8.60E-48	8.63E-46
C16orf89	1.67	2.05	1.95E-51	2.49E-49
PHLDA1	1.66	5.01	2.04E-66	6.69E-64
SHISA2	1.65	-1.52	1.23E-42	8.41E-41
PTN	1.65	4.87	2.75E-70	1.07E-67
NTM	1.65	0.82	3.47E-65	1.12E-62
GSTM5	1.64	1.17	5.07E-50	5.94E-48
TLL2	1.64	3.32	2.37E-89	4.41E-86
MYL1	1.64	-2.05	6.51E-27	1.40E-25
OASL	1.64	-0.54	2.21E-47	2.09E-45
CERS1	1.63	-1.23	8.15E-46	6.79E-44
RASL11B	1.63	3.08	1.28E-55	2.25E-53
C1QTNF2	1.62	0.79	2.53E-76	1.37E-73
LRRC17	1.62	2.19	2.72E-55	4.65E-53
P3H2	1.61	2.83	1.01E-43	7.48E-42
TMEM119	1.61	1.16	1.96E-34	7.37E-33
C2orf40	1.60	0.54	5.19E-52	6.90E-50
TMEM200A	1.60	-2.39	1.71E-24	3.09E-23
TMEM30B	1.60	0.18	1.78E-43	1.28E-41
ITGBL1	1.60	2.55	9.40E-32	2.83E-30
SIGLEC17P	1.59	-1.73	1.59E-36	6.95E-35
CYP11A1	1.59	-0.11	1.47E-62	4.03E-60
THY1	1.59	1.84	1.74E-23	2.92E-22
HRK	1.58	-0.89	3.06E-42	2.02E-40
ZAP70	1.57	-0.73	1.62E-39	8.91E-38
CA3	1.56	3.15	1.04E-23	1.77E-22
ABCG2	1.56	1.94	2.41E-55	4.16E-53
CD3D	1.56	-1.62	8.50E-32	2.57E-30
PROM1	1.56	2.74	4.63E-55	7.76E-53
HDC	1.56	-0.47	1.17E-32	3.79E-31
ADAMTS14	1.55	-0.57	8.75E-35	3.37E-33
SCG2	1.55	-1.06	3.71E-19	4.37E-18
CD3E	1.55	-0.60	4.01E-36	1.70E-34
KLRB1	1.55	-1.30	3.19E-34	1.17E-32
OPCML	1.55	-1.70	1.62E-34	6.11E-33
EGR2	1.55	0.85	1.89E-26	3.94E-25
SCG5	1.54	-2.35	6.59E-28	1.54E-26
KCNK17	1.54	-2.24	8.32E-29	2.06E-27
COL10A1	1.53	-1.79	1.39E-25	2.76E-24
COL16A1	1.53	4.66	1.70E-56	3.28E-54
SLAMF7	1.53	-0.76	2.54E-33	8.73E-32
LCN12	1.53	0.90	4.60E-42	3.02E-40
CHRDL1	1.52	2.45	2.61E-24	4.63E-23
SDSL	1.52	2.43	1.42E-47	1.41E-45
LHCGR	1.51	-1.95	4.84E-22	7.27E-21
CXCL14	1.50	1.05	5.57E-26	1.13E-24
CD1E	1.50	-2.37	2.66E-26	5.48E-25
COL8A2	1.50	0.79	1.00E-48	1.09E-46
CTSW	1.49	-1.09	3.06E-35	1.21E-33
GFRA3	1.49	-0.57	6.06E-39	3.18E-37
IL34	1.49	-0.09	7.30E-48	7.41E-46
CD2	1.49	-0.46	1.01E-31	3.02E-30
DIO2	1.48	4.07	2.48E-56	4.71E-54
MYOZ1	1.48	0.89	6.52E-36	2.71E-34
LRRC55	1.48	-1.36	2.49E-33	8.59E-32
ITIH5	1.48	4.82	8.34E-86	1.27E-82

TGFB2	1.47	2.83	2.81E-32	8.85E-31
MOXD1	1.47	2.52	5.38E-51	6.67E-49
ARMC4	1.47	-1.75	2.70E-39	1.44E-37
BEX1	1.47	1.78	2.77E-27	6.19E-26
TBX21	1.46	-2.38	5.55E-32	1.69E-30
EFCC1	1.46	1.28	1.94E-58	4.06E-56
MATN2	1.46	5.04	9.73E-77	5.82E-74
CPXM2	1.45	3.21	1.58E-42	1.07E-40
ITGAL	1.45	0.89	4.48E-45	3.55E-43
GABRD	1.44	-2.17	2.26E-29	5.87E-28
ARMCX7P	1.44	-0.78	8.95E-07	3.01E-06
SCARA3	1.44	2.06	9.04E-61	2.07E-58
TNFRSF4	1.43	-0.62	3.36E-18	3.65E-17
IGHG1	1.43	2.99	1.22E-11	7.20E-11
S1PR5	1.43	-2.56	7.58E-31	2.17E-29
COL1A1	1.43	6.03	4.93E-25	9.32E-24
KIAA1755	1.43	2.44	2.07E-55	3.60E-53
FAM180B	1.42	-1.73	3.94E-37	1.80E-35
GSG1L	1.42	0.24	7.39E-19	8.48E-18
DACT2	1.41	-1.02	9.39E-21	1.27E-19
CRHBP	1.41	0.66	9.20E-43	6.42E-41
TRGC2	1.41	-2.46	4.55E-27	9.98E-26
RP11-1094M14.8	1.41	-2.65	7.31E-33	2.41E-31
BPI	1.40	-2.50	3.78E-31	1.10E-29
THBS4	1.40	7.51	5.63E-38	2.81E-36
SARDH	1.40	-1.06	4.32E-47	3.91E-45
SCN2B	1.40	4.40	1.50E-63	4.25E-61
UCHL1	1.40	3.07	8.95E-30	2.39E-28
P2RX6	1.40	1.02	1.74E-34	6.57E-33
LTBP2	1.40	6.56	7.99E-40	4.51E-38
DPT	1.40	6.22	5.07E-51	6.34E-49
ANKRD24	1.39	-1.44	3.67E-33	1.24E-31
SOX8	1.39	0.69	1.33E-49	1.51E-47
LEF1	1.39	-0.95	1.71E-31	5.06E-30
AZIN2	1.39	1.71	3.74E-64	1.12E-61
NGEF	1.38	-2.26	5.28E-24	9.20E-23
LTC4S	1.38	-1.36	1.12E-19	1.39E-18
EXOC3L4	1.38	-1.76	7.80E-29	1.93E-27
CRABP2	1.38	-0.65	8.45E-29	2.09E-27
IRX6	1.37	3.53	5.89E-36	2.48E-34
FAM133A	1.37	-1.22	1.10E-21	1.61E-20
CTHRC1	1.37	0.99	8.88E-26	1.78E-24
PRRT2	1.37	1.07	6.17E-48	6.38E-46
CRYM	1.37	6.49	1.10E-67	3.85E-65
IL2RB	1.36	-0.85	2.69E-34	9.99E-33
SERPINE2	1.36	5.10	3.03E-34	1.12E-32
CD247	1.36	-0.83	2.48E-39	1.33E-37
PTPRCAP	1.35	-2.05	8.54E-11	4.62E-10
PLEKHH2	1.35	2.82	1.62E-36	7.07E-35
FERMT1	1.35	-0.42	8.55E-32	2.58E-30
PADI4	1.34	-1.62	2.44E-21	3.48E-20
MAP3K7CL	1.34	2.42	2.38E-23	3.96E-22
HAAO	1.34	0.92	1.40E-57	2.86E-55
IFI44L	1.34	4.18	1.95E-44	1.51E-42
IL31RA	1.34	-2.35	3.50E-19	4.13E-18
FOXS1	1.34	0.61	7.18E-22	1.06E-20
EIF1AY	1.34	3.57	0.001530151	0.003181323
ADAMTSL1	1.33	0.65	1.62E-30	4.55E-29
METTL21EP	1.33	1.87	1.52E-36	6.67E-35
PALM3	1.33	-2.53	8.59E-32	2.59E-30
MRC2	1.33	4.82	3.07E-40	1.78E-38
TENM4	1.33	0.65	2.06E-46	1.79E-44
HAPLN4	1.33	-2.71	2.09E-33	7.22E-32
CD83	1.33	0.99	1.96E-47	1.89E-45
SPNS3	1.32	-1.62	2.94E-30	8.09E-29
CCDC80	1.32	7.42	8.13E-34	2.93E-32
ZMYND15	1.32	-0.97	6.08E-40	3.45E-38
DMC1	1.32	0.02	2.80E-54	4.31E-52
IGHG2	1.32	1.03	6.18E-11	3.40E-10
RPS4Y1	1.32	3.13	0.003717404	0.00718018
NT5E	1.32	2.76	2.21E-52	2.99E-50
COL9A2	1.32	-0.59	1.57E-37	7.35E-36
WNT10B	1.32	-2.08	3.02E-27	6.74E-26
TMEM233	1.32	-0.03	8.68E-37	3.84E-35
IGLC1	1.32	0.18	1.11E-10	5.96E-10
SLC6A1	1.31	1.93	5.60E-42	3.64E-40
ETV4	1.31	0.09	8.66E-38	4.24E-36
LMX1A	1.31	-2.60	1.00E-27	2.32E-26
MALRD1	1.31	-1.96	6.21E-22	9.26E-21
NUP62CL	1.31	-0.32	9.01E-41	5.47E-39
FAM212A	1.30	-0.26	2.52E-29	6.50E-28
UBD	1.30	-2.87	3.54E-14	2.67E-13
MSS51	1.30	2.86	4.18E-53	5.98E-51

GDF10	1.30	-0.81	3.36E-26	6.88E-25
CLEC4F	1.29	-1.38	7.24E-21	9.88E-20
NAP1L3	1.29	2.65	1.13E-35	4.62E-34
RHCG	1.29	-1.99	5.81E-16	5.16E-15
IGKV3-15	1.29	-1.91	1.48E-09	7.00E-09
PPDPF	1.29	5.41	1.04E-25	2.07E-24
IGHM	1.29	0.28	1.12E-12	7.40E-12
SKAP1	1.29	-1.60	8.69E-30	2.33E-28
AK5	1.28	-0.69	4.34E-46	3.69E-44
SULF1	1.28	4.22	4.91E-42	3.21E-40
DLGAP1	1.28	-0.72	1.12E-27	2.57E-26
GNLY	1.28	0.51	3.45E-36	1.48E-34
IGHA1	1.27	1.90	1.97E-10	1.03E-09
DUOX2	1.27	-0.41	6.17E-30	1.66E-28
MME	1.27	3.42	1.03E-37	4.94E-36
ECM2	1.27	4.15	6.00E-36	2.52E-34
IGKC	1.27	3.86	1.64E-11	9.54E-11
PHF21B	1.26	-2.06	2.90E-32	9.11E-31
FBLN7	1.26	-0.91	1.08E-39	6.03E-38
PIK3R6	1.26	0.53	2.17E-45	1.78E-43
CHD5	1.26	-1.56	6.65E-29	1.65E-27
F2RL2	1.26	0.60	8.41E-26	1.69E-24
EIF4EBP3	1.26	3.09	4.48E-31	1.29E-29
SH2D1A	1.26	-2.35	4.10E-22	6.20E-21
CLEC11A	1.26	0.99	3.20E-28	7.64E-27
PRPH	1.25	-2.16	4.14E-26	8.45E-25
RXRG	1.25	2.01	4.00E-35	1.56E-33
CCL4	1.24	-0.84	1.36E-21	1.97E-20
LINC01330	1.24	2.67	7.20E-37	3.21E-35
IGLC2	1.24	1.80	1.65E-10	8.68E-10
FCER2	1.24	-1.80	1.01E-20	1.36E-19
CARD11	1.24	-1.63	3.15E-28	7.55E-27
DUSP15	1.24	0.17	6.61E-42	4.24E-40
C1QTNF9	1.24	0.94	2.34E-34	8.73E-33
KCNN3	1.24	2.35	8.22E-56	1.48E-53
CTSK	1.23	3.63	4.85E-53	6.88E-51
IGHG4	1.23	-2.03	5.32E-07	1.84E-06
ANKRD33B	1.23	3.53	2.09E-43	1.49E-41
GPA33	1.23	0.15	1.83E-25	3.59E-24
FMO1	1.22	-1.08	4.43E-21	6.15E-20
IGLL5	1.22	-0.52	8.19E-10	4.01E-09
PODXL2	1.22	0.39	3.25E-53	4.70E-51
CCL3	1.22	-1.80	9.86E-14	7.12E-13
CEMP1	1.22	-0.79	1.01E-08	4.36E-08
C6orf223	1.21	-2.42	4.99E-34	1.82E-32
GLI2	1.21	0.22	1.03E-37	4.95E-36
HTR2A	1.21	-2.20	2.23E-20	2.91E-19
LTBP4	1.21	5.99	8.53E-33	2.78E-31
IDO1	1.21	-0.42	1.66E-14	1.30E-13
SYTL2	1.21	3.11	4.86E-37	2.20E-35
LCK	1.20	-0.66	7.95E-31	2.27E-29
TCEAL5	1.20	-2.07	3.58E-25	6.81E-24
SFRP1	1.20	5.46	8.08E-30	2.17E-28
HSH2D	1.20	-0.83	4.73E-38	2.39E-36
OLFML1	1.20	2.74	1.99E-61	4.69E-59
GZMK	1.20	-1.00	2.25E-21	3.22E-20
BCL11B	1.20	-1.92	2.79E-23	4.61E-22
KRT16P6	1.20	-2.35	6.03E-18	6.42E-17
ADAMTSL2	1.20	2.00	6.13E-25	1.15E-23
GNA14	1.20	0.77	2.25E-42	1.52E-40
HBG2	1.20	-1.63	2.55E-17	2.58E-16
ATP1B2	1.20	1.99	2.23E-62	6.03E-60
DDX3Y	1.19	1.94	0.005904222	0.010918052
CARNS1	1.19	3.32	6.98E-45	5.49E-43
FCRL6	1.19	-2.24	3.77E-23	6.18E-22
HMCN2	1.19	3.04	3.03E-28	7.28E-27
ITGB7	1.18	-1.29	3.11E-33	1.05E-31
CCDC168	1.18	0.03	7.88E-27	1.69E-25
FCN1	1.18	1.61	7.74E-25	1.44E-23
USP9Y	1.18	2.09	0.003259527	0.006356628
IGKV3-20	1.18	-0.71	5.13E-09	2.30E-08
PDIA2	1.18	0.10	5.03E-23	8.17E-22
LGALS2	1.18	-1.82	1.37E-23	2.31E-22
KCNIP1	1.17	-1.41	1.98E-30	5.53E-29
IGKV1-5	1.17	-1.41	6.40E-09	2.84E-08
PDCL3P4	1.17	-0.50	1.15E-26	2.42E-25
KAZALD1	1.17	2.34	7.91E-51	9.74E-49
TP63	1.16	-0.47	3.30E-32	1.03E-30
SLITRK4	1.16	1.51	1.83E-20	2.42E-19
CREB5	1.16	4.37	2.81E-45	2.28E-43
IGHA2	1.16	-0.35	9.36E-08	3.58E-07
BRSK1	1.16	1.17	2.90E-45	2.33E-43
COLQ	1.16	3.71	1.58E-25	3.12E-24

GUCA1C	1.16	2.30	1.35E-15	1.16E-14
PRRX2	1.16	-1.74	2.56E-14	1.96E-13
GATM	1.16	4.78	9.51E-30	2.54E-28
MEF2B	1.15	-1.65	2.97E-16	2.71E-15
SNCAIP	1.15	0.89	2.63E-47	2.46E-45
SERTAD4	1.15	1.85	4.43E-24	7.75E-23
TM6SF2	1.15	-1.36	1.27E-41	7.94E-40
FSCN1	1.15	2.55	1.12E-25	2.23E-24
PTP4A2P2	1.15	-2.62	8.30E-26	1.67E-24
PDE4C	1.15	2.30	5.92E-42	3.82E-40
RPS12P21	1.15	-1.83	4.07E-10	2.06E-09
SFRP2	1.15	-0.85	5.86E-09	2.61E-08
C1QTNF3	1.15	0.86	1.68E-39	9.21E-38
KCNJ4	1.14	3.74	1.68E-24	3.05E-23
IER3	1.14	4.20	2.09E-25	4.06E-24
EGR1	1.14	4.63	1.39E-17	1.45E-16
IGHV3-7	1.14	-2.51	5.03E-10	2.51E-09
CCDC3	1.14	3.63	5.06E-50	5.94E-48
TBC1D10C	1.13	-0.12	2.07E-28	5.02E-27
PDE5A	1.13	4.32	2.39E-38	1.22E-36
C11orf80	1.13	1.14	1.59E-52	2.18E-50
ZMYND12	1.13	0.92	4.99E-49	5.46E-47
RMI2	1.13	-0.36	1.98E-25	3.86E-24
GLYATL2	1.13	-2.03	2.30E-29	5.95E-28
IGLC3	1.13	0.52	4.47E-08	1.79E-07
ADAM19	1.12	4.35	1.14E-28	2.80E-27
NTNG2	1.12	0.45	4.63E-35	1.81E-33
TIGIT	1.12	-2.06	2.46E-21	3.51E-20
COL1A2	1.12	7.84	3.22E-27	7.17E-26
SYT17	1.12	0.16	2.18E-48	2.32E-46
BRINP1	1.12	-0.98	5.37E-20	6.81E-19
MMP16	1.12	1.16	2.01E-26	4.20E-25
RGS11	1.12	3.31	2.97E-40	1.73E-38
OR7E47P	1.12	-1.30	6.37E-19	7.36E-18
PODN	1.12	4.59	1.12E-41	7.06E-40
CD244	1.11	-2.12	3.63E-22	5.54E-21
IGHG3	1.11	0.26	5.27E-08	2.09E-07
RPL3P4	1.11	0.60	0.001758614	0.00361167
C12orf75	1.11	-0.17	1.99E-24	3.57E-23
BGN	1.11	6.26	1.06E-26	2.24E-25
JAML	1.11	2.19	3.83E-31	1.11E-29
SEMA5B	1.10	2.51	1.22E-46	1.08E-44
BOC	1.10	2.75	7.16E-45	5.60E-43
CLDN5	1.10	3.31	6.19E-15	5.04E-14
MXRA8	1.10	4.33	4.61E-30	1.26E-28
ALOX15	1.10	-2.63	3.17E-16	2.89E-15
TSPAN32	1.10	2.66	1.66E-31	4.92E-30
CD48	1.10	0.32	2.22E-25	4.28E-24
CTD-2510F5.6	1.09	-2.30	4.88E-07	1.70E-06
SELL	1.09	1.24	2.91E-25	5.58E-24
NELL2	1.09	-2.25	5.89E-20	7.45E-19
IGLV2-11	1.09	-2.39	2.37E-09	1.10E-08
IGSF10	1.09	3.07	5.59E-24	9.70E-23
HTRA1	1.08	5.94	7.30E-74	3.50E-71
ATP1A4	1.08	-0.82	3.58E-27	7.92E-26
KIT	1.08	1.62	1.46E-23	2.46E-22
SMPD3	1.08	0.54	1.08E-30	3.07E-29
TLR7	1.08	-0.12	2.66E-22	4.10E-21
CERCAM	1.08	3.28	3.80E-30	1.04E-28
RP3-395C13.1	1.08	-2.48	3.07E-26	6.31E-25
KCNA5	1.08	1.58	2.98E-42	1.98E-40
LOXL1	1.08	3.74	1.22E-23	2.08E-22
FBLN1	1.07	6.27	1.25E-36	5.50E-35
FZD2	1.07	-0.32	5.78E-21	7.93E-20
RARRES2	1.07	2.19	1.95E-15	1.66E-14
MNS1	1.07	0.72	1.32E-38	6.88E-37
ODF3B	1.07	1.58	1.67E-17	1.72E-16
LUZP2	1.07	-2.47	2.34E-18	2.57E-17
DPYSL3	1.07	5.61	5.87E-50	6.78E-48
GNB3	1.07	2.73	2.68E-42	1.79E-40
PTGDR2	1.07	1.85	1.60E-31	4.77E-30
RPL41P2	1.07	1.35	1.32E-09	6.31E-09
TFF3	1.07	-0.34	4.34E-14	3.25E-13
MRAP2	1.07	-0.65	1.50E-24	2.73E-23
SEZ6L2	1.06	-1.18	2.41E-20	3.15E-19
CD8B	1.06	-2.32	8.66E-18	9.15E-17
DPEP2	1.06	0.87	2.45E-31	7.17E-30
ST8SIA2	1.06	1.95	3.16E-29	8.09E-28
HMGN2	1.06	6.57	7.54E-60	1.68E-57
KLHDC9	1.06	1.36	8.61E-40	4.84E-38
GPAA1P1	1.06	-2.75	8.35E-14	6.08E-13
SLC22A7	1.06	-0.69	6.90E-14	5.06E-13
DTX4	1.06	1.96	7.81E-46	6.54E-44

NPR3	1.06	5.91	3.42E-32	1.06E-30
EMILIN3	1.06	2.29	2.30E-26	4.76E-25
WFIKKN2	1.06	-1.74	3.22E-25	6.15E-24
SNCA	1.06	2.70	1.54E-34	5.82E-33
CHRNE	1.05	-0.75	1.58E-16	1.49E-15
IGHEP2	1.05	-2.46	1.01E-15	8.80E-15
CD96	1.05	-0.76	9.88E-20	1.23E-18
IGLV3-21	1.05	-2.35	3.79E-08	1.53E-07
IGKV3-11	1.05	-1.79	3.38E-07	1.20E-06
CXCR1	1.05	-0.66	2.35E-13	1.63E-12
RAB7B	1.05	1.13	8.58E-37	3.80E-35
DACT1	1.05	1.54	1.53E-27	3.48E-26
VAT1L	1.05	3.81	3.26E-21	4.60E-20
GALNT5	1.04	-1.12	5.56E-16	4.94E-15
CYP4F3	1.04	-0.89	5.83E-27	1.26E-25
SRCIN1	1.04	0.00	2.14E-30	5.98E-29
TNNT1	1.04	6.28	2.42E-23	4.02E-22
HSD17B14	1.04	0.11	4.65E-26	9.45E-25
SNAP47	1.04	4.75	4.04E-55	6.83E-53
GLI1	1.04	-0.41	4.45E-29	1.12E-27
P2RY13	1.04	0.55	7.48E-28	1.74E-26
P2RX1	1.04	-0.07	1.24E-23	2.10E-22
NXPH4	1.04	-0.27	4.05E-19	4.75E-18
CETP	1.04	-1.45	4.30E-16	3.86E-15
C17orf107	1.03	-1.04	1.12E-23	1.90E-22
RP11-693N9.2	1.03	1.35	2.75E-47	2.56E-45
KLRK1	1.03	0.57	5.09E-29	1.28E-27
IGLV2-14	1.03	-1.40	1.15E-07	4.36E-07
HPGDS	1.03	0.04	7.87E-23	1.26E-21
MDFI	1.03	-1.15	1.73E-10	9.11E-10
GLT8D2	1.03	3.27	7.68E-56	1.40E-53
TAGLN3	1.03	-2.66	2.86E-21	4.05E-20
LFNG	1.03	1.71	7.70E-23	1.24E-21
P3H3	1.03	3.76	2.97E-34	1.10E-32
HLA-DPB1	1.03	5.12	3.50E-39	1.85E-37
BACH2	1.03	1.75	2.77E-53	4.03E-51
SOD3	1.02	4.04	1.52E-21	2.20E-20
PLPPR4	1.02	-0.44	5.16E-16	4.59E-15
PRR5L	1.02	0.52	5.85E-30	1.58E-28
COLEC11	1.02	2.07	4.18E-29	1.06E-27
RLN2	1.02	-2.23	1.07E-21	1.57E-20
C14orf132	1.02	2.81	3.81E-21	5.35E-20
ANTXR1	1.02	5.45	1.61E-41	1.00E-39
ESPNL	1.02	-1.92	2.41E-10	1.25E-09
RP11-420K14.2	1.02	-2.46	3.38E-10	1.72E-09
CPZ	1.02	-1.48	2.81E-14	2.14E-13
FRMD7	1.02	-2.05	3.20E-10	1.63E-09
ARHGAP33	1.01	1.56	9.54E-34	3.41E-32
SCAMP5	1.01	2.06	2.10E-32	6.66E-31
C9orf24	1.01	1.59	2.71E-30	7.48E-29
MSTN	1.01	-1.71	1.16E-13	8.32E-13
CCL8	1.01	-1.01	6.95E-13	4.64E-12
ARID5A	1.01	4.15	5.44E-25	1.03E-23
FGF17	1.01	-2.53	9.75E-22	1.43E-20
IGKV4-1	1.00	-0.84	4.86E-08	1.93E-07
TRAF3IP3	1.00	0.59	1.64E-31	4.88E-30
IGLV1-40	1.00	-2.32	1.70E-07	6.30E-07
LIPH	1.00	-0.35	4.18E-33	1.40E-31
CTF1	1.00	2.98	1.98E-47	1.90E-45
TNNI3	1.00	11.46	7.37E-20	9.27E-19
CYP4F24P	1.00	-2.70	3.27E-07	1.17E-06
PHF24	0.99	-1.64	3.74E-24	6.57E-23
BEGAIN	0.99	-0.43	2.08E-29	5.40E-28
CD5	0.99	-1.80	2.30E-19	2.75E-18
LAMP3	0.99	-2.30	1.18E-15	1.02E-14
CCL21	0.99	2.43	1.85E-11	1.07E-10
KCNMB2	0.99	1.26	5.09E-24	8.88E-23
TMEM221	0.99	-2.42	1.12E-21	1.63E-20
SLC44A5	0.99	0.68	1.29E-09	6.18E-09
TSPAN11	0.99	1.40	1.48E-29	3.88E-28
HTR2B	0.99	0.09	2.86E-16	2.62E-15
NHLRC4	0.99	-1.57	3.23E-18	3.52E-17
PLCE1	0.99	5.13	7.11E-37	3.18E-35
WBSCR27	0.99	-2.27	3.47E-14	2.62E-13
POSTN	0.99	6.13	3.45E-11	1.95E-10
HSD11B2	0.98	-1.55	8.60E-18	9.09E-17
MMP9	0.98	-0.69	9.11E-09	3.95E-08
IDSP1	0.98	-2.76	4.43E-13	3.01E-12
GPC6	0.98	1.98	8.36E-22	1.23E-20
NTF3	0.98	-0.46	1.89E-38	9.72E-37
BTN3A1	0.98	3.66	1.56E-38	8.11E-37
RP11-153M3.1	0.98	-2.36	1.95E-20	2.56E-19
KIF1A	0.98	0.19	2.79E-14	2.13E-13

CNTNAP3P1	0.98	-1.97	2.82E-17	2.84E-16
POU6F2	0.98	-1.26	6.40E-19	7.39E-18
IGHV3-23	0.98	-1.86	4.13E-07	1.45E-06
CTC-575D19.1	0.98	-0.29	0.000846629	0.001831939
RP11-113C12.4	0.97	-2.53	3.27E-18	3.56E-17
CHST6	0.97	0.72	1.81E-29	4.71E-28
C11orf65	0.97	-0.85	9.04E-38	4.40E-36
LRRN4CL	0.97	1.63	6.82E-46	5.74E-44
LMX1B	0.97	-0.57	3.06E-34	1.13E-32
AL589743.1	0.97	-0.28	5.15E-18	5.50E-17
ISG15	0.97	1.75	1.24E-10	6.65E-10
QPCT	0.97	1.48	5.31E-27	1.15E-25
CSPG5	0.97	-2.04	1.04E-24	1.91E-23
BCL6B	0.97	3.93	1.16E-35	4.71E-34
GJC2	0.97	-0.74	8.81E-17	8.47E-16
LPAR2	0.97	-0.40	1.18E-18	1.34E-17
ARSI	0.97	-2.07	9.14E-14	6.63E-13
B3GALT5	0.97	-1.13	3.40E-19	4.02E-18
REM1	0.96	1.09	3.86E-33	1.29E-31
GRM2	0.96	-1.29	9.01E-21	1.22E-19
CLU	0.96	6.83	4.65E-40	2.68E-38
SAP25	0.96	-0.49	1.15E-12	7.54E-12
CILP	0.96	4.96	4.69E-16	4.20E-15
COL15A1	0.96	6.38	1.60E-40	9.47E-39
NUPR2	0.96	-1.55	2.57E-20	3.35E-19
FAM216B	0.96	-1.09	6.73E-14	4.94E-13
IFI6	0.96	3.69	4.23E-23	6.90E-22
RSAD2	0.96	2.80	1.84E-28	4.47E-27
P2RY8	0.96	2.31	3.19E-35	1.26E-33
THEMIS	0.96	-2.60	6.36E-12	3.86E-11
CCER2	0.96	-2.47	6.52E-14	4.80E-13
IGLV1-44	0.96	-2.22	4.14E-07	1.45E-06
ZNF541	0.96	0.54	2.97E-23	4.91E-22
RTN4R	0.96	-2.23	2.36E-11	1.35E-10
PDZD4	0.95	1.79	4.87E-27	1.06E-25
CD74	0.95	7.52	5.75E-34	2.08E-32
STAB2	0.95	-1.68	2.63E-16	2.42E-15
SALL3	0.95	-2.52	3.35E-09	1.53E-08
PGPEP1L	0.95	-0.07	1.45E-22	2.28E-21
LRRC10	0.95	4.78	3.99E-23	6.53E-22
ANGPTL7	0.95	0.08	1.36E-12	8.89E-12
CD6	0.95	-0.44	2.38E-30	6.60E-29
DOK5	0.94	2.95	1.18E-28	2.88E-27
GADD45G	0.94	2.90	9.18E-10	4.46E-09
IL17D	0.94	2.01	5.92E-42	3.82E-40
EIF4HP2	0.94	-0.66	4.62E-30	1.26E-28
TSPAN9	0.94	7.15	9.89E-65	3.13E-62
GDF15	0.94	-1.88	4.25E-16	3.82E-15
REEP2	0.94	1.77	3.10E-23	5.12E-22
BMP4	0.94	2.70	1.58E-40	9.39E-39
CD163L1	0.94	1.45	7.51E-21	1.02E-19
PTPRT	0.94	-1.21	5.37E-15	4.40E-14
ANKDD1B	0.94	-1.77	2.33E-20	3.05E-19
SSXP10	0.94	-1.55	1.50E-09	7.12E-09
MX1	0.94	4.16	8.09E-44	6.08E-42
COL12A1	0.94	5.48	1.59E-24	2.88E-23
AGRN	0.94	3.50	7.89E-23	1.26E-21
COL6A4P2	0.94	-2.22	4.82E-16	4.31E-15
SOX15	0.93	-0.38	4.28E-27	9.41E-26
STAC	0.93	-0.85	3.06E-29	7.84E-28
ROR2	0.93	-0.47	4.01E-25	7.61E-24
UTY	0.93	0.62	0.010662731	0.018641138
C1orf204	0.93	1.75	1.12E-32	3.64E-31
UBA7	0.93	4.19	1.73E-57	3.49E-55
CX3CL1	0.93	4.46	5.55E-37	2.49E-35
IGKV2-28	0.93	-2.27	1.97E-06	6.33E-06
ALDH1A2	0.93	3.38	1.07E-37	5.11E-36
ARHGAP22	0.93	1.73	7.35E-38	3.63E-36
SLC6A4	0.93	-0.87	1.86E-18	2.06E-17
SEPT1	0.93	0.24	3.26E-25	6.20E-24
FBXL16	0.93	-2.51	1.56E-19	1.90E-18
RP11-54D18.2	0.93	-2.14	6.50E-13	4.36E-12
TSKS	0.93	-1.33	2.04E-18	2.26E-17
MAFK	0.93	5.53	8.76E-27	1.87E-25
DBP	0.92	1.44	2.54E-23	4.22E-22
PAPLN	0.92	2.33	6.89E-31	1.98E-29
SLCO4C1	0.92	-1.46	8.32E-16	7.28E-15
ACTA1	0.92	11.14	5.34E-13	3.60E-12
TMEM71	0.92	5.17	3.90E-37	1.79E-35
PNMAL2	0.92	0.28	1.03E-24	1.89E-23
RTP4	0.92	0.60	1.19E-35	4.84E-34
CXCR2	0.92	-0.16	7.78E-13	5.18E-12
CST7	0.92	-0.75	2.50E-16	2.31E-15

HYAL4	0.92	-1.85	3.60E-20	4.61E-19
EMID1	0.92	-1.29	1.22E-15	1.06E-14
PRKCQ	0.92	-1.73	1.14E-17	1.20E-16
CYP2J2	0.92	6.70	2.50E-45	2.04E-43
PTCH2	0.92	0.09	2.71E-21	3.85E-20
PPP2R2B	0.92	0.69	1.70E-32	5.44E-31
BGLAP	0.92	-1.80	6.06E-10	3.00E-09
DGKI	0.92	0.75	5.51E-21	7.58E-20
VTCN1	0.92	-1.24	4.95E-22	7.42E-21
EXT1	0.91	4.46	7.15E-67	2.40E-64
KLRF1	0.91	-1.77	4.36E-18	4.68E-17
GVQW2	0.91	-1.60	3.54E-26	7.24E-25
KIRREL3	0.91	-0.31	1.19E-30	3.36E-29
SKP1P1	0.91	-2.47	1.18E-21	1.72E-20
LIMD2	0.91	1.32	7.75E-17	7.48E-16
IGLV1-47	0.91	-2.61	2.95E-07	1.06E-06
IFIT2	0.90	3.09	9.74E-33	3.17E-31
MAGED4B	0.90	-0.38	1.94E-16	1.81E-15
LTBP3	0.90	5.55	8.54E-44	6.39E-42
COLGALT2	0.90	3.63	2.34E-51	2.97E-49
TNNT3	0.90	5.42	2.24E-23	3.74E-22
KIF21B	0.90	0.31	6.93E-32	2.11E-30
CCDC113	0.90	2.48	1.61E-51	2.07E-49
RASGRP1	0.90	-0.19	5.20E-17	5.12E-16
THEM5	0.90	-2.04	8.16E-23	1.30E-21
IGLV3-1	0.90	-2.36	1.73E-06	5.60E-06
MYCN	0.90	-1.65	5.87E-15	4.78E-14
IGSF1	0.90	2.39	1.88E-20	2.47E-19
FLRT2	0.90	2.38	2.83E-29	7.28E-28
GPRASP1	0.90	4.40	1.19E-33	4.22E-32
CD7	0.90	-2.60	1.65E-10	8.70E-10
IGKV1D-39	0.90	-1.58	3.53E-06	1.10E-05
KCNS3	0.90	1.95	1.27E-39	7.04E-38
AC004754.3	0.90	-0.11	4.24E-21	5.90E-20
HLA-DOA	0.89	1.89	3.19E-26	6.56E-25
PTPRH	0.89	0.59	2.45E-14	1.88E-13
SLC30A2	0.89	2.75	4.53E-19	5.28E-18
OMD	0.89	3.53	7.19E-17	6.99E-16
ZNF536	0.89	1.28	6.43E-30	1.73E-28
CPA3	0.89	1.37	7.71E-19	8.84E-18
APOBEC3A	0.89	-0.71	2.33E-14	1.80E-13
VSX1	0.89	-1.66	5.88E-23	9.51E-22
COL24A1	0.89	1.00	2.21E-14	1.71E-13
TCEAL2	0.89	-1.04	5.01E-17	4.94E-16
TNFRSF12A	0.89	4.83	1.21E-14	9.64E-14
MYADML2	0.89	1.33	4.96E-16	4.42E-15
CD8A	0.89	-0.39	1.92E-15	1.63E-14
TNXB	0.89	6.35	3.48E-22	5.31E-21
IFIT3	0.88	3.51	1.29E-27	2.94E-26
RSPO3	0.88	-1.11	4.26E-13	2.90E-12
MAPK10	0.88	1.77	3.49E-29	8.88E-28
CD300LF	0.88	-1.49	1.03E-14	8.23E-14
C3orf80	0.88	-2.42	1.55E-12	1.01E-11
CCDC74A	0.88	-0.20	3.76E-28	8.93E-27
PEX11G	0.88	-0.94	3.14E-18	3.43E-17
IGLV2-23	0.88	-2.21	3.94E-06	1.22E-05
DDAH2	0.88	4.36	2.16E-26	4.50E-25
CCDC74B	0.88	-1.17	1.63E-23	2.75E-22
LRRTM1	0.87	-2.39	1.14E-15	9.91E-15
IQCA1	0.87	-1.66	1.52E-17	1.57E-16
PYHIN1	0.87	-1.86	3.09E-15	2.58E-14
EPHX2	0.87	4.79	8.46E-50	9.71E-48
CH507-9B2.1	0.87	0.50	1.04E-06	3.47E-06
RUNDC3A	0.87	-1.07	2.41E-27	5.42E-26
HLA-DPA1	0.87	5.72	9.21E-35	3.54E-33
TSPAN8	0.87	-1.06	1.07E-11	6.37E-11
PCOLCE	0.87	4.33	3.72E-18	4.02E-17
IGLV3-19	0.87	-2.36	2.58E-06	8.17E-06
APLP1	0.87	2.58	2.96E-33	1.01E-31
TNNC2	0.87	-1.99	1.64E-14	1.29E-13
CACNA1G	0.87	-1.39	2.81E-20	3.64E-19
BDNF	0.87	3.13	4.27E-14	3.21E-13
EPHB3	0.87	0.40	1.07E-18	1.21E-17
CRIP1	0.87	3.76	1.78E-11	1.03E-10
CDH23	0.87	2.65	7.59E-28	1.77E-26
PCED1B	0.87	0.35	1.42E-34	5.41E-33
ZNF831	0.87	-1.98	6.27E-13	4.21E-12
SCARF2	0.86	1.12	6.58E-14	4.85E-13
ACHE	0.86	-0.81	5.46E-20	6.92E-19
ADAMTS7P4	0.86	-1.78	2.40E-19	2.87E-18
PHOSPHO1	0.86	-0.96	2.50E-27	5.59E-26
CRTAM	0.86	-2.47	1.31E-15	1.13E-14
PGAM1P5	0.86	-0.66	1.45E-29	3.80E-28

PAQR6	0.86	-0.04	8.60E-31	2.45E-29
DNAJC22	0.86	-1.29	3.75E-21	5.26E-20
SSUH2	0.86	1.22	5.32E-37	2.39E-35
ATP6V1G2	0.86	0.73	2.49E-26	5.16E-25
RELN	0.86	1.46	1.51E-11	8.81E-11
RP11-777B9.5	0.86	-1.71	0.00010588	0.00026633
CHODL	0.86	-1.27	4.43E-19	5.17E-18
NTRK1	0.86	-0.13	1.93E-14	1.50E-13
EOMES	0.86	-2.14	5.51E-14	4.10E-13
TECRP1	0.85	-0.75	0.002205262	0.004451988
SPON2	0.85	3.01	4.01E-16	3.61E-15
LAMB4	0.85	-1.68	3.53E-14	2.67E-13
GDPD3	0.85	1.28	4.02E-29	1.02E-27
RAB26	0.85	-0.66	7.94E-19	9.10E-18
TNF	0.85	-1.25	9.19E-13	6.10E-12
GAL3ST4	0.85	0.80	1.83E-33	6.39E-32
NREP	0.85	4.95	3.59E-29	9.11E-28
STAMBPL1	0.85	1.97	1.94E-39	1.06E-37
PARP10	0.85	2.73	1.88E-18	2.09E-17
LDHAP4	0.85	-1.87	2.08E-12	1.33E-11
NTN5	0.85	-2.43	8.48E-16	7.42E-15
MS4A2	0.85	0.39	3.84E-17	3.83E-16
STMN3	0.85	2.30	3.21E-22	4.91E-21
HSPA2	0.85	4.41	5.16E-23	8.38E-22
DEFB124	0.85	-2.26	4.08E-17	4.06E-16
HHAT	0.85	1.49	8.77E-44	6.53E-42
FGF14	0.85	2.87	7.72E-38	3.80E-36
RAB37	0.85	1.08	2.21E-46	1.91E-44
TCEAL7	0.85	1.61	3.37E-32	1.05E-30
HLA-DMB	0.85	3.40	2.17E-27	4.89E-26
PMP2	0.85	-0.23	3.10E-13	2.13E-12
TMEM54	0.85	1.65	9.15E-16	7.98E-15
XAF1	0.85	5.62	1.04E-39	5.81E-38
COL21A1	0.84	5.77	5.21E-27	1.13E-25
DUOXA1	0.84	-1.32	6.36E-21	8.71E-20
USP51	0.84	1.25	5.68E-53	7.93E-51
OAS1	0.84	2.57	5.05E-28	1.19E-26
MED12L	0.84	-0.03	4.62E-15	3.81E-14
BEND5	0.84	1.18	2.39E-48	2.54E-46
CHAD	0.84	-1.81	1.31E-13	9.32E-13
MTCO3P22	0.84	-1.12	0.000118476	0.000295793
MID1	0.84	3.36	7.79E-29	1.93E-27
ESR1	0.84	1.54	7.75E-20	9.74E-19
GPR27	0.84	1.97	6.10E-27	1.32E-25
CAPN5	0.84	1.79	9.72E-38	4.70E-36
EDA2R	0.84	1.60	1.16E-15	1.01E-14
ANGPTL5	0.84	0.68	2.20E-12	1.40E-11
GBP5	0.84	0.68	2.60E-15	2.19E-14
TMEM35A	0.84	-1.97	9.32E-17	8.94E-16
COL3A1	0.84	7.88	2.16E-12	1.38E-11
ANKRD1	0.83	11.89	1.74E-18	1.94E-17
CTD-2587H24.4	0.83	-0.79	1.47E-07	5.49E-07
HSPB1P1	0.83	-2.03	7.51E-10	3.69E-09
IGHV3-33	0.83	-2.29	4.64E-05	0.000123225
THBS2	0.83	4.88	3.76E-19	4.42E-18
NLGN3	0.83	-0.44	4.46E-34	1.63E-32
SERPINI1	0.83	2.99	2.54E-37	1.18E-35
LINC00672	0.83	1.05	3.73E-33	1.26E-31
NRIP2	0.83	2.32	1.52E-47	1.50E-45
RAB33A	0.83	-0.52	2.18E-16	2.02E-15
STX1B	0.83	0.05	6.48E-33	2.15E-31
PLXDC1	0.83	4.96	7.73E-47	6.92E-45
OLR1	0.83	-0.98	1.63E-13	1.15E-12
TM7SF2	0.83	4.63	7.26E-19	8.35E-18
ASIC1	0.83	-0.28	1.91E-23	3.20E-22
NCR1	0.83	-1.57	8.58E-16	7.50E-15
ASB18	0.83	2.11	6.37E-21	8.71E-20
RP11-466H18.1	0.83	0.01	0.022740787	0.037103856
AC110615.1	0.83	0.96	3.61E-09	1.65E-08
PIK3IP1	0.83	4.49	2.21E-31	6.49E-30
CTLA4	0.83	-1.39	2.59E-15	2.17E-14
CH25H	0.83	-0.91	9.47E-07	3.17E-06
DEFA1	0.82	-1.99	4.50E-05	0.000119889
ADAM8	0.82	0.23	1.14E-14	9.06E-14
ARVCF	0.82	3.61	1.87E-20	2.47E-19
RP11-588F10.1	0.82	0.36	1.17E-10	6.27E-10
AGER	0.82	1.41	4.16E-22	6.28E-21
RNF165	0.82	1.30	9.96E-28	2.31E-26
HESX1	0.82	-1.03	2.43E-24	4.33E-23
DENND6B	0.82	0.67	4.24E-22	6.39E-21
MATK	0.82	-1.38	1.76E-16	1.65E-15
EPHA3	0.82	3.53	1.90E-14	1.48E-13
ZBTB12	0.82	-0.29	6.33E-23	1.02E-21

CHIT1	0.82	-1.82	1.81E-08	7.62E-08
CTGF	0.82	6.53	3.50E-14	2.64E-13
PRRT1	0.82	-0.04	1.96E-16	1.82E-15
MIA	0.82	-1.50	2.60E-13	1.80E-12
ECT2L	0.82	1.37	3.01E-13	2.07E-12
ANKRD29	0.82	3.05	3.81E-32	1.18E-30
GDNF	0.82	-1.48	1.19E-20	1.59E-19
CETN4P	0.81	-2.05	6.68E-15	5.41E-14
SPACA9	0.81	2.06	1.41E-44	1.09E-42
GSTM1	0.81	0.91	0.024933016	0.040315476
ITGAX	0.81	1.94	4.85E-18	5.20E-17
DRP2	0.81	-2.06	1.19E-12	7.80E-12
AC092651.1	0.81	-2.34	0.000415711	0.000953597
FAM196A	0.81	-1.99	1.62E-20	2.15E-19
ANOS1	0.81	3.18	1.63E-22	2.55E-21
FGF18	0.81	2.79	1.28E-15	1.11E-14
CCDC190	0.81	-1.65	5.26E-12	3.21E-11
TNN	0.81	-2.06	2.04E-12	1.30E-11
DYRK1B	0.81	3.17	1.59E-31	4.73E-30
HPX	0.81	-1.30	1.40E-26	2.94E-25
EPHA5	0.81	-2.24	5.30E-10	2.64E-09
RP11-347C12.3	0.81	-0.42	1.12E-18	1.27E-17
C7orf50	0.81	4.37	3.16E-14	2.40E-13
TIMP2	0.80	7.04	1.10E-50	1.34E-48
TXLNGY	0.80	1.36	0.031332444	0.049628222
GPR68	0.80	0.58	1.11E-19	1.37E-18
MFAP2	0.80	0.78	6.75E-13	4.52E-12
ADAM22	0.80	2.01	7.76E-24	1.34E-22
BFSP1	0.80	-1.16	7.54E-15	6.10E-14
TMEM178A	0.80	1.22	1.92E-31	5.64E-30
COL8A1	0.80	5.37	1.37E-23	2.31E-22
STON2	0.80	1.40	2.36E-35	9.40E-34
PAMR1	0.80	3.47	1.13E-29	3.00E-28
CCR6	0.80	-2.45	1.62E-12	1.05E-11
ZGLP1	0.80	-0.81	9.06E-14	6.57E-13
RP11-79D8.2	0.80	-2.86	4.13E-10	2.08E-09
IL2RG	0.80	1.03	1.72E-22	2.69E-21
HLA-DQB1	0.79	3.92	4.78E-20	6.09E-19
CYTL1	0.79	-1.76	4.90E-13	3.32E-12
RP11-834C11.7	0.79	-1.76	2.15E-20	2.82E-19
SLIT3	0.79	4.27	1.60E-25	3.16E-24
NHLRC1	0.79	-0.89	7.25E-32	2.20E-30
RP11-213G2.1	0.79	-1.85	1.24E-14	9.84E-14
VMO1	0.79	-2.18	3.85E-07	1.36E-06
CAMK4	0.79	-1.18	2.93E-13	2.02E-12
FAM156B	0.79	3.30	7.97E-16	6.99E-15
RP11-248C1.3	0.79	-2.57	1.04E-07	3.96E-07
APBB3	0.79	3.56	6.42E-48	6.60E-46
GDF9	0.79	-1.18	2.80E-21	3.97E-20
EXTL1	0.79	1.81	2.95E-17	2.97E-16
PTK7	0.79	1.57	4.59E-27	1.01E-25
DSCAML1	0.79	-0.36	4.21E-19	4.93E-18
CD52	0.79	1.11	1.01E-13	7.27E-13
HYI	0.79	3.59	1.19E-33	4.22E-32
IGLV2-8	0.79	-2.59	1.04E-05	3.05E-05
TMEM163	0.79	-0.93	1.89E-15	1.61E-14
TNFRSF11B	0.79	2.61	3.91E-08	1.58E-07
LCNL1	0.79	2.01	1.01E-08	4.38E-08
CCDC154	0.79	-0.84	1.11E-13	7.96E-13
TRIM17	0.79	-1.62	3.75E-20	4.80E-19
PRRG3	0.79	1.40	1.75E-22	2.72E-21
GBAP1	0.79	0.57	6.71E-22	9.96E-21
ABC14-864958H18.2	0.79	-1.01	1.31E-17	1.37E-16
SLC25A45	0.79	2.43	1.13E-40	6.78E-39
LRRC36	0.78	-2.10	7.75E-09	3.40E-08
OR7E126P	0.78	-2.44	4.10E-15	3.40E-14
SCRG1	0.78	1.26	2.05E-19	2.46E-18
GPAA1P2	0.78	-0.01	1.55E-19	1.88E-18
COL5A1	0.78	5.06	2.47E-18	2.71E-17
S100B	0.78	1.43	7.99E-11	4.34E-10
LPAR4	0.78	-0.90	2.00E-11	1.16E-10
CTD-3088G3.8	0.78	0.55	2.68E-21	3.82E-20
TMC4	0.78	1.40	9.62E-26	1.92E-24
MMP25	0.78	0.74	3.98E-15	3.30E-14
FGF23	0.78	1.95	2.26E-06	7.22E-06
HERC5	0.78	2.43	1.99E-40	1.17E-38
SVEP1	0.78	4.74	1.28E-24	2.33E-23
MEGF6	0.78	1.59	1.62E-11	9.42E-11
SCML4	0.78	-1.70	2.27E-15	1.92E-14
ABCA4	0.78	-2.01	5.05E-18	5.40E-17
H2BFM	0.77	-2.23	5.81E-19	6.74E-18
JAK2	0.77	5.74	1.13E-13	8.10E-13

AL513523.1	0.77	0.93	0.000182206	0.00044116
AL513523.2	0.77	0.93	0.000182206	0.00044116
RCN3	0.77	2.81	1.42E-10	7.57E-10
ARHGEF4	0.77	0.87	6.81E-24	1.17E-22
SRRM4	0.77	1.23	2.45E-24	4.36E-23
TTC9	0.77	1.78	1.10E-26	2.33E-25
MKX	0.77	-2.02	2.39E-08	9.88E-08
CABLES1	0.77	1.80	1.84E-22	2.86E-21
TESPA1	0.77	0.08	1.21E-32	3.90E-31
TPPP3	0.77	2.65	3.63E-15	3.02E-14
TCEA2	0.77	3.63	2.64E-23	4.38E-22
TMEM200B	0.77	1.57	2.13E-25	4.13E-24
MST1R	0.77	0.30	2.84E-24	5.02E-23
SLC35F1	0.77	3.17	2.56E-18	2.81E-17
GPC3	0.77	2.36	3.36E-18	3.65E-17
CCDC85B	0.77	2.37	8.32E-06	2.47E-05
IL32	0.77	4.29	5.00E-19	5.81E-18
INMT	0.76	5.09	5.52E-23	8.93E-22
LSMEM2	0.76	3.69	4.19E-19	4.91E-18
XKR4	0.76	0.69	2.19E-15	1.84E-14
TTLL3	0.76	4.75	2.04E-45	1.68E-43
MYH10	0.76	5.76	2.13E-34	7.99E-33
DBIL5P	0.76	-1.97	7.84E-18	8.31E-17
CCNI2	0.76	0.17	1.72E-13	1.21E-12
FAM186A	0.76	-0.72	5.60E-21	7.70E-20
MN1	0.76	3.26	3.36E-32	1.05E-30
RPL7P3	0.76	1.33	4.90E-10	2.44E-09
CLDND2	0.76	-1.02	5.39E-11	2.98E-10
RP11-196G11.1	0.76	-2.80	8.71E-07	2.93E-06
CREG2	0.76	-1.15	2.86E-24	5.06E-23
CARMIL2	0.76	-1.99	4.16E-12	2.57E-11
EDIL3	0.76	4.35	4.92E-11	2.74E-10
GFI1	0.76	-2.07	4.56E-13	3.10E-12
PURG	0.76	-1.86	1.18E-17	1.24E-16
KRTCAP3	0.76	-1.81	9.61E-18	1.01E-16
PCOLCE2	0.76	6.18	3.54E-37	1.63E-35
NCF1	0.76	0.64	1.61E-15	1.37E-14
PTGFRN	0.76	6.60	1.67E-52	2.28E-50
LOXHD1	0.76	-0.37	1.98E-15	1.68E-14
TPSAB1	0.75	0.98	5.09E-08	2.02E-07
TRDC	0.75	-1.82	7.71E-08	2.99E-07
KLHL35	0.75	-1.07	2.72E-24	4.82E-23
MYBL1	0.75	2.47	5.00E-25	9.44E-24
PSPHP1	0.75	-1.96	0.009542009	0.016880931
DUOX1	0.75	1.05	1.28E-34	4.87E-33
SMO	0.75	2.57	2.30E-43	1.64E-41
RPS11P6	0.75	-1.55	6.41E-22	9.56E-21
ANKRD23	0.75	2.87	8.33E-13	5.54E-12
CELF6	0.75	1.51	3.41E-18	3.70E-17
B9D1	0.75	1.98	3.50E-36	1.50E-34
ADRA2A	0.75	-0.28	1.26E-09	6.03E-09
THBS3	0.75	3.55	5.03E-32	1.54E-30
LRRC75B	0.75	1.22	9.49E-23	1.51E-21
FAM150B	0.75	-0.34	4.60E-08	1.84E-07
CHAC1	0.75	0.04	1.68E-09	7.92E-09
TSTD1	0.75	1.81	3.14E-20	4.07E-19
ZMAT1	0.75	4.40	3.85E-19	4.52E-18
GARNL3	0.75	4.07	2.68E-36	1.16E-34
OAS3	0.75	3.52	4.17E-15	3.46E-14
IL23A	0.75	-2.41	2.82E-16	2.59E-15
CDRT4	0.74	1.33	1.48E-08	6.28E-08
SLC28A1	0.74	-0.13	3.43E-20	4.41E-19
GPR173	0.74	1.33	1.24E-23	2.10E-22
HLA-DRA	0.74	6.57	1.33E-22	2.10E-21
FBLN2	0.74	5.69	3.36E-17	3.37E-16
ASPRV1	0.74	-0.09	3.31E-30	9.08E-29
MBL1P	0.74	0.66	2.39E-30	6.63E-29
LYPD3	0.74	-2.24	1.28E-13	9.11E-13
KCNC4	0.74	3.28	4.07E-51	5.13E-49
C2orf27A	0.74	-0.99	6.47E-12	3.91E-11
TRIM45	0.74	3.58	8.96E-31	2.55E-29
SAMD3	0.74	-0.80	9.96E-21	1.34E-19
SFTPD	0.74	-0.81	3.59E-18	3.89E-17
PDE1A	0.74	4.00	1.90E-18	2.10E-17
NTRK2	0.74	3.33	5.88E-17	5.77E-16
KDM5D	0.74	2.52	0.027284614	0.043742084
AMT	0.74	4.50	1.41E-37	6.63E-36
NSUN5P2	0.74	2.63	2.89E-13	2.00E-12
ABHD11	0.74	3.35	2.04E-36	8.84E-35
RP11-134G8.6	0.74	-0.56	4.79E-23	7.81E-22
COL11A2	0.74	1.14	1.48E-16	1.40E-15
TCF21	0.74	2.87	5.93E-22	8.85E-21
RP11-466G12.2	0.74	-2.71	1.14E-11	6.73E-11

EVA1A	0.74	-1.12	1.63E-12	1.05E-11
AC240274.1	0.74	3.37	1.92E-25	3.75E-24
FAT3	0.74	-1.26	7.80E-09	3.42E-08
EVL	0.74	4.90	1.53E-47	1.50E-45
ROBO3	0.74	2.00	5.51E-24	9.56E-23
FAM181B	0.74	-1.52	9.61E-23	1.53E-21
HDAC10	0.74	1.89	1.41E-16	1.34E-15
FGF1	0.74	5.97	1.87E-38	9.68E-37
PCSK2	0.74	0.41	2.73E-15	2.29E-14
KANK4	0.74	-2.39	2.63E-06	8.31E-06
CHSY3	0.74	0.01	1.86E-13	1.30E-12
PTGFR	0.74	0.36	4.09E-07	1.44E-06
CD24	0.74	-0.87	2.34E-09	1.09E-08
C10orf105	0.73	-2.62	1.04E-07	3.95E-07
ADAM12	0.73	-0.29	2.47E-05	6.84E-05
LDOC1	0.73	0.80	2.29E-21	3.27E-20
ARHGAP1	0.73	5.95	1.22E-59	2.69E-57
C2orf16	0.73	-0.51	1.39E-25	2.76E-24
PTGES3P2	0.73	-2.01	3.57E-12	2.22E-11
PPP1R36	0.73	-1.69	8.91E-17	8.56E-16
ADGRA2	0.73	3.82	2.77E-16	2.55E-15
FZD1	0.73	4.50	2.95E-28	7.11E-27
RP11-234B24.6	0.73	1.50	0.00011552	0.000289146
ANO1	0.73	4.16	6.08E-36	2.54E-34
STX19	0.73	-2.56	6.40E-14	4.72E-13
PCDH19	0.73	0.53	1.59E-19	1.93E-18
SLC26A4	0.73	0.25	7.99E-16	7.01E-15
HFE2	0.73	5.42	1.37E-21	1.99E-20
CCNA1	0.73	-2.24	2.99E-09	1.38E-08
AMOT	0.73	2.72	5.53E-42	3.60E-40
RASGEF1C	0.73	-2.35	1.60E-11	9.31E-11
AMPH	0.73	1.77	3.08E-19	3.65E-18
NBL1	0.73	5.01	8.74E-18	9.22E-17
FBF1	0.73	1.63	4.26E-29	1.07E-27
TMEM130	0.73	-0.99	4.41E-12	2.72E-11
MTND4P12	0.73	2.90	0.000472819	0.001073424
SYT9	0.73	-1.90	7.80E-11	4.25E-10
IFT43	0.73	3.94	3.27E-40	1.89E-38
FAM231D	0.73	0.94	5.66E-29	1.41E-27
MAGED4	0.73	-1.31	2.02E-11	1.17E-10
BAIAP2L2	0.72	-1.63	2.80E-12	1.77E-11
C2orf70	0.72	-2.61	7.59E-11	4.14E-10
GDF7	0.72	1.70	3.87E-17	3.86E-16
APOC2	0.72	-2.19	2.16E-11	1.24E-10
STK32A	0.72	0.08	2.42E-21	3.46E-20
CGNL1	0.72	5.02	8.64E-25	1.60E-23
KCNMA1	0.72	1.71	6.12E-16	5.42E-15
HLA-J	0.72	-0.91	2.32E-13	1.61E-12
SCN11A	0.72	-1.75	2.87E-12	1.81E-11
C10orf35	0.72	0.98	1.95E-32	6.21E-31
C6orf25	0.72	-0.81	5.35E-24	9.29E-23
ROBO2	0.72	-2.10	1.16E-12	7.62E-12
GLUD1P7	0.72	-1.76	2.90E-15	2.43E-14
SESN3	0.72	3.90	3.69E-17	3.69E-16
CCND1	0.72	6.36	1.32E-46	1.16E-44
LRFN1	0.72	-2.31	1.08E-11	6.39E-11
ASTN1	0.72	0.03	4.96E-17	4.90E-16
GALNT8	0.72	0.73	3.72E-22	5.67E-21
ST6GALNAC5	0.72	-1.36	2.40E-13	1.67E-12
PSMB10	0.72	3.70	2.22E-12	1.42E-11
ZNF423	0.72	2.19	5.77E-30	1.56E-28
MARCKS	0.72	5.07	6.20E-18	6.60E-17
SLIT2	0.72	3.28	1.38E-24	2.52E-23
MBP	0.72	4.86	7.68E-33	2.52E-31
RP11-281O15.7	0.72	-1.19	5.12E-09	2.30E-08
EEF1A1P13	0.72	-1.63	1.35E-13	9.61E-13
ADGRB1	0.72	-1.44	5.65E-12	3.44E-11
LLNLF-187D8.1	0.72	-2.03	1.65E-09	7.80E-09
CXCR4	0.72	2.71	3.60E-14	2.71E-13
RAMP2	0.72	4.18	5.14E-28	1.21E-26
F2R	0.72	4.78	9.23E-12	5.52E-11
CD22	0.71	-1.41	1.61E-12	1.04E-11
BCL2A1	0.71	-0.92	4.12E-07	1.45E-06
PRRX1	0.71	6.01	3.99E-24	6.99E-23
RP11-96A1.4	0.71	2.42	2.21E-21	3.17E-20
ABHD1	0.71	1.31	3.22E-38	1.64E-36
ABI3BP	0.71	5.27	4.61E-15	3.80E-14
RHBDL1	0.71	-1.01	2.40E-09	1.12E-08
PRG4	0.71	2.04	9.06E-07	3.04E-06
INHBE	0.71	-1.82	1.82E-16	1.71E-15
CACNA2D2	0.71	1.45	6.00E-28	1.40E-26
CYSRT1	0.71	-2.49	1.84E-11	1.07E-10
ADAMTS13	0.71	1.59	6.04E-20	7.63E-19

MTMR9LP	0.71	3.10	1.44E-29	3.80E-28
ABHD18	0.71	4.82	2.16E-17	2.20E-16
GADD45A	0.71	5.03	5.86E-29	1.46E-27
MEI1	0.71	-1.18	6.01E-14	4.45E-13
RAB39B	0.71	-1.81	1.22E-12	8.00E-12
HSPB6	0.71	8.72	7.54E-16	6.63E-15
GIMAP1-GIMAP5	0.71	0.24	0.014101038	0.024054627
PLEKHB1	0.71	3.05	5.34E-28	1.25E-26
RERGL	0.71	1.41	3.85E-22	5.84E-21
HCAR2	0.71	-2.44	1.68E-05	4.79E-05
GPM6A	0.71	0.04	2.73E-12	1.72E-11
RP11-467J12.4	0.71	-0.38	4.02E-16	3.62E-15
NAT8L	0.71	-1.13	7.29E-09	3.21E-08
TREX2	0.71	-2.44	1.33E-09	6.33E-09
RUNX2	0.71	1.07	3.79E-17	3.79E-16
MSI1	0.71	1.06	1.05E-23	1.79E-22
SLC16A6	0.71	0.13	5.80E-12	3.53E-11
PTPRR	0.71	-0.60	1.02E-12	6.76E-12
GRIN2C	0.71	-0.27	2.77E-21	3.93E-20
MSH5-SAPCD1	0.70	1.77	1.44E-17	1.50E-16
TMSB4Y	0.70	-2.23	0.003790564	0.00730654
TRO	0.70	2.16	1.19E-28	2.91E-27
EVA1B	0.70	0.64	1.03E-05	3.00E-05
FCRL3	0.70	-1.78	1.96E-12	1.26E-11
MEOX1	0.70	4.11	3.15E-19	3.74E-18
CCDC54	0.70	-1.44	9.66E-10	4.68E-09
BAIAP3	0.70	1.01	3.88E-15	3.22E-14
AK8	0.70	-2.48	8.36E-14	6.09E-13
C1QTNF5	0.70	3.13	3.36E-16	3.04E-15
EDNRA	0.70	4.82	2.59E-17	2.62E-16
CPEB1	0.70	0.90	1.46E-23	2.46E-22
KCNA4	0.70	2.03	2.42E-17	2.45E-16
FXYD1	0.70	6.56	1.30E-15	1.12E-14
HLA-DRB3	0.70	3.11	0.003100623	0.006079296
CASP1	0.70	2.86	2.79E-35	1.11E-33
GRIA1	0.70	-0.57	2.91E-15	2.44E-14
OSBP2	0.70	3.05	5.79E-25	1.09E-23
ENPP2	0.70	2.69	1.00E-13	7.22E-13
MDH1B	0.70	0.51	1.90E-27	4.30E-26
SOCS2	0.70	4.47	1.96E-35	7.91E-34
IER2	0.70	4.20	6.79E-17	6.61E-16
IGLV1-51	0.70	-1.53	2.29E-06	7.31E-06
FAM90A1	0.70	-2.19	1.13E-13	8.09E-13
FAM102A	0.70	3.49	1.05E-38	5.50E-37
MYL2	0.70	13.10	4.16E-25	7.89E-24
PYROXD2	0.70	3.38	3.06E-31	8.92E-30
GRM1	0.70	2.48	2.82E-09	1.30E-08
C1orf54	0.70	2.79	1.97E-26	4.12E-25
PIEZO2	0.70	-1.04	7.19E-10	3.54E-09
MTURN	0.70	5.62	4.09E-33	1.37E-31
ACAN	0.70	-0.87	1.30E-09	6.19E-09
FMO4	0.70	2.60	1.90E-27	4.30E-26
C7orf61	0.70	-2.67	1.53E-09	7.23E-09
SLC5A7	0.69	-1.80	1.13E-06	3.75E-06
CD3G	0.69	-0.88	2.08E-11	1.20E-10
ITLN1	0.69	-0.26	0.002923002	0.005754618
HLA-DRB1	0.69	6.01	6.58E-16	5.80E-15
SLC6A9	0.69	-0.72	1.48E-12	9.62E-12
SPN	0.69	1.16	4.10E-22	6.19E-21
RAPSN	0.69	0.20	4.20E-10	2.12E-09
DEF6	0.69	0.70	5.84E-15	4.76E-14
NES	0.69	8.13	1.23E-29	3.25E-28
INHBA	0.69	1.82	2.37E-09	1.10E-08
GSDMB	0.69	2.90	5.05E-32	1.55E-30
CD72	0.69	-0.23	8.22E-09	3.59E-08
ERICH2	0.69	-0.64	1.84E-16	1.72E-15
SPATA18	0.69	1.25	4.72E-18	5.06E-17
CRIP3	0.69	3.12	1.16E-08	4.99E-08
OLFML3	0.69	4.06	1.19E-22	1.87E-21
SYNPO2L	0.69	8.25	2.44E-25	4.71E-24
SAMD9L	0.69	3.70	1.07E-14	8.51E-14
LINC01296	0.69	0.93	1.48E-13	1.05E-12
RP11-327P2.7	0.69	0.47	9.34E-19	1.06E-17
ANO9	0.69	-1.46	5.69E-11	3.14E-10
HSPB1	0.69	8.04	8.51E-07	2.87E-06
C1orf106	0.69	-1.30	6.73E-14	4.94E-13
GDPD2	0.69	-0.20	3.06E-20	3.96E-19
TNFRSF25	0.69	2.71	2.51E-25	4.83E-24
ZNF821	0.69	1.39	3.51E-36	1.50E-34
PLAC9	0.69	3.05	6.78E-09	3.00E-08
MAP6	0.69	2.57	1.29E-56	2.52E-54
BEX2	0.69	0.00	7.16E-12	4.32E-11
TMPRSS9	0.69	-1.11	1.48E-16	1.40E-15

GABRE	0.69	5.51	2.61E-20	3.38E-19
TSPY26P	0.68	1.64	8.13E-48	8.20E-46
HLA-DMA	0.68	4.14	2.82E-22	4.34E-21
PSD	0.68	-0.89	2.21E-10	1.15E-09
MTND1P23	0.68	6.69	0.001436217	0.00299958
TRIM74	0.68	-2.18	3.58E-06	1.12E-05
PRR7	0.68	-2.47	9.28E-07	3.11E-06
CCND2	0.68	7.40	2.94E-42	1.95E-40
VILL	0.68	1.62	4.60E-23	7.51E-22
ADAMTSL5	0.68	3.73	1.05E-28	2.57E-27
RIBC1	0.68	-0.92	1.57E-19	1.91E-18
AC002398.9	0.68	-1.31	0.00592403	0.010951054
C4orf47	0.68	-1.84	7.30E-14	5.33E-13
NRP2	0.68	3.31	1.05E-21	1.54E-20
SLC4A8	0.68	0.43	4.96E-15	4.08E-14
SLC16A2	0.68	3.59	2.33E-35	9.33E-34
PFN4	0.68	-0.88	4.61E-26	9.37E-25
CAPN14	0.68	-1.28	2.69E-12	1.70E-11
CIDEA	0.68	-0.43	0.000713909	0.001568259
LRRC53	0.68	-1.07	2.42E-10	1.25E-09
LRRN1	0.68	-2.52	4.53E-09	2.04E-08
RP4-800G7.2	0.68	1.90	4.82E-27	1.05E-25
IKZF3	0.68	1.04	3.68E-19	4.34E-18
F10	0.68	2.01	3.75E-17	3.75E-16
FAM188B	0.68	1.79	1.34E-33	4.73E-32
MANEAL	0.68	-0.02	5.47E-32	1.67E-30
ESRP1	0.68	-2.04	6.48E-07	2.22E-06
APOBEC3H	0.68	-2.67	2.13E-10	1.11E-09
ENC1	0.68	3.59	3.92E-17	3.90E-16
COPZ2	0.67	4.30	9.42E-29	2.32E-27
KLHL13	0.67	3.38	8.19E-21	1.12E-19
PAFAH2	0.67	3.86	9.96E-42	6.30E-40
S100A2	0.67	-0.55	2.40E-12	1.52E-11
KIF25	0.67	-1.80	6.73E-14	4.94E-13
CCDC191	0.67	1.35	2.80E-22	4.31E-21
HS3ST3A1	0.67	-1.76	5.43E-06	1.65E-05
DNM1	0.67	2.79	2.88E-22	4.42E-21
PER2	0.67	2.91	7.61E-16	6.70E-15
PKP3	0.67	-2.01	4.75E-09	2.14E-08
EFS	0.67	2.00	1.70E-27	3.86E-26
KCNH8	0.67	-1.94	6.72E-11	3.69E-10
INAFM1	0.67	1.70	3.70E-09	1.69E-08
RP11-656G20.1	0.67	-2.26	3.94E-09	1.79E-08
VTN	0.67	4.89	8.01E-13	5.33E-12
ZNF711	0.67	1.72	2.47E-08	1.02E-07
PAPPA2	0.67	-0.38	1.62E-11	9.42E-11
CCDC89	0.67	-1.51	9.04E-14	6.56E-13
LDLRAD2	0.67	2.23	2.32E-07	8.45E-07
ANKRD20A8P	0.67	-0.50	1.25E-15	1.08E-14
RP11-949J7.8	0.67	3.00	1.02E-22	1.62E-21
NDP	0.67	-1.20	2.59E-11	1.48E-10
NEB	0.67	3.39	2.07E-05	5.81E-05
FLG2	0.67	-1.45	7.80E-13	5.20E-12
USP11	0.67	5.00	8.71E-56	1.55E-53
SSPN	0.67	5.75	8.74E-26	1.75E-24
DHX58	0.67	2.01	2.02E-27	4.57E-26
AP3M2	0.66	4.20	8.90E-38	4.34E-36
WDR66	0.66	2.10	2.09E-42	1.41E-40
ZDHHC14	0.66	3.60	6.04E-36	2.53E-34
TMEM45B	0.66	-1.71	2.98E-14	2.27E-13
CYP27A1	0.66	3.34	9.08E-24	1.56E-22
TNFSF13B	0.66	1.07	2.16E-11	1.25E-10
NRGN	0.66	0.98	4.76E-10	2.38E-09
TANC2	0.66	3.11	4.98E-24	8.70E-23
RP11-182J1.14	0.66	-1.64	2.43E-08	1.01E-07
HLA-DQA1	0.66	4.83	3.95E-21	5.54E-20
B4GALNT4	0.66	-2.17	8.08E-08	3.13E-07
LOXL2	0.66	2.96	2.17E-14	1.68E-13
JCHAIN	0.66	1.20	0.00013178	0.000326237
ZC3H12B	0.66	0.25	1.59E-18	1.77E-17
F3	0.66	5.21	1.65E-16	1.55E-15
ENAM	0.66	3.02	9.74E-14	7.04E-13
MPO	0.66	-2.23	2.73E-09	1.26E-08
RP11-231P20.2	0.66	0.91	7.34E-21	1.00E-19
SLC26A11	0.66	1.82	3.53E-34	1.29E-32
CTA-963H5.5	0.66	-1.67	4.07E-12	2.51E-11
FOXD3	0.66	-2.66	3.99E-08	1.61E-07
IPCEF1	0.66	0.09	1.20E-16	1.15E-15
MMP2	0.66	6.90	1.63E-23	2.74E-22
ACBD4	0.66	2.37	2.26E-28	5.46E-27
NDC80	0.66	0.35	1.97E-17	2.01E-16
LRRC43	0.66	-2.13	2.99E-13	2.07E-12
GPR61	0.66	-1.80	1.21E-14	9.64E-14

SLFN13	0.66	1.78	2.08E-19	2.50E-18
DOC2A	0.66	-0.72	2.10E-17	2.14E-16
ZNF467	0.65	0.86	5.41E-14	4.03E-13
MYL12A	0.65	11.09	3.37E-18	3.66E-17
LY9	0.65	-1.20	1.08E-13	7.76E-13
RP11-159D12.5	0.65	3.48	7.42E-22	1.10E-20
RARG	0.65	3.13	5.19E-31	1.50E-29
ZCCHC18	0.65	-0.36	1.70E-24	3.07E-23
PIWIL4	0.65	0.99	8.84E-28	2.05E-26
ST6GAL2	0.65	-0.13	7.58E-09	3.33E-08
TPSB2	0.65	0.99	7.75E-06	2.31E-05
RELB	0.65	1.36	7.49E-12	4.51E-11
LRMP	0.65	1.10	3.42E-17	3.42E-16
RP11-763B22.3	0.65	-1.80	3.96E-10	2.00E-09
KIF7	0.65	2.09	9.72E-21	1.31E-19
NPIPB6	0.65	-1.65	2.50E-05	6.91E-05
CSRP3	0.65	10.18	2.80E-19	3.33E-18
OAS2	0.65	3.60	5.26E-24	9.17E-23
LIME1	0.65	-0.05	6.90E-09	3.05E-08
IFI27L2	0.65	3.64	9.59E-11	5.17E-10
UPK3B	0.65	-1.02	3.01E-09	1.39E-08
SORBS2	0.65	9.78	6.94E-27	1.49E-25
DCHS1	0.65	4.10	1.60E-22	2.51E-21
RRP7BP	0.65	3.04	3.06E-31	8.92E-30
TMEM98	0.65	3.29	3.08E-30	8.45E-29
BST2	0.65	4.66	6.58E-14	4.85E-13
PRELP	0.65	6.40	5.66E-12	3.45E-11
EGFL7	0.65	5.12	5.61E-07	1.94E-06
LYRM9	0.65	2.88	4.09E-32	1.26E-30
RAB40A	0.65	-1.06	6.92E-17	6.74E-16
ADAMTS8	0.65	-0.17	2.73E-10	1.40E-09
KIF26A	0.64	2.27	1.11E-13	7.97E-13
PLAGL1	0.64	4.26	4.05E-13	2.76E-12
DUSP27	0.64	7.18	1.02E-24	1.87E-23
LAMA4	0.64	6.43	4.28E-37	1.95E-35
APOBEC3F	0.64	0.56	1.55E-33	5.44E-32
LAT	0.64	1.12	3.69E-20	4.73E-19
EIF3EP1	0.64	-1.38	2.42E-10	1.25E-09
SH3GL2	0.64	2.29	2.85E-09	1.32E-08
BMP6	0.64	2.08	9.22E-15	7.40E-14
CDC42EP5	0.64	0.66	8.85E-09	3.85E-08
NME3	0.64	2.40	1.33E-05	3.84E-05
NPAS3	0.64	-0.18	9.04E-21	1.23E-19
MTMR11	0.64	2.15	1.13E-21	1.65E-20
IDUA	0.64	1.50	2.88E-12	1.82E-11
ACP5	0.64	0.99	6.80E-07	2.32E-06
NXNL1	0.64	-2.02	5.54E-11	3.06E-10
PCYOX1L	0.64	2.52	1.29E-41	8.06E-40
HMGN1	0.64	5.95	3.51E-41	2.14E-39
ADAM33	0.64	4.04	7.24E-18	7.68E-17
TMEM231	0.64	2.89	1.64E-37	7.68E-36
IFI44	0.64	3.89	8.08E-23	1.29E-21
CYP2D7	0.64	-2.11	3.43E-07	1.22E-06
IRF7	0.64	1.84	2.44E-08	1.01E-07
RP4-583P15.15	0.64	0.38	6.31E-10	3.11E-09
TF	0.64	1.38	9.15E-06	2.70E-05
FAM86B1	0.64	0.31	2.94E-21	4.16E-20
NCS1	0.64	2.32	3.04E-16	2.78E-15
CCDC87	0.64	-2.67	4.22E-12	2.61E-11
RPSAP18	0.64	-2.63	7.40E-11	4.04E-10
MYH11	0.64	6.44	2.40E-15	2.02E-14
AFF3	0.64	2.83	2.12E-25	4.12E-24
OSBPL7	0.64	2.34	3.06E-28	7.35E-27
MMP28	0.64	1.11	1.23E-12	8.03E-12
SLC27A3	0.64	3.15	3.16E-21	4.46E-20
GTF2IP13	0.64	1.46	2.42E-20	3.15E-19
SCX	0.64	-0.08	3.20E-05	8.71E-05
IGF2	0.64	5.96	1.17E-17	1.22E-16
FZD7	0.64	4.22	6.30E-25	1.18E-23
ABCA10	0.64	3.88	1.82E-13	1.28E-12
RANBP17	0.64	1.36	2.98E-21	4.21E-20
NEK11	0.63	1.06	3.23E-22	4.94E-21
DLEC1	0.63	-0.83	1.62E-14	1.27E-13
NBEAP3	0.63	-2.27	4.24E-09	1.92E-08
TRBC2	0.63	0.61	8.14E-09	3.55E-08
WBP1	0.63	4.77	1.19E-37	5.61E-36
GAB3	0.63	5.23	7.87E-31	2.25E-29
ST8SIA5	0.63	3.22	8.42E-19	9.64E-18
S1PR4	0.63	-1.89	7.80E-06	2.33E-05
SPATA4	0.63	-1.63	1.06E-10	5.70E-10
PRH1	0.63	-2.18	5.59E-05	0.000146827
RP11-756A22.7	0.63	-1.12	8.58E-07	2.89E-06
AOAH	0.63	0.99	3.97E-11	2.23E-10

GBP1P1	0.63	0.15	4.18E-17	4.15E-16
CHTF18	0.63	0.72	3.65E-10	1.85E-09
FAM155A	0.63	-0.76	3.58E-12	2.23E-11
HEPH	0.63	4.81	2.25E-23	3.76E-22
VCAN	0.63	5.79	3.12E-09	1.43E-08
MSH4	0.63	-0.97	2.17E-07	7.95E-07
BTN3A2	0.63	4.17	4.96E-22	7.43E-21
HES4	0.63	1.63	2.90E-07	1.04E-06
DNAH6	0.63	-1.73	2.75E-10	1.41E-09
RHCE	0.62	-0.88	4.50E-12	2.77E-11
RP11-407P18.1	0.62	-2.30	2.78E-08	1.14E-07
RPL39P40	0.62	-1.17	5.94E-17	5.82E-16
FAM86C2P	0.62	-0.29	1.61E-26	3.38E-25
SLC25A41	0.62	-1.78	8.10E-09	3.54E-08
PHYHIPL	0.62	-1.57	3.56E-05	9.62E-05
DCX	0.62	-2.71	9.26E-11	4.99E-10
TRIP6	0.62	3.57	3.22E-16	2.93E-15
WEE1	0.62	5.87	2.57E-29	6.63E-28
MGP	0.62	8.09	3.79E-14	2.85E-13
PLXNC1	0.62	2.88	4.45E-16	3.99E-15
RFX8	0.62	-1.56	4.97E-09	2.23E-08
NAALADL2	0.62	1.66	1.64E-16	1.54E-15
SCN4B	0.62	4.35	1.03E-20	1.38E-19
PLA2G4C	0.62	5.02	8.01E-31	2.29E-29
OTUD1	0.62	4.94	1.46E-09	6.94E-09
FAR2P2	0.62	1.74	1.07E-27	2.46E-26
LILRA3	0.62	-2.58	2.56E-05	7.07E-05
HYDIN	0.62	0.44	1.83E-17	1.88E-16
SUSD3	0.62	-2.34	3.53E-08	1.43E-07
TNFRSF14	0.62	3.69	1.07E-13	7.73E-13
PRDM8	0.62	0.58	1.67E-20	2.21E-19
SLITRK6	0.62	-1.01	3.35E-06	1.05E-05
LINC00176	0.62	-1.45	3.17E-13	2.17E-12
FBXL22	0.62	2.86	2.02E-13	1.41E-12
PRKG2	0.62	-2.14	8.62E-07	2.90E-06
SH3D21	0.62	0.64	1.32E-18	1.48E-17
CD200	0.62	1.70	2.49E-14	1.92E-13
VWA3B	0.62	-1.63	2.58E-13	1.79E-12
JAM2	0.62	5.16	3.81E-39	2.01E-37
ODC1	0.62	5.83	1.78E-33	6.21E-32
AJAP1	0.62	-1.45	5.89E-09	2.62E-08
TEKT3	0.62	-1.92	6.95E-13	4.64E-12
PCNX2	0.61	1.76	2.24E-06	7.17E-06
ATP2A1	0.61	-0.79	1.79E-18	1.99E-17
SLC22A18	0.61	2.28	2.34E-09	1.09E-08
MLLT11	0.61	5.61	1.31E-12	8.54E-12
ADGRL3	0.61	3.13	1.00E-11	5.96E-11
PRR5	0.61	0.62	1.20E-11	7.08E-11
PLAC8	0.61	-0.83	1.61E-13	1.14E-12
SGCE	0.61	4.24	4.88E-27	1.06E-25
RP11-163E9.2	0.61	0.39	1.71E-22	2.68E-21
PCDH15	0.61	-1.37	2.82E-08	1.16E-07
KLHL33	0.61	0.98	1.04E-18	1.18E-17
RNASET2	0.61	3.38	1.22E-20	1.63E-19
SPAG8	0.61	1.10	1.07E-23	1.82E-22
AC026248.1	0.61	-1.53	1.45E-08	6.17E-08
OTOA	0.61	-2.44	1.13E-08	4.86E-08
RUNX3	0.61	0.46	3.31E-09	1.52E-08
FIBIN	0.61	4.22	1.03E-14	8.25E-14
SNTB1	0.61	2.95	2.03E-23	3.40E-22
STARD5	0.61	1.62	9.69E-20	1.21E-18
PRMT2	0.61	5.44	2.03E-53	3.01E-51
MARVELD1	0.61	3.62	1.37E-22	2.16E-21
NLGN4Y	0.61	-1.36	0.00407974	0.007812466
RP11-266K4.1	0.61	-0.95	4.57E-07	1.59E-06
BSN	0.61	0.02	2.47E-19	2.95E-18
MAMSTR	0.61	0.37	2.50E-12	1.58E-11
HSD17B8	0.61	3.13	5.40E-21	7.45E-20
DHRS11	0.61	3.40	3.39E-48	3.55E-46
FAM35BP	0.61	0.04	9.02E-15	7.25E-14
SCRN2	0.61	4.06	1.59E-16	1.50E-15
SLN	0.61	1.57	1.08E-05	3.16E-05
VSTM5	0.61	-2.48	1.12E-09	5.40E-09
MMP15	0.61	5.15	3.57E-11	2.01E-10
COL4A5	0.61	3.66	5.16E-13	3.49E-12
IL17RD	0.61	2.90	1.95E-19	2.35E-18
SLC22A17	0.61	2.24	6.00E-14	4.44E-13
RAET1G	0.61	-2.10	4.72E-07	1.64E-06
RP11-562I5.1	0.61	-1.04	1.24E-08	5.32E-08
ARSE	0.61	-1.57	1.38E-16	1.30E-15
RNASEK-C17orf49	0.61	-1.92	4.42E-05	0.000118088
GDPD5	0.61	2.05	4.28E-12	2.64E-11
DDAH1	0.61	4.36	4.88E-12	2.99E-11

PARP15	0.61	3.31	1.10E-13	7.90E-13
ARHGDIG	0.61	1.04	5.03E-07	1.75E-06
GFRA2	0.61	0.11	3.09E-13	2.13E-12
NPY5R	0.61	-2.52	1.01E-06	3.36E-06
PMS2P4	0.61	-0.48	4.48E-26	9.12E-25
CRMP1	0.61	2.10	1.14E-29	3.02E-28
ENPP3	0.61	-1.83	1.30E-11	7.63E-11
CTAGE3P	0.61	-2.53	2.86E-08	1.17E-07
CLEC9A	0.61	-2.30	2.85E-08	1.17E-07
FAM65B	0.60	2.68	1.06E-14	8.43E-14
LRP3	0.60	2.94	4.24E-08	1.70E-07
GRID1	0.60	0.45	5.90E-15	4.81E-14
NLRC3	0.60	2.04	3.09E-39	1.65E-37
CXCL12	0.60	6.79	4.11E-19	4.82E-18
DALRD3	0.60	3.07	5.76E-28	1.35E-26
SHANK2	0.60	-2.33	6.56E-11	3.61E-10
TMEM121	0.60	-2.40	4.01E-05	0.000107608
HMGB1P3	0.60	-2.26	3.85E-05	0.000103654
AL358852.1	0.60	-1.76	2.60E-15	2.18E-14
EVC	0.60	2.42	5.04E-08	2.00E-07
SHB	0.60	3.62	1.60E-26	3.35E-25
NDN	0.60	4.74	1.03E-33	3.68E-32
CPE	0.60	5.87	1.17E-21	1.71E-20
HMGN2P15	0.60	-1.62	9.39E-06	2.77E-05
CLDN1	0.60	-1.32	1.17E-05	3.41E-05
RASAL3	0.60	0.13	4.51E-10	2.26E-09
KIF22	0.60	4.19	1.61E-13	1.14E-12
MEOX2	0.60	3.30	1.62E-19	1.97E-18
SGIP1	0.60	3.12	2.56E-11	1.46E-10
DLK2	0.60	-0.51	4.10E-09	1.86E-08
APBA1	0.60	1.84	2.46E-17	2.49E-16
TAGAP	0.60	0.17	3.49E-12	2.18E-11
ZMYND10	0.60	-1.64	1.90E-14	1.48E-13
CAPN3	0.60	3.58	3.46E-31	1.01E-29
SRPK3	0.60	4.34	2.29E-14	1.77E-13
DNMT3B	0.60	0.58	3.32E-20	4.28E-19
FBXO16	0.60	-0.37	2.24E-16	2.08E-15
MGMT	0.60	2.76	4.43E-10	2.23E-09
EPSTI1	0.60	2.28	4.70E-16	4.20E-15
DHRS1	0.60	2.77	9.38E-33	3.06E-31
ARMC9	0.60	1.27	2.94E-33	9.99E-32
TMEM74B	0.60	1.65	6.76E-17	6.59E-16
FRMPD4	0.60	1.80	4.21E-10	2.12E-09
NBPF25P	0.59	-0.33	2.04E-15	1.72E-14
LINGO1	0.59	-0.12	4.49E-11	2.51E-10
RASSF10	0.59	-2.71	3.09E-07	1.10E-06
LRRC1	0.59	2.78	3.21E-25	6.13E-24
HHIPL1	0.59	0.99	1.16E-24	2.13E-23
HERC6	0.59	4.19	8.00E-27	1.71E-25
SAMD12	0.59	3.38	7.68E-15	6.21E-14
PRCD	0.59	1.09	4.13E-23	6.75E-22
BORCS8-MEF2B	0.59	-0.34	1.69E-07	6.26E-07
SSPO	0.59	-0.79	5.29E-11	2.93E-10
TTLL1	0.59	2.47	5.33E-32	1.63E-30
PLA2G7	0.59	-0.95	1.71E-05	4.86E-05
LMOD2	0.59	9.73	9.03E-17	8.67E-16
PABPC1P4	0.59	-1.00	4.83E-11	2.69E-10
LGI1	0.59	-1.51	3.17E-11	1.80E-10
FLT4	0.59	3.23	1.43E-22	2.24E-21
TRANK1	0.59	3.24	2.64E-14	2.02E-13
RCN1P2	0.59	1.65	1.15E-16	1.10E-15
KLRC1	0.59	-2.21	2.62E-07	9.49E-07
PHGDH	0.59	1.95	2.51E-11	1.44E-10
DAPK3	0.59	5.22	6.15E-15	5.01E-14
RP5-1052I5.2	0.59	2.71	2.72E-17	2.74E-16
EGFL8	0.59	2.68	6.80E-25	1.27E-23
ICOSLG	0.59	2.19	2.05E-16	1.91E-15
ABHD14A-ACY1	0.59	-0.64	1.03E-08	4.46E-08
PDLIM7	0.59	4.31	5.39E-09	2.41E-08
RPL32P29	0.59	0.50	1.20E-11	7.06E-11
TMEM176B	0.59	4.87	4.34E-11	2.43E-10
TMEM179	0.59	-2.14	9.70E-07	3.24E-06
SLC16A5	0.59	2.71	2.25E-20	2.94E-19
VWA1	0.59	2.67	1.92E-06	6.20E-06
DUSP8	0.59	3.38	1.37E-14	1.08E-13
BST1	0.59	1.86	6.42E-22	9.56E-21
NOXA1	0.59	0.63	1.33E-05	3.85E-05
CEMIP	0.59	-0.02	5.13E-07	1.78E-06
PSRC1	0.59	0.12	8.28E-14	6.04E-13
AKR7A3	0.59	-2.48	4.57E-10	2.29E-09
MOV10L1	0.59	-2.24	1.33E-07	4.97E-07
CRB1	0.59	-0.95	2.16E-09	1.01E-08
ANKRD35	0.59	0.59	1.49E-12	9.65E-12

CYP2T1P	0.59	-0.35	1.47E-15	1.26E-14
CD69	0.58	-0.48	9.57E-06	2.82E-05
PRAM1	0.58	-0.48	6.13E-11	3.38E-10
WT1	0.58	1.67	3.52E-18	3.81E-17
PAQR7	0.58	1.52	4.08E-24	7.14E-23
COL6A2	0.58	6.91	2.47E-08	1.02E-07
ZNF441	0.58	3.39	5.21E-13	3.52E-12
ADAM20P1	0.58	-0.24	1.61E-15	1.38E-14
PLXDC2	0.58	5.41	6.56E-17	6.40E-16
GABRA4	0.58	4.58	2.96E-10	1.51E-09
PLCH1	0.58	-0.38	1.66E-06	5.38E-06
LIG1	0.58	1.58	2.76E-27	6.18E-26
TMEM191C	0.58	-0.78	8.53E-13	5.67E-12
METRN	0.58	3.26	1.12E-09	5.40E-09
EFEMP2	0.58	5.32	5.87E-19	6.80E-18
TMEM74	0.58	-0.24	3.80E-07	1.34E-06
ZNF177	0.58	1.74	1.06E-11	6.27E-11
PRDM1	0.58	1.30	3.54E-12	2.21E-11
ISYNA1	0.58	3.55	2.41E-11	1.38E-10
C16orf86	0.58	0.62	4.53E-08	1.81E-07
DNALI1	0.58	1.77	6.95E-25	1.30E-23
MCM2	0.58	2.48	6.08E-33	2.02E-31
NOS2	0.58	-2.26	4.20E-06	1.30E-05
GSN	0.58	9.69	1.59E-17	1.64E-16
MYO1D	0.58	3.84	1.00E-15	8.71E-15
MAGED2	0.58	6.48	8.72E-55	1.43E-52
C1QTNF9B	0.58	0.31	3.08E-12	1.93E-11
SIDT1	0.58	0.39	1.49E-20	1.98E-19
PAGR1	0.58	3.55	3.96E-21	5.54E-20
CLIP3	0.58	2.06	2.49E-20	3.24E-19
CTSF	0.58	5.47	3.19E-22	4.89E-21
INPP4B	0.58	5.42	1.43E-10	7.60E-10
RASIP1	0.58	3.68	6.39E-16	5.64E-15
C19orf66	0.58	3.50	1.92E-33	6.67E-32
ASPA	0.58	1.99	1.70E-16	1.60E-15
ST8SIA1	0.58	1.12	4.44E-20	5.66E-19
UBE2L6	0.58	3.68	8.32E-27	1.78E-25
NR1D1	0.58	3.24	2.32E-09	1.08E-08
GRIA3	0.58	0.92	2.80E-06	8.84E-06
GPRASP2	0.58	4.25	4.77E-35	1.86E-33
EEF1A1P5	0.58	1.07	3.25E-09	1.49E-08
ZNF575	0.58	-0.71	5.21E-13	3.51E-12
GFAP	0.58	1.95	1.43E-17	1.49E-16
FBXL2	0.58	1.73	1.33E-28	3.25E-27
RAB28P5	0.58	-0.07	4.34E-18	4.66E-17
NR0B2	0.57	1.55	5.77E-10	2.86E-09
KCNQ4	0.57	1.35	2.68E-18	2.93E-17
GALNT12	0.57	-0.15	6.39E-12	3.88E-11
SYTL1	0.57	-0.15	3.14E-12	1.96E-11
HCST	0.57	0.21	2.78E-07	1.00E-06
MFSD4A	0.57	0.42	1.27E-14	1.00E-13
LIN7B	0.57	2.15	2.43E-10	1.25E-09
UCN	0.57	-0.37	3.13E-10	1.60E-09
C10orf71	0.57	5.96	1.74E-14	1.36E-13
SYT12	0.57	-1.06	2.72E-08	1.12E-07
FBXO17	0.57	1.75	7.61E-29	1.89E-27
CTC-435M10.3	0.57	3.88	2.21E-08	9.18E-08
HIST1H3H	0.57	-2.04	1.05E-08	4.53E-08
RAD51AP2	0.57	-1.23	2.36E-09	1.10E-08
CNTN4	0.57	0.72	7.75E-08	3.00E-07
IFITM10	0.57	-2.34	3.21E-06	1.01E-05
ORMDL3	0.57	5.56	2.60E-49	2.88E-47
DAND5	0.57	2.12	9.03E-14	6.55E-13
FAM102B	0.57	3.31	2.72E-14	2.08E-13
DNAJC30	0.57	3.37	6.59E-23	1.06E-21
LGALS4	0.57	-0.75	6.85E-09	3.03E-08
AC108938.5	0.57	-0.32	1.80E-07	6.65E-07
TMEM253	0.57	-2.35	1.90E-09	8.95E-09
EFNB3	0.57	-1.00	2.45E-07	8.90E-07
SEMA3B	0.57	3.56	2.10E-13	1.46E-12
BHLHE22	0.57	-1.57	1.85E-07	6.80E-07
GAMT	0.57	4.15	3.70E-08	1.50E-07
JUN	0.57	5.78	5.58E-18	5.95E-17
GALNT13	0.57	-2.27	6.62E-07	2.26E-06
RP11-368J21.3	0.57	-2.07	6.18E-08	2.43E-07
ZNF365	0.57	0.94	1.21E-18	1.37E-17
ZDHHC1	0.57	1.34	1.76E-15	1.50E-14
RHNO1	0.57	2.26	2.25E-36	9.72E-35
CD27	0.57	0.40	1.58E-14	1.24E-13
ATPIF1	0.57	6.77	1.23E-20	1.64E-19
C16orf74	0.56	-1.21	8.40E-10	4.11E-09
NAALADL1	0.56	0.87	3.18E-15	2.65E-14
ZBP1	0.56	-0.88	7.29E-14	5.33E-13

BLNK	0.56	-0.33	5.39E-09	2.41E-08
GRIN2B	0.56	-2.67	1.17E-09	5.61E-09
FLT3LG	0.56	0.96	2.80E-18	3.06E-17
ZNF843	0.56	0.82	2.21E-23	3.68E-22
PHLDB2	0.56	7.12	1.22E-12	8.01E-12
GBP4	0.56	4.40	2.84E-16	2.60E-15
FAM161B	0.56	2.80	4.79E-17	4.74E-16
PDE6B	0.56	0.89	2.47E-23	4.10E-22
ENO2	0.56	3.91	1.90E-22	2.95E-21
JMJD4	0.56	3.04	5.07E-17	5.00E-16
LRRC38	0.56	-1.39	0.000512392	0.001156558
IL12A	0.56	-1.13	8.03E-10	3.94E-09
CES1P1	0.56	1.31	0.001847058	0.003779868
COL6A6	0.56	2.22	2.28E-07	8.31E-07
HBEGF	0.56	5.11	2.11E-15	1.78E-14
CDKL2	0.56	-2.69	1.97E-06	6.34E-06
HLA-DOB	0.56	-1.26	3.14E-06	9.86E-06
FSIP1	0.56	-0.45	5.20E-08	2.06E-07
FMNL3	0.56	4.75	1.35E-26	2.84E-25
CCDC61	0.56	1.39	1.10E-10	5.92E-10
ADAMTS16	0.56	-1.96	9.36E-07	3.13E-06
LRRC4B	0.56	-1.71	3.85E-05	0.000103657
MAB21L1	0.56	-0.53	9.47E-14	6.85E-13
CHRNA1	0.56	-1.14	3.65E-07	1.29E-06
CYP21A2	0.56	-1.41	5.28E-06	1.61E-05
ST3GAL6	0.56	5.93	2.54E-26	5.25E-25
PPIAP16	0.56	-1.99	5.29E-07	1.83E-06
TRAF1	0.56	2.78	6.83E-19	7.87E-18
DACT3	0.56	3.55	1.55E-21	2.25E-20
B3GNT5	0.56	2.26	3.21E-09	1.47E-08
HID1	0.56	3.16	3.12E-23	5.14E-22
TAS2R19	0.56	-2.61	2.91E-06	9.16E-06
ATP2A3	0.56	2.66	1.90E-16	1.78E-15
AOC3	0.56	4.24	4.66E-11	2.60E-10
TENM3	0.56	1.93	8.17E-16	7.16E-15
SEPT6	0.56	4.00	5.33E-23	8.64E-22
C14orf79	0.56	1.51	2.69E-26	5.55E-25
ASMTL	0.56	4.28	7.94E-24	1.36E-22
NT5C1A	0.56	1.08	5.84E-09	2.60E-08
ZFP2	0.56	0.71	3.18E-25	6.09E-24
NXPH3	0.56	1.98	1.93E-17	1.97E-16
CMKLR1	0.56	3.78	3.76E-15	3.13E-14
PGBD4P3	0.56	-2.23	2.79E-09	1.29E-08
PLD5	0.56	-1.15	1.08E-07	4.07E-07
KLK14	0.56	-1.27	5.79E-08	2.28E-07
ZSCAN18	0.56	5.47	4.02E-37	1.83E-35
RHBDL2	0.56	0.81	2.58E-14	1.98E-13
BTN3A3	0.55	3.60	1.32E-19	1.62E-18
PNMAL1	0.55	3.41	3.90E-17	3.88E-16
BICC1	0.55	3.70	3.20E-14	2.42E-13
FAM43B	0.55	0.34	8.91E-07	2.99E-06
PLGLB2	0.55	-1.07	0.00203526	0.004133196
BSCL2	0.55	2.23	1.41E-05	4.06E-05
ACAP1	0.55	1.81	1.81E-15	1.54E-14
IQSEC3	0.55	1.17	4.92E-16	4.39E-15
NOX4	0.55	2.08	3.13E-08	1.28E-07
WTIP	0.55	3.99	7.30E-32	2.22E-30
CADPS	0.55	3.94	2.01E-12	1.29E-11
GPCPD1	0.55	5.03	2.57E-22	3.96E-21
AGAP10P	0.55	-0.82	8.68E-08	3.34E-07
RP11-1223D19.1	0.55	-1.45	2.54E-07	9.21E-07
MYOM2	0.55	10.02	6.87E-20	8.66E-19
TNFSF15	0.55	-2.08	3.60E-08	1.46E-07
TMEM8B	0.55	3.40	1.70E-32	5.44E-31
ABCC11	0.55	-2.59	3.54E-09	1.62E-08
S100A14	0.55	0.72	1.08E-09	5.20E-09
CXCR6	0.55	0.02	1.72E-07	6.38E-07
PADI2	0.55	-0.69	9.97E-08	3.79E-07
OPRL1	0.55	-1.18	9.55E-08	3.65E-07
ANKRD65	0.55	0.23	1.78E-07	6.56E-07
CPXM1	0.55	-0.11	0.001029433	0.002195442
CBLN3	0.55	0.24	1.25E-12	8.19E-12
LAG3	0.55	-1.93	1.68E-07	6.22E-07
RP11-104G3.2	0.55	-0.04	7.26E-08	2.83E-07
REC8	0.55	1.71	3.78E-18	4.08E-17
PATZ1	0.55	3.58	5.00E-53	7.04E-51
NFASC	0.55	4.68	1.33E-16	1.26E-15
KLHL34	0.55	1.67	4.71E-14	3.52E-13
TPTE2P2	0.55	-1.12	1.10E-09	5.30E-09
POU2F2	0.55	0.28	2.93E-11	1.67E-10
ALKBH7	0.55	4.39	1.56E-05	4.47E-05
MIEF2	0.55	4.06	3.30E-21	4.65E-20
CMTM3	0.55	3.58	7.22E-17	7.01E-16

NUP210	0.55	2.10	7.10E-22	1.05E-20
UNC5B	0.55	3.92	3.60E-14	2.71E-13
SLC5A4	0.55	-2.20	0.000495318	0.001120552
ANKRD36B	0.55	2.35	4.92E-24	8.61E-23
HHIP	0.55	-0.82	1.98E-08	8.29E-08
TSPAN4	0.54	3.62	6.55E-08	2.57E-07
SLC43A2	0.54	2.00	6.68E-13	4.47E-12
PPFIA4	0.54	5.15	2.03E-15	1.72E-14
IL33	0.54	4.49	3.20E-08	1.30E-07
ROBO1	0.54	3.26	9.03E-15	7.25E-14
ADAMTS10	0.54	2.47	1.57E-14	1.24E-13
RP4-669L17.8	0.54	-1.54	0.001948568	0.003974006
MYCL	0.54	-0.87	4.69E-10	2.34E-09
HLA-F	0.54	4.47	1.53E-18	1.71E-17
RUNX1	0.54	2.74	9.98E-08	3.80E-07
TPTE2P1	0.54	-2.03	3.11E-11	1.77E-10
PRRT3	0.54	-0.29	6.51E-16	5.74E-15
RGS13	0.54	-1.82	2.19E-07	8.01E-07
MEGF10	0.54	-1.78	4.00E-07	1.41E-06
FADS2	0.54	3.65	2.39E-11	1.37E-10
HNRNPA1L2	0.54	2.09	4.29E-27	9.41E-26
EGR3	0.54	1.48	0.00012582	0.000312777
ZNF610	0.54	1.48	8.68E-20	1.09E-18
HCN2	0.54	2.13	3.33E-05	9.03E-05
BCAS4	0.54	1.48	7.34E-27	1.58E-25
CNKSR2	0.54	1.61	3.75E-11	2.11E-10
FGFBP3	0.54	-0.54	1.39E-15	1.20E-14
MTCO2P22	0.54	-1.65	0.003750637	0.007235191
SLC24A3	0.54	0.41	5.33E-12	3.26E-11
M1AP	0.54	-1.91	9.22E-08	3.53E-07
UBBP4	0.54	-1.98	5.08E-09	2.28E-08
TRAIP	0.54	-0.45	7.70E-17	7.44E-16
RP11-7F17.1	0.54	-2.06	0.009235738	0.016399725
VEGFC	0.54	2.76	1.06E-16	1.01E-15
APOBEC3G	0.54	1.83	4.59E-25	8.69E-24
C16orf58	0.54	4.61	1.25E-29	3.29E-28
AF186192.5	0.54	0.59	1.13E-12	7.41E-12
SLC7A9	0.54	-2.52	6.01E-09	2.67E-08
CFAP53	0.54	-2.06	1.42E-10	7.54E-10
CFAP70	0.54	2.58	9.43E-18	9.93E-17
CHCHD6	0.54	2.04	8.45E-15	6.81E-14
CSF1R	0.54	4.51	2.13E-13	1.49E-12
BBC3	0.54	0.29	2.37E-09	1.10E-08
NME5	0.54	1.32	2.78E-17	2.80E-16
AC098614.2	0.54	-0.26	7.55E-13	5.03E-12
TEX22	0.54	-2.37	2.33E-07	8.50E-07
DCST2	0.54	-1.21	1.89E-10	9.91E-10
CTC-359D24.3	0.54	0.30	1.02E-16	9.72E-16
STK38L	0.54	6.29	1.59E-10	8.38E-10
DNASE1L2	0.54	-2.22	3.45E-06	1.08E-05
ANKRD18B	0.54	-1.06	1.18E-10	6.33E-10
FAM173A	0.54	1.15	0.00024104	0.000573172
HAS3	0.54	-0.27	2.35E-10	1.22E-09
ALDH4A1	0.53	5.12	4.37E-22	6.58E-21
MORN4	0.53	3.20	1.13E-33	4.02E-32
TMEM240	0.53	-0.71	2.67E-06	8.45E-06
C6orf1	0.53	3.11	3.96E-12	2.45E-11
C9orf43	0.53	-1.82	1.53E-11	8.93E-11
DNPH1	0.53	2.41	1.02E-08	4.39E-08
ZNF775	0.53	1.60	4.79E-15	3.94E-14
GABRB1	0.53	-0.09	1.20E-08	5.13E-08
POLR1E	0.53	3.58	9.29E-34	3.33E-32
RNF112	0.53	1.05	2.82E-13	1.95E-12
NYNRIN	0.53	3.78	1.40E-27	3.20E-26
MTRNR2L11	0.53	7.98	3.01E-07	1.08E-06
PXDNL	0.53	6.03	4.01E-18	4.32E-17
TMEM232	0.53	-0.74	1.32E-10	7.05E-10
OLFML2B	0.53	1.69	2.73E-07	9.84E-07
CRY2	0.53	6.27	2.46E-37	1.14E-35
GAPT	0.53	-0.63	5.73E-07	1.98E-06
NUDT17	0.53	1.20	1.91E-21	2.75E-20
MX2	0.53	3.64	2.31E-22	3.58E-21
MYO10	0.53	3.62	1.20E-21	1.75E-20
JAM3	0.53	5.44	2.28E-50	2.71E-48
BRF2	0.53	3.67	9.76E-29	2.40E-27
PSMB9	0.53	2.87	5.51E-16	4.90E-15
ATP13A4	0.53	-2.50	4.37E-08	1.75E-07
PTPRVP	0.53	-2.14	9.05E-10	4.40E-09
ISM1	0.53	0.05	3.96E-09	1.80E-08
COL9A3	0.53	0.18	9.73E-06	2.86E-05
C19orf60	0.53	3.31	8.28E-07	2.80E-06
MIB2	0.53	4.02	1.51E-06	4.91E-06
GPX8	0.53	3.09	5.57E-12	3.40E-11

KMT5C	0.53	1.42	6.08E-12	3.70E-11
MAATS1	0.53	0.22	8.14E-14	5.93E-13
MYO15B	0.53	5.01	2.10E-13	1.47E-12
TRIM73	0.53	0.88	3.92E-21	5.49E-20
TMEM156	0.53	-1.68	4.68E-09	2.11E-08
SLC16A8	0.53	-1.70	1.33E-06	4.36E-06
TTLL7	0.53	3.07	1.87E-08	7.83E-08
UPK3BL	0.53	2.79	0.001881494	0.00384658
ABHD16B	0.52	-1.07	0.000426973	0.000977022
CLEC3B	0.52	4.83	1.22E-05	3.52E-05
PPIEL	0.52	-0.17	1.96E-17	2.00E-16
SHF	0.52	1.65	7.04E-24	1.21E-22
ITGA8	0.52	3.15	5.00E-08	1.99E-07
SPATA20	0.52	5.45	1.41E-17	1.46E-16
FBXO44	0.52	3.59	3.99E-18	4.30E-17
PACS1	0.52	5.55	1.10E-29	2.93E-28
FST	0.52	1.59	1.58E-08	6.67E-08
NANOS3	0.52	-2.25	5.61E-06	1.70E-05
AC073333.1	0.52	0.56	2.78E-08	1.14E-07
TCTEX1D2	0.52	0.92	6.14E-15	5.00E-14
TMEM42	0.52	2.97	1.29E-32	4.16E-31
C4BPA	0.52	-2.62	7.51E-09	3.30E-08
C5orf56	0.52	3.30	1.57E-30	4.41E-29
UNC13D	0.52	2.08	4.67E-09	2.11E-08
SLC2A4RG	0.52	4.38	3.69E-09	1.68E-08
TRAF5	0.52	3.06	2.72E-16	2.51E-15
AGBL2	0.52	-1.98	2.85E-09	1.31E-08
KIF13A	0.52	6.83	6.44E-23	1.04E-21
SOHLH2	0.52	-0.75	4.30E-05	0.000114926
SLC16A11	0.52	-1.94	1.04E-05	3.05E-05
SLAMF6	0.52	-0.38	1.06E-07	4.02E-07
TSPOAP1	0.52	3.61	1.37E-12	8.92E-12
SELP	0.52	2.72	1.16E-13	8.32E-13
FXYD6	0.52	5.20	3.65E-27	8.06E-26
TKFC	0.52	2.91	6.76E-23	1.09E-21
DYNC2LI1	0.52	3.53	7.85E-36	3.25E-34
D2HGDH	0.52	3.25	2.73E-10	1.40E-09
KRTAP5-1	0.52	-2.65	0.000100807	0.000254525
ELOVL4	0.52	-0.95	2.39E-07	8.69E-07
GSDMD	0.52	3.54	1.76E-08	7.43E-08
WHRN	0.52	-0.87	1.01E-09	4.88E-09
SPEF2	0.52	2.05	2.03E-11	1.17E-10
NFKB2	0.52	3.17	7.33E-17	7.11E-16
CFH	0.52	6.35	1.47E-09	6.98E-09
LRRC49	0.52	5.91	1.73E-20	2.29E-19
LENG9	0.52	0.23	0.00052786	0.001188075
RPS28P7	0.52	2.11	3.41E-06	1.07E-05
TMEM59L	0.52	0.17	2.63E-05	7.24E-05
PPP1R3C	0.52	6.81	1.14E-14	9.06E-14
RPS2P32	0.52	0.51	1.38E-12	9.00E-12
EID3	0.52	-0.53	2.66E-12	1.68E-11
GLIPR1L2	0.52	1.42	3.13E-13	2.15E-12
DUSP26	0.52	5.62	1.26E-15	1.09E-14
ARMC5	0.52	2.21	1.02E-09	4.93E-09
WIPF3	0.52	3.00	2.21E-24	3.95E-23
KRT80	0.52	-1.80	1.01E-05	2.97E-05
TTC23L	0.52	-2.35	5.02E-09	2.26E-08
CERKL	0.52	0.71	2.11E-10	1.10E-09
LINC00521	0.52	-0.89	2.90E-05	7.93E-05
C21orf33	0.52	4.24	0.005043505	0.009470706
AP006285.1	0.52	-0.82	5.93E-17	5.82E-16
AP006285.2	0.52	-0.82	5.93E-17	5.82E-16
XPO4	0.51	5.79	5.14E-12	3.14E-11
TMEM200C	0.51	-0.74	8.90E-09	3.87E-08
TIAM1	0.51	1.00	1.01E-11	6.04E-11
FBXO24	0.51	0.12	6.58E-22	9.78E-21
NRIP3	0.51	0.12	5.51E-13	3.71E-12
ACKR4	0.51	1.82	1.95E-06	6.28E-06
IL1B	0.51	-1.35	0.005451698	0.010159763
MASP1	0.51	6.93	3.75E-19	4.41E-18
OMG	0.51	-1.23	1.59E-07	5.90E-07
RP11-75L1.2	0.51	-0.54	2.39E-05	6.64E-05
FAM9A	0.51	-2.47	2.78E-07	1.00E-06
MMP23B	0.51	2.54	0.000330476	0.000768709
NEK5	0.51	0.73	1.33E-16	1.26E-15
VPS37D	0.51	-0.44	1.15E-06	3.79E-06
NOTCH2NL	0.51	3.26	3.88E-14	2.92E-13
HSF4	0.51	3.77	5.69E-11	3.14E-10
ZNF135	0.51	2.50	3.40E-33	1.15E-31
ARHGAP39	0.51	-0.30	6.42E-09	2.85E-08
FANCG	0.51	1.58	4.69E-21	6.49E-20
NEURL1	0.51	-2.12	4.42E-10	2.22E-09
RET	0.51	-0.21	1.26E-06	4.15E-06

FCHSD1	0.51	2.39	1.34E-20	1.79E-19
ALS2CR12	0.51	2.13	3.00E-15	2.51E-14
ZNF618	0.51	2.01	7.12E-17	6.93E-16
RP3-454G6.2	0.51	-2.11	2.65E-09	1.23E-08
BAALC	0.51	1.31	1.35E-16	1.28E-15
AVPR2	0.51	1.09	4.69E-06	1.44E-05
CATSPER2	0.51	1.91	1.16E-14	9.20E-14
CFAP221	0.51	-0.10	7.30E-17	7.08E-16
SEMA4C	0.51	4.23	6.65E-19	7.68E-18
TPTE2P6	0.51	-2.01	6.82E-08	2.67E-07
SOX4	0.51	2.90	2.60E-11	1.49E-10
IL7R	0.51	1.47	3.25E-05	8.84E-05
KDELR3	0.51	3.05	4.58E-14	3.43E-13
TNFRSF10C	0.51	-0.21	1.70E-11	9.88E-11
EPS8L2	0.51	0.19	1.47E-05	4.21E-05
PRSS30P	0.51	-2.60	7.20E-05	0.000185999
NHSL1	0.51	4.73	4.34E-32	1.34E-30
PDE8B	0.51	2.70	1.84E-06	5.94E-06
UBE2H	0.51	7.45	3.25E-27	7.22E-26
PTPRF	0.51	1.91	2.80E-06	8.82E-06
RHEBL1	0.50	-1.21	1.70E-11	9.88E-11
TBXA2R	0.50	0.50	9.84E-12	5.87E-11
ZNF853	0.50	3.48	2.08E-25	4.06E-24
PPP1R15A	0.50	6.11	5.31E-29	1.33E-27
NLRC5	0.50	3.48	1.94E-16	1.81E-15
CPED1	0.50	4.79	1.57E-08	6.64E-08
RABGAP1L	0.50	6.98	5.29E-24	9.20E-23
DDX60	0.50	3.58	3.61E-10	1.83E-09
RPL4P5	0.50	-1.29	1.25E-06	4.12E-06
ZNF114	0.50	-1.04	1.05E-14	8.40E-14
CISH	0.50	3.50	3.28E-09	1.50E-08
ZNF251	0.50	4.06	6.38E-20	8.04E-19
AC008810.1	0.50	-0.06	4.95E-06	1.51E-05
AL135745.1	0.50	-0.06	4.95E-06	1.51E-05
CATSPERG	0.50	0.03	2.00E-20	2.62E-19
PER3	0.50	4.15	1.22E-08	5.20E-08
C1orf35	0.50	2.36	2.02E-15	1.71E-14
MAMDC4	0.50	2.48	1.66E-06	5.40E-06
DKK2	0.50	0.23	0.000133553	0.000330381
SV2C	0.50	-1.43	1.34E-05	3.88E-05
MMP17	0.50	-0.99	1.34E-06	4.40E-06
HSFX2	0.50	-2.14	0.017566317	0.029328877
DGKB	0.50	-0.97	9.90E-08	3.77E-07
PELP1	0.50	4.45	2.74E-16	2.52E-15
TRPC6	0.50	0.01	4.75E-08	1.89E-07
TSHZ2	0.50	2.37	6.80E-14	4.99E-13
PCSK4	0.50	-1.86	2.00E-05	5.61E-05
PTPRN2	0.50	1.73	1.01E-22	1.60E-21
PROS1	0.50	6.92	4.53E-10	2.27E-09
PLA2G4B	0.50	1.99	2.36E-07	8.60E-07
ITPK1	-0.50	5.72	3.48E-23	5.72E-22
SLC41A1	-0.50	8.27	4.38E-20	5.59E-19
ITGA11	-0.50	4.04	3.69E-14	2.77E-13
FAM83D	-0.50	-0.73	2.47E-05	6.84E-05
LIPG	-0.50	0.37	1.46E-06	4.76E-06
CDKN1A	-0.50	6.17	2.65E-11	1.51E-10
GPATCH4	-0.50	4.22	6.09E-33	2.02E-31
ATP6V1C2	-0.50	1.13	1.27E-14	1.00E-13
NOD2	-0.50	-0.03	4.54E-10	2.28E-09
CCT8P1	-0.50	-0.71	2.22E-06	7.11E-06
FABP5P7	-0.50	3.32	0.027500515	0.044062906
SLC7A7	-0.50	2.34	5.51E-12	3.36E-11
FURIN	-0.51	5.35	1.16E-16	1.11E-15
FCAR	-0.51	-0.23	4.43E-06	1.36E-05
MS4A6A	-0.51	4.74	5.76E-10	2.85E-09
ASB15	-0.51	5.58	2.30E-06	7.34E-06
LARP1B	-0.51	4.27	4.56E-18	4.89E-17
G0S2	-0.51	4.34	1.70E-05	4.83E-05
AK9	-0.51	2.98	5.55E-17	5.45E-16
KLHL23	-0.51	3.84	4.63E-13	3.14E-12
ZNF367	-0.51	2.18	6.31E-09	2.81E-08
KLHL2	-0.51	3.59	2.83E-12	1.78E-11
FOXF1	-0.51	-1.94	1.15E-05	3.35E-05
SLC39A14	-0.51	7.14	8.33E-20	1.04E-18
NR4A2	-0.51	1.93	0.00100809	0.00215349
BATF	-0.51	-1.57	0.000111952	0.000280843
DLGAP5	-0.51	-2.14	0.000119492	0.000298195
LOC100421166	-0.51	-2.26	0.002270427	0.004568674
MAOA	-0.51	7.15	1.48E-19	1.81E-18
SUN2	-0.51	6.38	2.62E-24	4.65E-23
STK26	-0.51	0.93	1.12E-06	3.71E-06
H2AFZ	-0.51	5.29	2.61E-24	4.63E-23
SRCAP	-0.51	4.39	1.63E-05	4.65E-05

FAM105A	-0.51	1.53	4.02E-09	1.82E-08
PLCL1	-0.51	6.54	7.79E-12	4.67E-11
TMEM236	-0.51	0.86	3.60E-13	2.46E-12
ABCB6	-0.51	3.74	1.52E-27	3.46E-26
BMS1P10	-0.52	0.52	1.16E-11	6.88E-11
ATP1B3	-0.52	5.14	8.79E-18	9.27E-17
RP11-188C12.2	-0.52	-1.51	6.63E-08	2.59E-07
HADHB	-0.52	9.70	7.57E-33	2.49E-31
KIF24	-0.52	0.45	1.59E-11	9.25E-11
CLSPN	-0.52	-1.40	5.66E-07	1.95E-06
GPR180	-0.52	3.30	1.59E-10	8.38E-10
PHTF2	-0.52	4.97	4.13E-11	2.32E-10
KIAA1456	-0.52	4.38	9.79E-21	1.32E-19
LRRC10B	-0.52	-0.63	1.47E-08	6.24E-08
TGM2	-0.52	9.07	3.19E-19	3.78E-18
MMP19	-0.52	2.45	2.77E-07	9.99E-07
GFOD1	-0.52	5.64	1.44E-25	2.85E-24
MTFR1	-0.52	4.10	3.28E-15	2.73E-14
ERRFI1	-0.52	4.13	2.88E-10	1.48E-09
PPP1CC	-0.52	7.16	2.10E-28	5.07E-27
B4GALT6	-0.52	2.07	6.92E-09	3.06E-08
PSME4	-0.52	6.80	4.69E-29	1.18E-27
TMEM38B	-0.52	5.41	6.60E-15	5.35E-14
KAT2B	-0.52	6.16	4.91E-15	4.04E-14
GUCY1A2	-0.52	3.95	5.00E-08	1.99E-07
LILRB3	-0.52	0.65	1.56E-06	5.07E-06
CD300LG	-0.52	4.57	3.64E-13	2.49E-12
SLC25A34	-0.52	3.40	4.08E-08	1.64E-07
SSR3	-0.52	7.26	3.78E-28	8.97E-27
LGR4	-0.52	4.97	5.48E-08	2.16E-07
KLF9	-0.52	6.97	2.02E-15	1.71E-14
EFHC2	-0.53	2.83	2.21E-09	1.03E-08
HOXB6	-0.53	-1.60	2.80E-07	1.01E-06
ITGA5	-0.53	6.18	1.69E-17	1.74E-16
MFSD6	-0.53	4.61	1.07E-27	2.47E-26
NSDHL	-0.53	3.82	1.59E-22	2.50E-21
TPPP	-0.53	3.91	4.10E-12	2.53E-11
ANKRD7	-0.53	-2.55	7.51E-09	3.30E-08
bP-2171C21.2	-0.53	-2.15	3.66E-05	9.88E-05
ZBTB7B	-0.53	4.29	3.66E-22	5.58E-21
MEGF9	-0.53	3.93	2.76E-11	1.58E-10
MAP3K6	-0.53	5.20	1.52E-15	1.30E-14
E2F7	-0.53	-2.54	1.80E-05	5.10E-05
MFAP3	-0.53	3.37	1.90E-05	5.35E-05
TACC1	-0.53	6.93	2.07E-12	1.33E-11
PNP	-0.53	3.43	5.45E-12	3.33E-11
LRRC7	-0.53	0.32	3.43E-07	1.22E-06
FMO2	-0.53	5.95	2.20E-07	8.03E-07
TMEM2	-0.53	3.49	4.54E-10	2.28E-09
UBASH3B	-0.53	1.18	5.90E-09	2.63E-08
TLDC2	-0.53	0.00	1.14E-07	4.31E-07
KIF18B	-0.53	-1.01	1.16E-09	5.56E-09
TLR8	-0.53	0.50	2.65E-07	9.57E-07
FAM213A	-0.53	6.39	1.29E-42	8.79E-41
WISP1	-0.53	0.84	4.57E-06	1.41E-05
GMPR	-0.53	5.87	3.34E-30	9.13E-29
PTH1R	-0.53	2.94	4.24E-10	2.13E-09
SAT1	-0.53	7.00	9.37E-17	8.99E-16
ACSL1	-0.53	8.38	7.99E-17	7.70E-16
GFRA1	-0.53	2.70	9.71E-08	3.70E-07
PALM2	-0.53	0.26	4.29E-07	1.50E-06
SMIM3	-0.54	4.54	3.72E-22	5.67E-21
ZFP92	-0.54	0.38	2.68E-18	2.93E-17
PARVB	-0.54	6.07	1.07E-27	2.46E-26
RP11-62J1.3	-0.54	-0.54	0.000821682	0.001783949
CASKIN1	-0.54	-1.72	0.000274296	0.000646652
SLC7A1	-0.54	4.09	2.92E-14	2.23E-13
VCL	-0.54	8.49	1.62E-16	1.52E-15
NUF2	-0.54	-1.58	2.03E-06	6.52E-06
MOB3B	-0.54	1.87	7.02E-12	4.24E-11
MAN1A1	-0.54	5.11	7.47E-09	3.28E-08
PPM1L	-0.54	5.40	2.95E-21	4.18E-20
JPH1	-0.54	3.93	3.37E-15	2.80E-14
CBLB	-0.54	5.83	2.04E-18	2.26E-17
RP11-644F5.10	-0.54	3.43	7.61E-17	7.36E-16
PLXNB1	-0.54	5.17	3.21E-28	7.65E-27
C4orf26	-0.54	-2.28	1.02E-08	4.41E-08
BHMT2	-0.54	2.52	4.82E-10	2.41E-09
TMEFF2	-0.54	-1.34	8.32E-07	2.81E-06
DSP	-0.54	8.64	6.70E-19	7.73E-18
ACR	-0.54	-1.87	4.50E-06	1.39E-05
FOSL2	-0.54	5.85	3.09E-12	1.94E-11
C6orf106	-0.54	7.11	4.99E-64	1.47E-61

CDC42EP4	-0.55	5.34	1.07E-27	2.46E-26
EPHB6	-0.55	1.34	1.29E-11	7.59E-11
TMEM245	-0.55	7.25	1.59E-22	2.50E-21
GPR82	-0.55	-0.98	4.86E-09	2.19E-08
FAM46C	-0.55	3.70	3.07E-17	3.09E-16
NFKBIZ	-0.55	3.90	9.42E-10	4.57E-09
IP6K3	-0.55	4.95	3.02E-14	2.30E-13
ABHD5	-0.55	3.74	1.09E-12	7.17E-12
LYPD5	-0.55	-0.12	5.50E-12	3.36E-11
SLCO5A1	-0.55	3.77	1.30E-14	1.03E-13
UAP1	-0.55	4.72	1.00E-22	1.59E-21
PPP3CC	-0.55	5.18	3.24E-62	8.60E-60
PDCD6IPP1	-0.55	-0.11	2.67E-06	8.44E-06
MFSD2A	-0.55	-1.03	1.89E-06	6.08E-06
NR2E3	-0.55	-2.34	1.36E-10	7.24E-10
KIF14	-0.55	-1.65	4.20E-07	1.47E-06
DHCR24	-0.55	4.22	3.97E-11	2.23E-10
USP53	-0.55	6.21	1.49E-08	6.31E-08
NDUFB5P1	-0.55	-2.55	1.14E-05	3.32E-05
HIST1H2BJ	-0.55	-0.47	1.74E-14	1.36E-13
ESRRG	-0.55	4.66	6.21E-11	3.42E-10
HES6	-0.55	-0.32	2.88E-08	1.18E-07
ACACB	-0.55	7.08	4.26E-21	5.93E-20
IGSF11	-0.55	0.68	6.87E-10	3.38E-09
RAET1E	-0.55	-1.61	1.61E-10	8.48E-10
NANOS1	-0.55	3.06	4.16E-12	2.57E-11
CDH26	-0.55	-0.09	1.49E-14	1.17E-13
SFRP5	-0.55	-0.17	0.000610591	0.0013584
GLUD2	-0.55	-0.83	8.65E-09	3.77E-08
UBN1	-0.56	5.28	9.52E-27	2.03E-25
WWP1	-0.56	6.79	1.67E-21	2.41E-20
ASF1B	-0.56	-1.08	2.92E-09	1.35E-08
CDH22	-0.56	-1.52	8.14E-08	3.15E-07
LILRA5	-0.56	0.10	4.58E-07	1.60E-06
EMB	-0.56	3.67	1.21E-10	6.46E-10
VEPH1	-0.56	-1.99	2.13E-06	6.81E-06
TMED5	-0.56	5.99	1.50E-14	1.18E-13
TBX3	-0.56	4.21	6.26E-25	1.17E-23
ZDHHC9	-0.56	3.93	9.72E-25	1.79E-23
SHISA4	-0.56	5.44	8.20E-21	1.12E-19
BCL2L13	-0.56	6.31	2.16E-44	1.66E-42
PLEKHH1	-0.56	2.48	1.72E-19	2.09E-18
KLK1	-0.56	-2.60	1.49E-05	4.28E-05
RP11-57K17.1	-0.56	-2.66	1.58E-05	4.52E-05
PLCD3	-0.56	4.90	2.10E-22	3.27E-21
NGF	-0.56	1.26	3.76E-17	3.75E-16
SLC2A9	-0.56	1.36	7.54E-26	1.52E-24
FASN	-0.57	3.20	2.01E-05	5.63E-05
SCNN1G	-0.57	-2.54	8.35E-12	5.01E-11
NCOA3	-0.57	5.58	5.33E-21	7.37E-20
CH507-42P11.8	-0.57	3.63	0.001670982	0.003447765
HERC2P9	-0.57	5.47	7.66E-09	3.36E-08
PTPN5	-0.57	2.33	1.93E-08	8.10E-08
SRGN	-0.57	5.78	1.23E-13	8.75E-13
KCNH2	-0.57	5.61	4.38E-20	5.59E-19
ESPL1	-0.57	-1.28	8.40E-08	3.24E-07
HTR1F	-0.57	-2.68	3.18E-06	9.96E-06
PLP2	-0.57	4.85	2.13E-18	2.35E-17
RNF125	-0.57	3.08	1.08E-11	6.43E-11
CLEC4D	-0.57	-2.42	4.59E-05	0.000122067
QRFPR	-0.57	-0.18	5.86E-07	2.02E-06
DLK1	-0.57	1.89	0.001035208	0.002206353
OR6K1P	-0.57	-2.87	8.64E-06	2.56E-05
LGR5	-0.57	-2.29	2.71E-05	7.44E-05
RUNDC3B	-0.57	0.87	2.77E-11	1.58E-10
TRPM7	-0.57	6.04	2.01E-13	1.41E-12
KCNB1	-0.57	3.28	1.38E-15	1.18E-14
PITX3	-0.57	-2.13	0.000131727	0.000326184
HIF1A	-0.57	5.97	1.14E-10	6.13E-10
IMPA2	-0.57	3.79	4.08E-20	5.22E-19
SEMA6B	-0.57	2.36	1.51E-09	7.14E-09
ADRA1A	-0.57	3.17	1.08E-07	4.10E-07
PLS1	-0.57	0.76	2.39E-11	1.37E-10
ZNF749	-0.57	0.51	1.18E-12	7.73E-12
ART5	-0.57	-0.52	7.06E-08	2.75E-07
OPN3	-0.58	2.16	1.71E-24	3.09E-23
BCL9L	-0.58	3.94	5.73E-07	1.98E-06
FAM65C	-0.58	4.04	2.00E-20	2.63E-19
TREM1	-0.58	-0.33	9.00E-07	3.02E-06
PSMC1P1	-0.58	1.17	0.018984246	0.031446412
RCC1	-0.58	2.23	3.45E-23	5.69E-22
CLIC4	-0.58	8.70	7.49E-12	4.51E-11
LIN7A	-0.58	4.17	3.73E-22	5.67E-21

APOD	-0.58	7.89	4.98E-12	3.05E-11
KCNK13	-0.58	-0.93	1.91E-05	5.39E-05
SYN3	-0.58	-1.69	3.08E-13	2.12E-12
TPRKB	-0.58	4.04	1.05E-33	3.72E-32
RPS6KA2	-0.58	6.73	8.31E-35	3.21E-33
SLC25A24	-0.58	4.24	1.57E-11	9.15E-11
RP11-110I1.5	-0.58	-2.53	9.85E-05	0.000249174
RGL3	-0.58	2.68	7.92E-15	6.39E-14
CD300C	-0.58	-1.42	2.43E-07	8.83E-07
LEP	-0.58	0.11	0.017294057	0.028921271
SNAI3	-0.58	-0.14	6.98E-09	3.08E-08
SNTG2	-0.58	-1.35	1.72E-10	9.06E-10
CHPT1	-0.58	6.62	3.04E-35	1.20E-33
LVRN	-0.58	-0.68	7.69E-05	0.000197713
FANCB	-0.58	-2.39	1.88E-09	8.84E-09
ESRP2	-0.58	-1.98	2.31E-11	1.33E-10
UBE2C	-0.59	-2.11	0.000104039	0.00026217
C19orf33	-0.59	0.57	0.000118433	0.00029573
CHGB	-0.59	2.36	8.78E-10	4.28E-09
PTGER3	-0.59	0.52	2.29E-05	6.38E-05
IRAK3	-0.59	4.52	3.37E-11	1.90E-10
SLC22A5	-0.59	4.72	2.13E-17	2.17E-16
GCKR	-0.59	-2.54	1.25E-07	4.68E-07
RP11-449H3.3	-0.59	-2.11	0.000148162	0.000364047
ESR2	-0.59	0.27	5.19E-15	4.25E-14
GPD1	-0.59	3.45	1.54E-05	4.41E-05
JAK1	-0.59	7.26	4.79E-37	2.18E-35
LRP4	-0.59	4.13	7.93E-13	5.28E-12
CYBB	-0.59	4.16	3.60E-12	2.24E-11
C9orf47	-0.59	0.49	7.14E-16	6.28E-15
GNAO1	-0.59	2.68	3.68E-12	2.29E-11
HIVEP3	-0.59	2.11	4.06E-27	8.94E-26
DNA2	-0.59	1.48	1.14E-11	6.74E-11
SEC14L2	-0.59	0.18	1.87E-12	1.20E-11
RGAG1	-0.59	0.26	1.63E-13	1.15E-12
MMP11	-0.59	1.87	5.91E-12	3.59E-11
SUSD4	-0.59	2.03	3.84E-09	1.75E-08
B3GALT1	-0.59	-1.76	2.37E-11	1.36E-10
PHYHD1	-0.59	3.60	2.19E-21	3.14E-20
REV1	-0.59	5.59	1.02E-15	8.89E-15
PHC1P1	-0.59	-0.38	0.004781624	0.00901533
SLC29A2	-0.60	3.84	7.69E-22	1.13E-20
STRBP	-0.60	3.92	4.56E-36	1.93E-34
TWIST2	-0.60	0.06	4.42E-08	1.77E-07
ST3GAL1	-0.60	7.29	5.81E-24	1.01E-22
CTH	-0.60	0.72	1.02E-16	9.72E-16
HPCAL4	-0.60	-0.10	5.06E-07	1.76E-06
FGF7	-0.60	4.46	1.59E-14	1.25E-13
HCLS1	-0.60	4.54	2.58E-18	2.82E-17
ACPP	-0.60	-1.51	6.10E-10	3.02E-09
SULT4A1	-0.60	-2.56	1.11E-07	4.19E-07
TLR4	-0.60	4.00	1.12E-12	7.38E-12
BCL2L1	-0.60	5.66	3.97E-41	2.42E-39
XRCC4	-0.60	3.04	1.98E-13	1.39E-12
KCNH4	-0.60	-2.09	3.23E-10	1.65E-09
DDX21	-0.60	5.31	3.84E-12	2.38E-11
RP11-61N20.3	-0.60	-1.92	0.004211874	0.008041593
TAF4B	-0.60	1.01	2.15E-10	1.12E-09
BIRC3	-0.61	3.46	3.99E-13	2.72E-12
MARVELD2	-0.61	0.08	2.21E-18	2.43E-17
TEAD1	-0.61	6.67	1.18E-13	8.42E-13
IFI30	-0.61	3.37	9.63E-09	4.17E-08
DPP3P2	-0.61	0.85	0.004030704	0.007728285
EGLN1	-0.61	6.84	8.82E-25	1.64E-23
XK	-0.61	3.79	2.54E-14	1.95E-13
MTUS2	-0.61	6.13	1.44E-25	2.84E-24
HTATIP2	-0.61	3.50	1.90E-33	6.62E-32
DAAM2	-0.61	4.88	2.47E-12	1.56E-11
BEND2	-0.61	-2.05	2.72E-10	1.40E-09
CALCB	-0.61	-2.61	1.60E-07	5.93E-07
KIAA0040	-0.61	4.68	1.20E-24	2.20E-23
PAPSS2	-0.62	3.83	7.65E-16	6.72E-15
ATP1B1	-0.62	7.70	7.55E-34	2.73E-32
LPAR3	-0.62	2.88	6.05E-13	4.07E-12
NCAPH	-0.62	-1.66	2.63E-10	1.35E-09
LDHA	-0.62	7.98	2.77E-24	4.90E-23
SLC15A2	-0.62	2.56	4.02E-13	2.74E-12
CHRDL2	-0.62	0.69	3.56E-05	9.62E-05
FAM222A	-0.62	2.09	5.02E-12	3.08E-11
PPTC7	-0.62	6.88	2.12E-22	3.30E-21
ARMC12	-0.62	-1.88	2.23E-09	1.04E-08
IL1R1	-0.62	5.51	1.94E-14	1.50E-13
PIP5K1B	-0.62	5.25	1.56E-17	1.61E-16

FAM84A	-0.62	-0.10	4.21E-09	1.91E-08
MAN1A2P1	-0.62	-1.39	3.05E-05	8.32E-05
DDN	-0.62	-0.55	1.96E-10	1.02E-09
PDK4	-0.62	9.72	7.28E-05	0.00018788
CCDC69	-0.62	7.00	3.43E-47	3.14E-45
SRGAP2D	-0.62	-1.88	0.000963132	0.002064029
TCN2	-0.62	4.08	9.25E-23	1.48E-21
RBMS1P1	-0.62	-2.51	2.58E-05	7.12E-05
GCLM	-0.62	3.58	5.34E-17	5.26E-16
SLC17A9	-0.63	0.14	1.16E-09	5.58E-09
SPARCL1	-0.63	9.80	2.21E-24	3.96E-23
ECE2	-0.63	0.26	4.70E-15	3.87E-14
PTPRG	-0.63	5.64	3.11E-16	2.84E-15
SIGLEC10	-0.63	0.83	6.41E-13	4.30E-12
RP4-539M6.19	-0.63	-1.72	0.001153229	0.002443281
CORIN	-0.63	5.58	5.49E-08	2.17E-07
PREX1	-0.63	4.11	9.99E-23	1.59E-21
ABCC2	-0.63	-1.23	5.10E-10	2.54E-09
IL17RB	-0.63	1.33	1.15E-19	1.42E-18
CD53	-0.63	3.38	5.18E-15	4.25E-14
GREB1L	-0.63	3.43	2.39E-17	2.42E-16
CYP1A1	-0.63	-1.77	0.000330848	0.00076936
MAMDC2	-0.63	4.33	1.22E-10	6.53E-10
PRDX6	-0.63	7.93	1.52E-33	5.35E-32
ADGRF2	-0.63	-1.23	8.93E-05	0.000227849
WNT6	-0.64	-1.94	1.97E-05	5.54E-05
TMEM52	-0.64	-1.95	2.66E-08	1.09E-07
PAX9	-0.64	-2.61	8.12E-10	3.98E-09
HEY2	-0.64	4.36	3.76E-20	4.81E-19
CATSPERB	-0.64	-0.60	4.34E-15	3.59E-14
CACNA2D3	-0.64	4.28	7.02E-24	1.21E-22
MS4A4A	-0.64	3.59	3.68E-13	2.51E-12
TTK	-0.64	-2.10	3.84E-07	1.35E-06
CDC45	-0.64	-2.19	2.01E-08	8.40E-08
ENPP4	-0.64	5.10	6.18E-14	4.57E-13
BBS12	-0.64	2.77	5.05E-18	5.40E-17
WNT5A	-0.64	1.45	5.48E-14	4.08E-13
MAP2K6	-0.64	0.15	5.03E-13	3.40E-12
TBX20	-0.64	2.09	0.000908706	0.001956153
CALCOCO2	-0.64	7.54	5.07E-55	8.41E-53
KCNJ1	-0.64	-2.48	7.37E-11	4.03E-10
RASL10B	-0.64	3.47	1.77E-21	2.56E-20
TPST2	-0.64	4.61	4.26E-43	3.00E-41
HMOX1	-0.65	2.58	1.52E-07	5.68E-07
RNF144B	-0.65	4.23	6.36E-31	1.83E-29
MMRN1	-0.65	1.83	2.01E-05	5.63E-05
AZGP1	-0.65	5.99	2.98E-15	2.49E-14
AURKB	-0.65	-2.27	1.07E-07	4.06E-07
FAM131C	-0.65	0.51	1.86E-09	8.74E-09
FLT3	-0.65	-0.95	7.47E-08	2.90E-07
GPD1L	-0.65	8.14	1.24E-24	2.27E-23
TLCD2	-0.65	2.17	6.15E-24	1.06E-22
RP11-385D13.1	-0.65	-1.96	4.42E-07	1.55E-06
CCNG1	-0.65	5.95	2.26E-26	4.69E-25
AC013264.1	-0.65	-0.78	0.002515564	0.005016752
EBLN2	-0.65	-1.04	1.89E-05	5.33E-05
GADD45B	-0.65	4.60	2.74E-13	1.90E-12
SLC1A3	-0.65	6.62	7.21E-17	7.01E-16
NRCAM	-0.65	-1.16	2.99E-08	1.22E-07
CTD-3138B18.4	-0.65	-1.84	0.000737851	0.001618754
TIMP3	-0.66	9.00	1.10E-29	2.93E-28
GPR12	-0.66	-1.79	3.34E-09	1.53E-08
ADH1A	-0.66	-1.50	9.15E-10	4.45E-09
FGR	-0.66	2.62	2.35E-17	2.39E-16
USP31	-0.66	5.42	1.04E-32	3.36E-31
ATP1A1	-0.66	8.08	1.17E-31	3.49E-30
HRCT1	-0.66	0.94	1.34E-07	5.02E-07
TGFA	-0.66	0.00	1.34E-09	6.37E-09
TIFA	-0.66	3.04	6.81E-22	1.01E-20
PCDHGC4	-0.66	0.47	0.000233907	0.000557343
PIK3C2A	-0.66	5.43	2.58E-11	1.47E-10
CPO	-0.67	-1.70	9.68E-12	5.77E-11
KCNJ11	-0.67	2.85	3.82E-19	4.49E-18
SLC7A8	-0.67	3.45	1.06E-18	1.20E-17
ELOVL7	-0.67	0.37	4.90E-09	2.20E-08
GPSM2	-0.67	2.29	4.32E-19	5.05E-18
DOCK9	-0.67	5.87	2.97E-15	2.48E-14
FREM2	-0.67	-1.55	1.11E-07	4.21E-07
ARG2	-0.67	1.22	1.70E-09	8.02E-09
KLF15	-0.67	4.62	2.62E-17	2.65E-16
SMYD1	-0.67	8.45	2.67E-33	9.13E-32
EYA4	-0.67	4.15	7.30E-14	5.34E-13
UHRF1	-0.68	-0.81	4.21E-11	2.36E-10

HOGA1	-0.68	3.99	9.66E-21	1.30E-19
RP11-651P23.4	-0.68	-1.24	4.41E-08	1.76E-07
PAX8	-0.68	-0.07	9.91E-08	3.78E-07
QSOX1	-0.68	6.25	3.54E-28	8.42E-27
FERP1	-0.68	-2.87	8.35E-05	0.000213641
ZNF682	-0.68	2.96	5.05E-29	1.27E-27
MRO	-0.68	1.37	2.32E-17	2.36E-16
PRRG4	-0.68	0.98	2.19E-12	1.39E-11
RP11-561B11.2	-0.68	0.15	1.56E-05	4.47E-05
MID1IP1	-0.68	3.59	9.39E-25	1.74E-23
SGO1	-0.68	-2.12	8.56E-11	4.63E-10
CPNE4	-0.68	4.04	8.64E-09	3.76E-08
BCL2L2-PABPN1	-0.69	-0.31	0.003209413	0.006268386
IGSF21	-0.69	-0.90	2.81E-10	1.44E-09
SLC38A2	-0.69	7.44	1.06E-18	1.20E-17
VWA5B1	-0.69	-2.18	5.60E-10	2.78E-09
VPS13D	-0.69	6.90	2.55E-32	8.03E-31
ALPL	-0.69	3.33	3.75E-22	5.69E-21
C1QB	-0.69	5.27	1.38E-10	7.34E-10
TMED10P2	-0.69	-2.35	1.34E-09	6.38E-09
OSBPL11	-0.69	4.04	5.17E-23	8.39E-22
PPP1R1A	-0.69	5.31	4.74E-16	4.23E-15
MERTK	-0.69	3.49	1.02E-20	1.37E-19
GFPT2	-0.69	3.43	1.03E-17	1.08E-16
PPIP5K2	-0.69	5.20	8.60E-19	9.84E-18
LONRF3	-0.69	-0.01	2.78E-11	1.59E-10
SPECC1L-ADORA2A	-0.69	-0.83	0.000707809	0.001555472
PIM1	-0.69	3.73	3.18E-16	2.90E-15
SLC30A10	-0.69	-1.59	7.31E-12	4.41E-11
CCL2	-0.69	3.44	6.43E-07	2.20E-06
HPSE	-0.70	0.31	3.89E-16	3.51E-15
BMPER	-0.70	0.04	5.99E-11	3.31E-10
KDR	-0.70	5.33	2.12E-16	1.97E-15
LILRA6	-0.70	1.28	8.87E-15	7.13E-14
FAM58A	-0.70	4.11	1.06E-24	1.95E-23
SCN3A	-0.70	1.90	7.86E-11	4.28E-10
CHI3L2	-0.70	0.78	4.59E-06	1.41E-05
MICALCL	-0.70	-2.31	4.54E-08	1.81E-07
SULT1B1	-0.70	2.45	4.35E-15	3.60E-14
P2RY12	-0.70	-0.44	4.69E-07	1.63E-06
NCEH1	-0.70	5.41	3.08E-23	5.08E-22
SEMA4B	-0.70	3.88	2.42E-27	5.44E-26
FAM107A	-0.71	6.69	2.83E-34	1.05E-32
KLHL41	-0.71	7.17	4.95E-30	1.35E-28
SLC36A1	-0.71	2.43	2.02E-24	3.63E-23
ANXA3	-0.71	5.23	2.21E-32	6.98E-31
ADAM9	-0.71	6.55	2.14E-15	1.81E-14
ATP8B5P	-0.71	-1.39	5.30E-16	4.72E-15
PLTP	-0.71	5.31	1.19E-13	8.49E-13
RP5-937E21.8	-0.71	-2.45	6.41E-06	1.93E-05
ECT2	-0.71	2.34	6.19E-14	4.58E-13
TK1	-0.71	-0.27	1.74E-11	1.01E-10
SPP1	-0.71	3.21	2.78E-07	1.00E-06
KCNA7	-0.71	-0.36	1.25E-11	7.35E-11
MTFP1	-0.71	2.80	4.41E-12	2.72E-11
ADCK3	-0.71	7.61	3.75E-33	1.26E-31
CCDC68	-0.72	3.46	3.78E-12	2.35E-11
SLC52A3	-0.72	1.94	1.18E-23	2.01E-22
RP11-100N21.1	-0.72	-1.87	7.23E-09	3.19E-08
TGFBR2	-0.72	7.61	5.38E-27	1.17E-25
PTDSS1	-0.72	6.82	9.88E-47	8.81E-45
CALCRL	-0.72	4.84	5.22E-11	2.90E-10
CD5L	-0.72	-1.71	3.15E-07	1.13E-06
LSAMP	-0.72	2.84	2.03E-13	1.42E-12
GGH	-0.72	1.22	1.80E-19	2.17E-18
TMEM100	-0.72	2.73	1.02E-12	6.73E-12
SLC19A2	-0.72	5.19	8.67E-13	5.75E-12
SPC24	-0.72	0.84	1.60E-29	4.18E-28
SLC4A7	-0.73	4.06	4.07E-10	2.06E-09
MEDAG	-0.73	3.27	1.29E-15	1.11E-14
RASD1	-0.73	3.39	3.40E-05	9.21E-05
PLCXD3	-0.73	7.23	1.84E-27	4.18E-26
HPR	-0.73	6.29	4.74E-09	2.13E-08
GRAP2	-0.73	0.11	3.21E-14	2.43E-13
RP11-697N18.3	-0.73	-2.51	5.38E-10	2.67E-09
EPB41L4B	-0.73	3.90	3.74E-20	4.79E-19
MAP3K8	-0.73	2.95	4.89E-20	6.22E-19
FMO5	-0.73	2.15	2.11E-17	2.16E-16
SCN10A	-0.73	-1.51	4.04E-10	2.04E-09
C1QL1	-0.73	-2.01	9.03E-08	3.47E-07
LRRC8E	-0.73	-0.72	1.18E-14	9.35E-14
PVR	-0.73	4.93	1.03E-35	4.21E-34

CRISPLD2	-0.73	5.29	9.99E-25	1.84E-23
MANEA	-0.73	3.23	1.20E-11	7.06E-11
CADPS2	-0.73	3.88	2.21E-30	6.14E-29
ELL2	-0.74	5.61	1.83E-18	2.03E-17
NCAPG	-0.74	-0.71	1.34E-10	7.12E-10
MLXIPL	-0.74	0.75	1.80E-06	5.81E-06
BANK1	-0.74	-1.49	3.31E-08	1.35E-07
HK2	-0.74	4.10	6.36E-17	6.22E-16
GPR4	-0.74	2.72	2.75E-30	7.59E-29
ARNTL	-0.74	3.65	1.70E-14	1.33E-13
SIRPB2	-0.74	1.10	2.26E-12	1.44E-11
SSTR2	-0.74	1.90	3.97E-36	1.69E-34
AC084219.2	-0.75	-0.87	0.000558995	0.001253603
MYO7B	-0.75	3.07	1.15E-12	7.59E-12
KIF5C	-0.75	0.61	2.29E-16	2.12E-15
C10orf11	-0.75	2.67	1.45E-24	2.63E-23
GPRC5A	-0.75	0.85	8.46E-15	6.82E-14
GPAT2	-0.75	2.75	1.76E-18	1.96E-17
NUDT4	-0.75	8.27	6.47E-40	3.66E-38
C3	-0.76	7.15	6.00E-16	5.31E-15
TUBA1C	-0.76	4.75	3.80E-18	4.10E-17
STAT3	-0.76	6.93	3.72E-33	1.25E-31
FAM209B	-0.76	-2.42	1.94E-12	1.24E-11
ADAM11	-0.76	2.39	2.66E-12	1.68E-11
C8orf88	-0.76	4.20	9.37E-42	5.97E-40
TGFBR3	-0.76	5.34	1.42E-28	3.45E-27
C7orf55-LUC7L2	-0.76	2.03	0.013973653	0.023861625
RP11-3B12.4	-0.76	-2.82	6.68E-08	2.62E-07
BCAT1	-0.76	3.00	2.06E-17	2.10E-16
SLC1A1	-0.77	2.10	1.73E-25	3.39E-24
TBC1D29	-0.77	-1.73	4.49E-13	3.05E-12
MYBL2	-0.77	-2.20	9.35E-08	3.58E-07
RGS9BP	-0.77	-0.04	3.36E-19	3.98E-18
GPR183	-0.77	1.42	2.02E-11	1.17E-10
APOB	-0.77	4.81	1.30E-17	1.36E-16
SOCS3	-0.78	3.42	4.50E-09	2.03E-08
IL15RA	-0.78	2.42	9.68E-38	4.70E-36
GPX3	-0.78	9.33	3.01E-48	3.17E-46
AMD1	-0.78	6.32	3.03E-35	1.20E-33
LCN15	-0.78	-0.58	9.16E-21	1.24E-19
RGR	-0.78	-1.07	5.72E-15	4.67E-14
MKI67	-0.78	0.10	2.03E-07	7.46E-07
AKR1C1	-0.78	4.84	9.54E-20	1.19E-18
AIF1L	-0.79	5.41	1.24E-43	9.18E-42
SLC7A11	-0.79	-0.71	3.54E-14	2.67E-13
ARRDC4	-0.79	4.45	3.23E-20	4.17E-19
HIF3A	-0.80	5.95	3.12E-28	7.48E-27
CNR1	-0.80	0.16	1.41E-09	6.69E-09
EDN1	-0.80	2.30	5.43E-17	5.34E-16
HAS2	-0.80	1.43	2.96E-12	1.86E-11
MINOS1-NBL1	-0.80	-0.34	0.000462417	0.001051803
HK3	-0.80	1.22	4.61E-16	4.13E-15
RPGR	-0.80	3.58	5.17E-26	1.05E-24
EPN3	-0.80	2.03	8.37E-15	6.75E-14
FAM78B	-0.80	1.89	2.60E-44	1.98E-42
AC129778.2	-0.81	0.29	5.69E-20	7.21E-19
LPCAT3	-0.81	4.55	9.70E-37	4.28E-35
FAM83F	-0.81	1.97	5.50E-29	1.37E-27
ADARB2	-0.81	-1.71	6.28E-16	5.55E-15
SLC31A2	-0.81	3.40	9.52E-42	6.04E-40
TMEM132C	-0.81	1.17	3.07E-13	2.11E-12
CHI3L1	-0.81	-0.11	3.34E-06	1.04E-05
SLC36A4	-0.81	3.04	1.95E-33	6.76E-32
MT1M	-0.82	0.93	6.58E-08	2.58E-07
WASF1	-0.82	0.65	9.64E-21	1.30E-19
ANLN	-0.82	0.09	1.79E-08	7.53E-08
SLC38A4	-0.82	-1.99	1.34E-09	6.39E-09
MARK3	-0.82	6.46	1.94E-74	9.84E-72
SIGLEC14	-0.82	-0.40	2.09E-09	9.82E-09
COL25A1	-0.82	-0.62	4.48E-09	2.02E-08
LAPTM5	-0.82	4.87	7.23E-21	9.88E-20
PHACTR3	-0.83	1.54	6.70E-13	4.48E-12
TFRC	-0.83	6.43	5.27E-20	6.70E-19
WNT9B	-0.83	-1.28	3.22E-16	2.93E-15
SERPINB8	-0.83	2.05	6.29E-25	1.18E-23
SLC2A1	-0.83	4.36	5.92E-39	3.12E-37
GATSL2	-0.83	-0.38	4.40E-11	2.46E-10
GGT5	-0.83	4.34	7.66E-20	9.63E-19
ADGRF5	-0.83	6.89	6.70E-33	2.21E-31
KCNK3	-0.83	0.99	1.86E-19	2.24E-18
TTLL6	-0.84	-2.35	1.05E-11	6.24E-11
KLF10	-0.84	5.09	8.20E-24	1.41E-22
MCM10	-0.84	-1.76	5.88E-16	5.22E-15

OLAH	-0.84	-1.48	1.05E-21	1.54E-20
C1orf162	-0.84	3.38	2.34E-30	6.51E-29
GLUL	-0.84	8.32	2.61E-34	9.73E-33
CLEC7A	-0.84	2.07	3.27E-17	3.28E-16
GABRR2	-0.84	-1.49	9.02E-08	3.46E-07
RP11-211G3.2	-0.85	-1.30	2.77E-19	3.30E-18
STON1-GTF2A1L	-0.85	-2.57	1.49E-05	4.27E-05
LGR6	-0.85	0.85	3.52E-21	4.95E-20
PAQR5	-0.85	0.53	2.53E-22	3.91E-21
RP11-381O7.3	-0.85	2.20	5.40E-26	1.09E-24
ABCB1	-0.85	2.51	8.55E-20	1.07E-18
FAM3C2	-0.85	-1.15	0.003018889	0.005926674
FAM212B	-0.85	5.10	1.39E-52	1.92E-50
FCER1G	-0.85	3.63	1.08E-26	2.29E-25
PPM1E	-0.85	0.47	9.28E-12	5.54E-11
PTX3	-0.86	1.57	5.58E-07	1.93E-06
SAMSN1	-0.86	1.25	7.47E-13	4.98E-12
FAM179A	-0.86	3.34	1.13E-15	9.79E-15
ETNK2	-0.86	1.43	2.04E-28	4.94E-27
TOP2A	-0.86	0.18	2.62E-08	1.08E-07
AL022067.1	-0.86	-1.12	7.31E-05	0.00018852
VIT	-0.87	3.86	5.19E-27	1.13E-25
PKHD1L1	-0.87	1.13	5.31E-08	2.10E-07
ZBED6	-0.87	0.60	0.00029615	0.000695139
SLC5A1	-0.87	7.46	1.69E-29	4.40E-28
MSR1	-0.87	3.30	5.23E-19	6.07E-18
GALNT4	-0.87	1.29	7.69E-05	0.000197585
CDRT1	-0.88	-1.09	6.64E-22	9.87E-21
STEAP4	-0.88	6.02	5.33E-28	1.25E-26
SIGLEC7	-0.88	-0.69	8.91E-19	1.02E-17
SENP3-EIF4A1	-0.88	-2.92	5.67E-06	1.72E-05
TXNRD1	-0.88	6.77	2.78E-30	7.65E-29
EHF	-0.88	-2.09	4.71E-11	2.63E-10
MBNL3	-0.88	3.49	2.74E-12	1.73E-11
TFEC	-0.89	1.23	1.33E-13	9.47E-13
PCK1	-0.89	-0.50	0.000118207	0.000295342
THBS1	-0.89	6.54	6.72E-15	5.44E-14
PDE11A	-0.89	0.82	8.30E-19	9.50E-18
HP	-0.89	2.54	4.84E-10	2.41E-09
PCNT	-0.89	5.65	1.96E-43	1.41E-41
RP11-236F9.2	-0.89	0.33	2.74E-22	4.22E-21
LRRN3	-0.89	1.56	7.94E-14	5.79E-13
SAMHD1	-0.89	5.72	3.59E-36	1.54E-34
ADAMTS5	-0.89	3.63	9.18E-19	1.05E-17
ALOX5AP	-0.90	2.73	2.62E-25	5.02E-24
ADGRD1	-0.90	5.26	1.36E-43	9.83E-42
KIAA1549	-0.90	2.33	5.69E-35	2.20E-33
SELE	-0.90	1.00	2.61E-06	8.27E-06
BCL6	-0.90	5.68	1.08E-53	1.64E-51
ZNF366	-0.90	1.44	4.55E-09	2.05E-08
CLEC4G	-0.90	-1.21	5.83E-09	2.60E-08
GCOM1	-0.91	3.81	2.66E-10	1.37E-09
TLR2	-0.91	2.86	4.99E-23	8.12E-22
SLA	-0.91	3.04	1.05E-19	1.30E-18
CBSL	-0.91	-0.37	0.002300425	0.00462404
TATDN2P2	-0.91	-2.19	2.26E-11	1.30E-10
STEAP3	-0.91	3.19	5.48E-50	6.38E-48
DEPDC1B	-0.91	-2.64	6.46E-15	5.25E-14
F8	-0.91	6.52	2.36E-29	6.11E-28
RNF157	-0.91	2.49	8.20E-30	2.20E-28
FOSL1	-0.91	-0.40	2.65E-09	1.23E-08
FP325317.1	-0.91	3.77	2.77E-33	9.46E-32
CTD-2331H12.8	-0.91	-2.12	0.000189889	0.000459034
SLC25A18	-0.91	1.74	5.16E-27	1.12E-25
C1QTNF1	-0.92	5.81	1.25E-19	1.54E-18
TMED7-TICAM2	-0.92	-1.31	7.19E-07	2.45E-06
CSDC2	-0.92	7.06	3.06E-24	5.40E-23
S100A8	-0.92	3.34	4.34E-11	2.43E-10
TMEM252	-0.92	-0.67	6.92E-08	2.70E-07
CR1	-0.92	0.93	2.51E-12	1.59E-11
MPP3	-0.92	5.26	4.94E-33	1.64E-31
STOX1	-0.93	-0.98	4.19E-17	4.16E-16
ADAMTS15	-0.93	4.28	6.06E-38	3.01E-36
GPR17	-0.93	2.82	2.47E-12	1.57E-11
C11orf52	-0.93	0.32	3.58E-27	7.92E-26
CD109	-0.93	4.31	6.24E-36	2.60E-34
RP11-460N11.2	-0.93	-1.84	4.42E-10	2.22E-09
HMOX2	-0.93	5.83	1.26E-69	4.81E-67
ATP2A2	-0.93	10.69	3.13E-43	2.21E-41
ITPKA	-0.93	-0.49	1.58E-13	1.12E-12
TPO	-0.93	2.78	3.10E-32	9.68E-31
ALOX5	-0.93	2.23	9.65E-31	2.74E-29
ADGRF4	-0.93	-1.11	3.61E-08	1.46E-07

OSMR	-0.94	4.63	1.23E-29	3.25E-28
ANGPTL4	-0.94	2.42	2.66E-08	1.10E-07
NID1	-0.94	6.65	1.62E-41	1.00E-39
TRPC4	-0.95	-0.52	1.30E-18	1.46E-17
TUBAL3	-0.95	-1.98	9.18E-15	7.37E-14
WWC1	-0.95	-1.01	9.38E-14	6.79E-13
TC2N	-0.95	2.55	4.74E-20	6.04E-19
CBS	-0.95	1.80	1.99E-11	1.15E-10
RBM22P3	-0.96	-2.21	6.14E-08	2.42E-07
NECTIN1	-0.96	3.16	1.79E-40	1.06E-38
GXYLT1P6	-0.97	-2.40	3.54E-07	1.26E-06
KLHL32	-0.97	-0.87	9.70E-18	1.02E-16
CTB-50L17.14	-0.97	1.64	3.98E-14	2.99E-13
DNER	-0.97	0.04	1.90E-14	1.48E-13
CCR1	-0.98	1.85	1.66E-23	2.80E-22
GRB7	-0.98	-1.46	4.79E-21	6.63E-20
PPL	-0.98	2.88	1.35E-43	9.83E-42
MELK	-0.98	-2.06	2.19E-13	1.52E-12
F5	-0.98	2.80	4.57E-11	2.55E-10
GRB14	-0.98	3.47	1.25E-46	1.10E-44
EIF4EBP1	-0.98	3.16	6.21E-25	1.17E-23
RP11-244H3.4	-0.99	0.06	3.02E-07	1.08E-06
SLCO2A1	-0.99	4.19	1.66E-29	4.33E-28
ANGPT4	-0.99	-1.59	9.49E-15	7.60E-14
SYT13	-0.99	2.37	1.24E-20	1.66E-19
HS6ST2	-0.99	-1.32	2.13E-12	1.36E-11
KCND3	-0.99	4.85	1.01E-53	1.53E-51
AGTR1	-0.99	1.73	9.82E-23	1.56E-21
KCNK1	-0.99	3.34	2.29E-31	6.73E-30
RP11-544M22.13	-1.00	-1.72	4.07E-05	0.000109146
AC006116.27	-1.00	-1.79	2.21E-05	6.16E-05
FMN1	-1.00	1.85	3.18E-24	5.60E-23
HERC2P3	-1.00	3.10	2.95E-13	2.04E-12
NAMPT	-1.01	7.20	1.36E-31	4.04E-30
MATN3	-1.01	-0.95	7.14E-19	8.22E-18
RP11-15E1.6	-1.01	-2.56	2.90E-12	1.83E-11
MOG	-1.02	0.96	4.85E-14	3.62E-13
RP3-509I19.1	-1.02	-0.76	2.71E-07	9.79E-07
PRODH	-1.03	2.42	4.99E-18	5.34E-17
C15orf59	-1.03	4.67	9.95E-43	6.89E-41
CPAMD8	-1.03	4.18	6.18E-38	3.06E-36
AQP7P5	-1.03	-2.09	4.09E-07	1.44E-06
AASS	-1.04	4.69	8.02E-22	1.18E-20
SMTNL2	-1.04	2.68	1.06E-37	5.05E-36
AC008132.13	-1.04	-1.51	1.15E-24	2.11E-23
FPR1	-1.04	2.37	6.51E-23	1.05E-21
PALLD	-1.05	9.29	2.16E-54	3.38E-52
SBK3	-1.05	0.34	6.55E-14	4.82E-13
CPLX3	-1.06	1.50	1.04E-10	5.61E-10
CAMSAP3	-1.06	-2.24	1.30E-15	1.12E-14
LMAN1L	-1.06	1.56	1.81E-12	1.16E-11
KNG1	-1.06	-2.68	8.91E-19	1.02E-17
MRC1	-1.06	5.14	9.24E-28	2.14E-26
SPOCK3	-1.07	-1.98	4.19E-09	1.90E-08
KRT7	-1.07	-2.08	9.83E-10	4.75E-09
ART3	-1.07	5.93	1.58E-47	1.54E-45
BZW1P2	-1.09	-0.40	0.000114326	0.000286371
SERPINF2	-1.09	0.11	4.16E-21	5.81E-20
IL20RA	-1.09	1.24	1.04E-18	1.19E-17
ZBTB16	-1.09	4.01	3.15E-20	4.08E-19
CEP55	-1.09	-1.44	8.50E-15	6.84E-14
TFCP2L1	-1.09	-0.77	9.02E-24	1.55E-22
GXYLT1P4	-1.10	-1.42	3.14E-14	2.39E-13
ADAMTS9	-1.10	4.83	1.28E-43	9.38E-42
ZC3H11B	-1.11	-1.22	1.28E-14	1.02E-13
F13A1	-1.11	6.29	4.93E-28	1.17E-26
ZFP57	-1.11	-0.90	0.003164863	0.00619148
MEI4	-1.12	-1.20	2.87E-19	3.41E-18
PCSK1	-1.12	-1.32	1.32E-18	1.49E-17
VSTM2L	-1.13	2.16	1.26E-16	1.20E-15
RP11-6L6.2	-1.13	-0.96	2.89E-29	7.42E-28
TIMP4	-1.13	4.33	1.32E-32	4.25E-31
CFAP77	-1.14	-0.18	5.85E-38	2.92E-36
NFXL1	-1.14	3.08	2.03E-35	8.13E-34
MT1X	-1.14	3.96	2.58E-17	2.61E-16
OPN4	-1.14	-0.88	7.18E-19	8.26E-18
PLIN2	-1.15	6.32	1.26E-42	8.63E-41
CACNA1E	-1.16	-1.34	6.81E-18	7.24E-17
TMTC1	-1.16	6.12	1.70E-50	2.04E-48
TMEM178B	-1.16	5.70	5.13E-37	2.32E-35
MYOT	-1.16	4.12	2.08E-28	5.04E-27
HS6ST3	-1.16	0.96	4.38E-11	2.45E-10
S100A3	-1.17	-1.78	1.53E-19	1.86E-18

RHAG	-1.17	-1.40	8.23E-20	1.03E-18
BIVM-ERCC5	-1.18	1.76	0.000443223	0.001011579
TMEM63C	-1.18	-0.97	2.33E-13	1.62E-12
TMEM151B	-1.18	-1.58	2.12E-11	1.22E-10
NSG1	-1.19	1.48	2.31E-34	8.65E-33
CHAC2	-1.19	0.84	2.16E-40	1.26E-38
ST6GALNAC3	-1.19	2.94	5.31E-56	9.77E-54
FAM46B	-1.20	3.67	2.58E-34	9.61E-33
CPM	-1.21	3.71	4.82E-35	1.87E-33
CHL1	-1.21	2.51	6.40E-23	1.03E-21
NQO1	-1.21	3.72	1.26E-43	9.28E-42
AZGP1P1	-1.22	-1.23	1.34E-19	1.64E-18
KCNIP2	-1.24	6.44	9.82E-26	1.96E-24
S1PR3	-1.24	4.63	1.85E-64	5.65E-62
SHISA3	-1.24	2.92	2.95E-27	6.58E-26
TMEM132B	-1.25	0.71	1.06E-31	3.17E-30
RP11-438J1.1	-1.25	-1.31	3.50E-07	1.24E-06
CFTR	-1.26	-1.94	1.32E-24	2.41E-23
OVCH1	-1.26	-2.06	1.99E-23	3.34E-22
EDNRB	-1.26	5.36	2.26E-41	1.39E-39
FCGR3A	-1.26	3.53	2.21E-32	7.00E-31
POM121L9P	-1.27	0.51	6.48E-48	6.61E-46
EREG	-1.27	-0.01	1.13E-17	1.18E-16
AC007325.2	-1.29	2.75	9.82E-16	8.54E-15
CD38	-1.29	2.80	6.12E-46	5.18E-44
NUDT4P1	-1.29	1.14	3.83E-09	1.75E-08
NUDT4P2	-1.29	1.14	3.83E-09	1.75E-08
SERPINE1	-1.29	4.33	8.54E-14	6.22E-13
GRIP1	-1.29	-1.34	1.10E-19	1.36E-18
NAMPTP1	-1.29	0.85	2.74E-08	1.13E-07
CA14	-1.29	1.73	2.11E-33	7.28E-32
SCGB1D2	-1.30	-2.19	6.23E-20	7.87E-19
PQLC2L	-1.30	0.08	3.95E-38	2.00E-36
SERTM1	-1.31	-1.14	9.00E-20	1.12E-18
ANPEP	-1.32	2.14	5.33E-35	2.07E-33
CHST9	-1.32	2.24	3.52E-20	4.52E-19
SERPINA5	-1.33	1.10	2.09E-32	6.63E-31
STAC2	-1.33	-1.42	2.65E-33	9.09E-32
RP11-1081M5.3	-1.34	0.64	1.57E-19	1.91E-18
IL18R1	-1.35	2.35	7.03E-42	4.49E-40
TUBB4A	-1.36	-1.06	7.77E-33	2.54E-31
FER1L6	-1.36	-2.54	1.51E-17	1.56E-16
SSTR5	-1.37	-1.21	7.78E-12	4.67E-11
CHDH	-1.37	1.89	1.28E-61	3.06E-59
NPPC	-1.37	-1.94	1.95E-19	2.35E-18
HOOK1	-1.37	1.67	3.43E-39	1.82E-37
MT1A	-1.38	-0.45	9.43E-13	6.25E-12
TDRD9	-1.38	1.40	4.56E-49	5.03E-47
PLA2G4F	-1.39	-0.12	3.40E-29	8.64E-28
SLC11A1	-1.39	2.59	3.55E-37	1.64E-35
ANKRD2	-1.40	6.98	6.43E-26	1.30E-24
BMP7	-1.40	1.08	4.47E-25	8.47E-24
ADORA3	-1.41	0.96	1.07E-34	4.11E-33
S100A9	-1.42	4.15	1.86E-25	3.64E-24
OVOS2	-1.43	-0.81	1.29E-19	1.58E-18
ADH1B	-1.45	6.49	3.57E-35	1.40E-33
FAM155B	-1.46	4.38	5.49E-27	1.19E-25
WNK3	-1.49	0.41	3.25E-47	2.99E-45
SIGLEC9	-1.51	-0.01	4.47E-56	8.32E-54
AQP3	-1.51	3.00	5.53E-48	5.75E-46
AREG	-1.53	1.53	3.74E-22	5.67E-21
TMIGD3	-1.53	-0.37	1.24E-34	4.76E-33
C3orf36	-1.55	-2.40	7.09E-33	2.34E-31
CNTN3	-1.57	1.36	1.02E-27	2.35E-26
ETNPPL	-1.57	2.08	1.99E-35	8.00E-34
MGST1	-1.59	4.16	1.25E-36	5.50E-35
RHBDL3	-1.60	-0.39	1.14E-29	3.02E-28
RP11-443P15.2	-1.63	-1.76	4.36E-36	1.85E-34
ADAMTS4	-1.64	3.78	1.77E-39	9.67E-38
SYN2	-1.64	1.56	1.34E-39	7.41E-38
MARCO	-1.65	1.62	5.18E-17	5.11E-16
IL10	-1.68	-1.08	9.02E-40	5.05E-38
LGI3	-1.73	-2.23	9.54E-32	2.86E-30
LAD1	-1.74	1.96	3.21E-46	2.74E-44
TCF24	-1.76	-0.48	3.45E-27	7.65E-26
LYVE1	-1.77	5.26	3.78E-59	8.23E-57
PPEF1	-1.77	-2.04	2.22E-38	1.14E-36
GMNC	-1.82	-1.84	8.81E-34	3.17E-32
SAA2	-1.84	-1.60	6.81E-21	9.32E-20
HMGCS2	-1.85	3.28	1.92E-17	1.96E-16
BLM	-1.85	2.75	4.45E-77	2.76E-74
FGF10	-1.85	-2.08	5.32E-30	1.44E-28
VSIG4	-1.86	4.84	1.49E-70	6.25E-68

RARRES1	-1.86	2.01	2.61E-38	1.33E-36
METTL7B	-1.86	2.65	1.77E-49	1.97E-47
FCGBP	-1.90	1.95	1.80E-31	5.30E-30
GALNT15	-1.93	4.13	8.99E-59	1.93E-56
CMTM5	-1.94	2.23	1.39E-51	1.80E-49
DHRS7C	-1.94	2.67	3.37E-25	6.41E-24
CD177	-1.95	-2.44	4.46E-26	9.10E-25
HOPX	-1.96	3.26	1.07E-37	5.10E-36
FKBP5	-1.96	7.30	2.13E-53	3.13E-51
MCEMP1	-1.97	-2.19	1.12E-37	5.31E-36
C1orf105	-2.03	1.41	1.67E-49	1.87E-47
SGPP2	-2.10	2.38	1.22E-51	1.60E-49
IL1R2	-2.11	0.44	9.45E-43	6.57E-41
RP11-766F14.2	-2.14	1.25	4.00E-22	6.05E-21
CYP4Z1	-2.15	-0.89	3.42E-56	6.45E-54
SLCO4A1	-2.21	3.22	5.29E-62	1.36E-59
SCGN	-2.24	0.47	5.60E-40	3.19E-38
AQP4	-2.25	2.05	1.14E-35	4.66E-34
CYP4B1	-2.28	4.28	1.65E-50	2.01E-48
CD163	-2.29	6.35	2.08E-75	1.09E-72
FCN3	-2.34	3.40	2.12E-76	1.19E-73
GNMT	-2.35	-0.24	1.14E-67	3.90E-65
AOX1	-2.36	2.61	6.82E-62	1.70E-59
NPTX2	-2.36	1.95	1.74E-70	7.10E-68
SAA1	-2.41	-0.37	1.93E-17	1.98E-16
PI15	-2.51	-0.51	2.33E-39	1.26E-37
PLA2G2A	-2.54	4.20	2.39E-47	2.25E-45
ALOX15B	-2.56	-1.04	1.01E-37	4.88E-36
LCN10	-2.71	3.57	3.03E-77	2.03E-74
LCN6	-2.76	2.50	8.76E-85	1.22E-81
TUBA3D	-2.80	4.57	1.29E-76	7.43E-74
TUBA3E	-2.82	3.50	1.34E-80	1.32E-77
MYH6	-2.93	9.32	1.43E-84	1.85E-81
RP11-216L13.16	-3.02	-1.36	2.37E-43	1.68E-41
RNASE2	-3.06	-1.19	2.43E-79	2.04E-76
SERPINA3	-3.29	4.66	8.84E-112	4.94E-108
IL1RL1	-3.44	2.90	1.60E-78	1.27E-75

Table S2. Differentially expressed iron-linked genes in DCM at single cell level, obtained from the pseudobulk analysis of scRNA-seq data. pValue was adjusted using Benjamini-Hochberg method ((|logFC|) > 0.5 and FDR adjusted pValue (adj pValue) < 0.05).

	Gene name	logFC	pValue	adj pValue
CARDIOMYOCITES	HBA1	2.54	7.11E-07	2.29E-05
	P3H2	2.04	1.11E-10	2.02E-08
	BMP6	1.70	6.69E-07	2.18E-05
	HBA2	1.68	7.58E-05	9.12E-04
	HPX	1.39	1.64E-04	1.67E-03
	SNCA	1.28	9.98E-07	2.98E-05
	ABCG2	0.94	5.32E-04	4.16E-03
	BCL2	0.90	1.93E-05	3.12E-04
	CYP19A1	0.82	3.63E-03	1.84E-02
	CYP2J2	0.79	1.22E-05	2.19E-04
	AGMO	0.78	9.43E-04	6.50E-03
	CYP2C9	0.77	6.34E-03	2.80E-02
	SCD5	0.73	1.70E-03	1.03E-02
	ARHGAP1	0.70	1.08E-09	1.28E-07
	ALAS2	0.68	8.86E-03	3.58E-02
	CYP26B1	0.63	5.48E-03	2.51E-02
	SLC25A28	0.60	1.62E-07	6.88E-06
	CYP11A1	0.52	1.21E-02	4.54E-02
	SOD1	-0.53	4.13E-06	9.43E-05
	SC5D	-0.53	1.71E-05	2.86E-04
	B2M	-0.56	1.35E-03	8.58E-03
	ATP6V0B	-0.58	2.31E-05	3.58E-04
	NEDD8	-0.59	1.03E-04	1.16E-03
	STEAP4	-0.67	3.63E-03	1.84E-02
	HYAL2	-0.68	8.26E-04	5.86E-03
	SCARA5	-0.71	2.22E-03	1.26E-02
	HMOX2	-0.78	4.20E-13	2.09E-10
	STEAP1	-0.82	4.90E-03	2.30E-02
	SCD	-0.84	4.61E-03	2.21E-02
	CYP4F12	-1.03	5.92E-04	4.54E-03
	STEAP3	-1.14	2.36E-07	9.42E-06
	CYGB	-1.24	3.51E-05	4.94E-04
	C1QA	-1.40	2.04E-06	5.30E-05
	CYP4B1	-1.75	2.57E-06	6.41E-05
FIBROBLASTS	HBA1	3.30	4.70E-09	2.73E-07
	HBA2	2.79	8.90E-09	4.74E-07
	ABCG2	1.52	2.18E-07	6.63E-06
	PAH	1.36	1.53E-04	1.37E-03
	CYP19A1	1.26	6.70E-06	1.09E-04
	PPEF1	1.14	2.17E-05	2.84E-04
	REP15	1.14	6.12E-09	3.47E-07
	NEO1	0.94	6.40E-10	5.14E-08
	BMP6	0.91	8.39E-05	8.46E-04
	HPX	0.66	1.63E-03	9.06E-03
	CCND1	0.65	1.79E-05	2.45E-04
	P3H2	0.63	4.54E-03	2.00E-02
	AGMO	0.55	1.02E-03	6.25E-03
	HEPH	0.53	4.29E-05	4.99E-04
	ADI1	-0.52	7.38E-05	7.63E-04
	SLC48A1	-0.53	6.14E-06	1.01E-04
	PLOD1	-0.54	2.30E-04	1.91E-03
	TFR2	-0.58	3.39E-03	1.59E-02
	CYGB	-0.59	2.85E-05	3.55E-04
	TFRC	-0.61	4.05E-04	3.00E-03
	HMOX1	-0.65	3.11E-03	1.49E-02
	FTH1	-0.66	1.75E-06	3.70E-05
	CYP4A22	-0.68	5.06E-03	2.18E-02
	SCD	-0.69	4.74E-04	3.40E-03
	CYP4X1	-0.71	2.61E-04	2.11E-03
	FLVCR2	-0.72	1.81E-04	1.57E-03
	ALKBH2	-0.78	2.20E-06	4.44E-05
	ISCA2	-0.79	5.65E-07	1.46E-05
	SCD5	-0.85	3.96E-07	1.09E-05
	MAP1LC3A	-0.92	3.32E-08	1.42E-06
	CDO1	-0.97	4.47E-07	1.21E-05
	CYP26B1	-1.03	2.08E-04	1.75E-03
	SCARA5	-1.11	3.04E-09	1.88E-07
	CYP4A11	-1.23	2.49E-04	2.02E-03
	CYP4Z1	-1.34	2.13E-06	4.34E-05
	AOX1	-1.36	8.69E-07	2.08E-05
	C1QA	-1.50	4.37E-08	1.76E-06
	CYP4B1	-2.12	2.24E-10	2.08E-08

MYELOID CELLS	HBA1	3.75	2.32E-08	4.16E-06
	HBA2	2.66	1.17E-07	1.34E-05
	CH25H	1.83	8.73E-07	5.76E-05
	ATP6V0D2	1.38	1.36E-05	4.38E-04
	BMP6	0.82	8.67E-04	8.58E-03
	P3H2	0.79	7.62E-06	2.92E-04
	TF	0.78	6.89E-04	7.33E-03
	CYP2J2	0.60	2.78E-03	1.97E-02
	ABCG2	0.60	4.19E-04	5.09E-03
	CYP27A1	0.57	1.79E-03	1.42E-02
	SLC39A8	-0.52	6.48E-03	3.58E-02
	ISCA2	-0.52	9.63E-04	9.25E-03
	TFR2	-0.65	2.10E-03	1.60E-02
	ALOX5	-0.66	5.76E-10	2.07E-07
	STEAP3	-0.66	4.49E-04	5.38E-03
	SCARA5	-0.68	7.60E-04	7.84E-03
	CYGB	-0.70	1.55E-03	1.29E-02
	ALKBH2	-0.74	4.35E-04	5.25E-03
	CYP1B1	-0.79	1.27E-04	2.20E-03
	SLC11A1	-0.94	4.69E-05	1.09E-03
	CYP4B1	-0.96	3.28E-04	4.25E-03
	CCNB1	-1.48	1.81E-06	1.03E-04
ENDOCARDIAL CELLS	HBA1	4.00	1.99E-12	1.93E-10
	HBA2	3.03	5.22E-08	1.75E-06
	BMP6	2.41	2.42E-16	5.47E-14
	P3H2	2.34	7.11E-13	7.58E-11
	CP	1.49	1.08E-05	1.85E-04
	CYP2D6	1.42	1.81E-04	2.03E-03
	ABCG2	1.26	1.48E-04	1.72E-03
	P3H3	1.20	8.60E-06	1.53E-04
	HAAO	1.16	9.01E-05	1.14E-03
	BCL2	1.00	2.24E-04	2.39E-03
	TF	0.77	2.20E-03	1.46E-02
	CYP27B1	0.76	7.56E-03	3.78E-02
	TET1	0.65	4.04E-04	3.88E-03
	TTC7A	0.59	1.24E-04	1.50E-03
	MYC	0.54	5.63E-03	3.06E-02
	CYBRD1	0.52	5.70E-04	5.05E-03
	MCOLN1	-0.55	1.32E-03	9.89E-03
	SLC48A1	-0.57	4.88E-04	4.49E-03
	KDM7A	-0.62	2.15E-05	3.38E-04
	DNAJC24	-0.65	1.64E-07	4.75E-06
	STEAP4	-0.71	3.40E-03	2.06E-02
	ISCA2	-0.77	7.09E-04	6.03E-03
	NEDD8	-0.81	4.52E-04	4.22E-03
	ATP6V1F	-0.90	1.84E-04	2.05E-03
	C1QA	-1.24	8.08E-05	1.05E-03
	ALOX5	-1.34	5.33E-11	3.79E-09
	CYP4F12	-1.37	7.79E-09	3.31E-07
	HYAL2	-1.45	4.71E-13	5.15E-11
	CYP4B1	-1.71	5.61E-06	1.05E-04
	CYP1B1	-2.38	5.75E-18	1.79E-15
	PPEF1	-2.99	9.60E-17	2.29E-14
ENDOTHELIAL CELLS	HBA1	4.39	4.60E-12	2.31E-09
	HBA2	3.04	3.93E-08	3.73E-06
	BCL2	1.68	2.58E-13	2.63E-10
	P3H3	0.88	7.30E-05	1.21E-03
	CYP46A1	0.60	1.98E-03	1.38E-02
	SLC25A28	0.52	7.98E-06	2.34E-04
	CYGB	-0.51	9.34E-04	8.00E-03
	NOX5	-0.52	6.30E-03	3.20E-02
	ATP6V1F	-0.57	1.68E-04	2.25E-03
	SCD	-0.63	2.04E-03	1.41E-02
	ADI1	-0.70	4.61E-05	8.63E-04
	SCARA5	-0.74	2.39E-04	2.94E-03
	CYP4A11	-1.02	1.78E-04	2.34E-03
	STEAP4	-1.05	1.29E-07	1.00E-05
	C1QA	-1.42	3.83E-08	3.65E-06
	CYP4X1	-1.70	2.98E-10	6.34E-08
	CYP4B1	-2.01	6.18E-12	3.03E-09
	CYP4Z1	-2.24	1.99E-12	1.16E-09

Table S3. GSM number and covariates of selected samples for the bulk RNA-seq dataset.

ID	Study	Diagnosis
GSM3219558	GSE116250	NF
GSM3219559	GSE116250	NF
GSM3219560	GSE116250	NF
GSM3219561	GSE116250	NF
GSM3219562	GSE116250	NF
GSM3219563	GSE116250	NF
GSM3219564	GSE116250	NF
GSM3219565	GSE116250	NF
GSM3219566	GSE116250	NF
GSM3219567	GSE116250	NF
GSM3219568	GSE116250	NF
GSM3219569	GSE116250	NF
GSM3219570	GSE116250	NF
GSM3219571	GSE116250	NF
GSM3219572	GSE116250	DCM
GSM3219573	GSE116250	DCM
GSM3219574	GSE116250	DCM
GSM3219575	GSE116250	DCM
GSM3219576	GSE116250	DCM
GSM3219577	GSE116250	DCM
GSM3219578	GSE116250	DCM
GSM3219579	GSE116250	DCM
GSM3219580	GSE116250	DCM
GSM3219581	GSE116250	DCM
GSM3219582	GSE116250	DCM
GSM3219583	GSE116250	DCM
GSM3219584	GSE116250	DCM
GSM3219585	GSE116250	DCM
GSM3219586	GSE116250	DCM
GSM3219587	GSE116250	DCM
GSM3219588	GSE116250	DCM
GSM3219589	GSE116250	DCM
GSM3219590	GSE116250	DCM
GSM3219591	GSE116250	DCM
GSM3219592	GSE116250	DCM
GSM3219593	GSE116250	DCM
GSM3219594	GSE116250	DCM
GSM3219595	GSE116250	DCM
GSM3219596	GSE116250	DCM
GSM3219597	GSE116250	DCM
GSM3219598	GSE116250	DCM
GSM3219599	GSE116250	DCM
GSM3219600	GSE116250	DCM
GSM3219601	GSE116250	DCM
GSM3219602	GSE116250	DCM
GSM3219603	GSE116250	DCM
GSM3219604	GSE116250	DCM
GSM3219605	GSE116250	DCM
GSM3219606	GSE116250	DCM
GSM3219607	GSE116250	DCM
GSM3219608	GSE116250	DCM
GSM1126612	GSE46224	NF
GSM1126613	GSE46224	NF
GSM1126614	GSE46224	NF
GSM1126615	GSE46224	NF
GSM1126616	GSE46224	NF
GSM1126617	GSE46224	NF
GSM1126618	GSE46224	NF
GSM1126619	GSE46224	NF
GSM3417081	GSE120852	NF
GSM3417082	GSE120852	NF
GSM3417083	GSE120852	NF
GSM3417084	GSE120852	NF
GSM3417085	GSE120852	NF
GSM4215858	GSE141910	NF
GSM4215859	GSE141910	NF
GSM4215860	GSE141910	NF
GSM4215861	GSE141910	NF
GSM4215862	GSE141910	NF
GSM4215863	GSE141910	NF
GSM4215864	GSE141910	NF
GSM4215865	GSE141910	NF
GSM4215866	GSE141910	NF
GSM4215867	GSE141910	NF

GSM4215868	GSE141910	NF
GSM4215869	GSE141910	NF
GSM4215870	GSE141910	NF
GSM4215871	GSE141910	NF
GSM4215872	GSE141910	NF
GSM4215873	GSE141910	NF
GSM4215874	GSE141910	NF
GSM4215875	GSE141910	NF
GSM4215876	GSE141910	NF
GSM4215877	GSE141910	NF
GSM4215881	GSE141910	DCM
GSM4215882	GSE141910	DCM
GSM4215883	GSE141910	DCM
GSM4215884	GSE141910	DCM
GSM4215885	GSE141910	DCM
GSM4215886	GSE141910	DCM
GSM4215888	GSE141910	DCM
GSM4215889	GSE141910	DCM
GSM4215890	GSE141910	DCM
GSM4215891	GSE141910	DCM
GSM4215892	GSE141910	DCM
GSM4215893	GSE141910	DCM
GSM4215894	GSE141910	DCM
GSM4215895	GSE141910	DCM
GSM4215896	GSE141910	DCM
GSM4215897	GSE141910	DCM
GSM4215898	GSE141910	DCM
GSM4215900	GSE141910	DCM
GSM4215901	GSE141910	DCM
GSM4215902	GSE141910	DCM
GSM4215903	GSE141910	DCM
GSM4215904	GSE141910	DCM
GSM4215905	GSE141910	DCM
GSM4215906	GSE141910	DCM
GSM4215907	GSE141910	DCM
GSM4215908	GSE141910	DCM
GSM4215909	GSE141910	DCM
GSM4215910	GSE141910	DCM
GSM4215911	GSE141910	DCM
GSM4215912	GSE141910	DCM
GSM4215913	GSE141910	DCM
GSM4215914	GSE141910	DCM
GSM4215915	GSE141910	DCM
GSM4215916	GSE141910	DCM
GSM4215917	GSE141910	DCM
GSM4215918	GSE141910	DCM
GSM4215919	GSE141910	DCM
GSM4215920	GSE141910	DCM
GSM4215921	GSE141910	DCM
GSM4215922	GSE141910	DCM
GSM4215923	GSE141910	DCM
GSM4215924	GSE141910	DCM
GSM4215925	GSE141910	DCM
GSM4215926	GSE141910	DCM
GSM4215927	GSE141910	DCM
GSM4215928	GSE141910	DCM
GSM4215929	GSE141910	DCM
GSM4215930	GSE141910	DCM
GSM4215931	GSE141910	DCM
GSM4215933	GSE141910	DCM
GSM4215934	GSE141910	DCM
GSM4215935	GSE141910	DCM
GSM4215936	GSE141910	DCM
GSM4215937	GSE141910	DCM
GSM4215938	GSE141910	DCM
GSM4215939	GSE141910	DCM
GSM4215940	GSE141910	DCM
GSM4215942	GSE141910	DCM
GSM4215943	GSE141910	DCM
GSM4215944	GSE141910	DCM
GSM4215945	GSE141910	DCM
GSM4215946	GSE141910	DCM
GSM4215947	GSE141910	DCM
GSM4215948	GSE141910	DCM
GSM4215949	GSE141910	DCM
GSM4215952	GSE141910	DCM
GSM4215953	GSE141910	DCM
GSM4215955	GSE141910	DCM

GSM4215956	GSE141910	DCM
GSM4215957	GSE141910	DCM
GSM4215959	GSE141910	DCM
GSM4215961	GSE141910	DCM
GSM4215962	GSE141910	DCM
GSM4215963	GSE141910	DCM
GSM4215964	GSE141910	DCM
GSM4215965	GSE141910	DCM
GSM4215966	GSE141910	DCM
GSM4215967	GSE141910	DCM
GSM4215968	GSE141910	DCM
GSM4215969	GSE141910	DCM
GSM4215970	GSE141910	DCM
GSM4215971	GSE141910	DCM
GSM4215972	GSE141910	DCM
GSM4215973	GSE141910	DCM
GSM4215974	GSE141910	DCM
GSM4215975	GSE141910	DCM
GSM4215977	GSE141910	DCM
GSM4215978	GSE141910	DCM
GSM4215979	GSE141910	DCM
GSM4215980	GSE141910	DCM
GSM4215981	GSE141910	DCM
GSM4215982	GSE141910	DCM
GSM4215983	GSE141910	DCM
GSM4215984	GSE141910	DCM
GSM4215986	GSE141910	NF
GSM4215987	GSE141910	DCM
GSM4215988	GSE141910	DCM
GSM4215989	GSE141910	NF
GSM4215990	GSE141910	DCM
GSM4215991	GSE141910	NF
GSM4215992	GSE141910	NF
GSM4215993	GSE141910	DCM
GSM4215994	GSE141910	NF
GSM4215995	GSE141910	DCM
GSM4215996	GSE141910	DCM
GSM4215997	GSE141910	DCM
GSM4215998	GSE141910	NF
GSM4215999	GSE141910	NF
GSM4216000	GSE141910	DCM
GSM4216001	GSE141910	NF
GSM4216002	GSE141910	DCM
GSM4216003	GSE141910	DCM
GSM4216004	GSE141910	NF
GSM4216005	GSE141910	NF
GSM4216006	GSE141910	DCM
GSM4216007	GSE141910	NF
GSM4216008	GSE141910	NF
GSM4216009	GSE141910	NF
GSM4216010	GSE141910	NF
GSM4216011	GSE141910	DCM
GSM4216012	GSE141910	NF
GSM4216013	GSE141910	DCM
GSM4216014	GSE141910	NF
GSM4216015	GSE141910	NF
GSM4216016	GSE141910	NF
GSM4216017	GSE141910	NF
GSM4216018	GSE141910	NF
GSM4216019	GSE141910	DCM
GSM4216020	GSE141910	DCM
GSM4216021	GSE141910	NF
GSM4216022	GSE141910	NF
GSM4216024	GSE141910	NF
GSM4216025	GSE141910	NF
GSM4216026	GSE141910	DCM
GSM4216027	GSE141910	NF
GSM4216028	GSE141910	NF
GSM4216029	GSE141910	NF
GSM4216030	GSE141910	NF
GSM4216031	GSE141910	NF
GSM4216032	GSE141910	NF
GSM4216033	GSE141910	DCM
GSM4216034	GSE141910	NF
GSM4216035	GSE141910	NF
GSM4216036	GSE141910	NF
GSM4216037	GSE141910	NF
GSM4216039	GSE141910	NF

GSM4216040	GSE141910	NF
GSM4216041	GSE141910	DCM
GSM4216042	GSE141910	NF
GSM4216043	GSE141910	NF
GSM4216044	GSE141910	NF
GSM4216045	GSE141910	NF
GSM4216047	GSE141910	NF
GSM4216048	GSE141910	NF
GSM4216049	GSE141910	NF
GSM4216050	GSE141910	NF
GSM4216052	GSE141910	DCM
GSM4216053	GSE141910	NF
GSM4216054	GSE141910	NF
GSM4216055	GSE141910	DCM
GSM4216056	GSE141910	NF
GSM4216057	GSE141910	DCM
GSM4216058	GSE141910	DCM
GSM4216059	GSE141910	DCM
GSM4216060	GSE141910	DCM
GSM4216061	GSE141910	NF
GSM4216062	GSE141910	DCM
GSM4216063	GSE141910	NF
GSM4216065	GSE141910	DCM
GSM4216066	GSE141910	DCM
GSM4216067	GSE141910	DCM
GSM4216069	GSE141910	NF
GSM4216070	GSE141910	NF
GSM4216071	GSE141910	DCM
GSM4216072	GSE141910	DCM
GSM4216073	GSE141910	DCM
GSM4216074	GSE141910	DCM
GSM4216075	GSE141910	NF
GSM4216076	GSE141910	DCM
GSM4216077	GSE141910	NF
GSM4216080	GSE141910	DCM
GSM4216081	GSE141910	NF
GSM4216082	GSE141910	NF
GSM4216083	GSE141910	NF
GSM4216084	GSE141910	NF
GSM4216085	GSE141910	NF
GSM4216086	GSE141910	NF
GSM4216087	GSE141910	NF
GSM4216089	GSE141910	NF
GSM4216090	GSE141910	NF
GSM4216091	GSE141910	DCM
GSM4216092	GSE141910	DCM
GSM4216093	GSE141910	NF
GSM4216094	GSE141910	NF
GSM4216095	GSE141910	NF
GSM4216096	GSE141910	DCM
GSM4216097	GSE141910	NF
GSM4216098	GSE141910	NF
GSM4216099	GSE141910	NF
GSM4216100	GSE141910	NF
GSM4216101	GSE141910	NF
GSM4216102	GSE141910	NF
GSM4216103	GSE141910	DCM
GSM4216104	GSE141910	NF
GSM4216105	GSE141910	NF
GSM4216106	GSE141910	NF
GSM4216108	GSE141910	NF
GSM4216109	GSE141910	NF
GSM4216110	GSE141910	NF
GSM4216111	GSE141910	NF
GSM4216112	GSE141910	NF
GSM4216113	GSE141910	NF
GSM4216114	GSE141910	NF
GSM4216115	GSE141910	NF
GSM4216116	GSE141910	NF
GSM4216117	GSE141910	DCM
GSM4216118	GSE141910	NF
GSM4216119	GSE141910	NF
GSM4216120	GSE141910	NF
GSM4216121	GSE141910	NF
GSM4216122	GSE141910	NF
GSM4216123	GSE141910	NF
GSM4216124	GSE141910	NF
GSM4216127	GSE141910	DCM

GSM4216128	GSE141910	DCM
GSM4216129	GSE141910	NF
GSM4216130	GSE141910	DCM
GSM4216131	GSE141910	NF
GSM4216132	GSE141910	NF
GSM4216134	GSE141910	NF
GSM4216135	GSE141910	NF
GSM4216136	GSE141910	NF
GSM4216138	GSE141910	NF
GSM4216139	GSE141910	NF
GSM4216140	GSE141910	DCM
GSM4216141	GSE141910	NF
GSM4216142	GSE141910	NF
GSM4216143	GSE141910	DCM
GSM4216144	GSE141910	NF
GSM4216146	GSE141910	NF
GSM4216147	GSE141910	NF
GSM4216148	GSE141910	NF
GSM4216149	GSE141910	NF
GSM4216150	GSE141910	NF
GSM4216151	GSE141910	NF
GSM4216152	GSE141910	NF
GSM4216153	GSE141910	NF
GSM4216154	GSE141910	NF
GSM4216155	GSE141910	DCM
GSM4216156	GSE141910	NF
GSM4216158	GSE141910	DCM
GSM4216160	GSE141910	NF
GSM4216161	GSE141910	NF
GSM4216162	GSE141910	NF
GSM4216163	GSE141910	NF
GSM4216164	GSE141910	NF
GSM4216165	GSE141910	DCM
GSM4216166	GSE141910	DCM
GSM4216167	GSE141910	DCM
GSM4216168	GSE141910	DCM
GSM4216169	GSE141910	NF
GSM4216170	GSE141910	NF
GSM4216171	GSE141910	NF
GSM4216172	GSE141910	NF
GSM4216173	GSE141910	NF
GSM4216174	GSE141910	NF
GSM4216175	GSE141910	NF
GSM4216176	GSE141910	NF
GSM4216177	GSE141910	NF
GSM4216178	GSE141910	NF
GSM4216179	GSE141910	NF
GSM4216180	GSE141910	DCM
GSM4216181	GSE141910	DCM
GSM4216182	GSE141910	DCM
GSM4216183	GSE141910	DCM
GSM4216184	GSE141910	NF
GSM4216185	GSE141910	NF
GSM4216186	GSE141910	NF
GSM4216187	GSE141910	NF
GSM4216188	GSE141910	NF
GSM4216189	GSE141910	DCM
GSM4216190	GSE141910	NF
GSM4216191	GSE141910	DCM
GSM4216192	GSE141910	NF
GSM4216193	GSE141910	DCM
GSM4216194	GSE141910	NF
GSM4216195	GSE141910	NF
GSM4216196	GSE141910	DCM
GSM4216197	GSE141910	DCM
GSM4216198	GSE141910	NF
GSM4216200	GSE141910	NF
GSM4216201	GSE141910	DCM
GSM4216202	GSE141910	DCM
GSM4216203	GSE141910	DCM
GSM4216204	GSE141910	DCM
GSM4216205	GSE141910	NF
GSM4216206	GSE141910	DCM
GSM4216207	GSE141910	DCM
GSM4216208	GSE141910	NF
GSM4216209	GSE141910	DCM
GSM4216210	GSE141910	DCM
GSM4216211	GSE141910	NF

GSM4216212	GSE141910	NF
GSM4216213	GSE141910	NF
GSM4216214	GSE141910	NF
GSM4216215	GSE141910	DCM
GSM4216216	GSE141910	NF
GSM4216217	GSE141910	DCM
GSM4216221	GSE141910	DCM
GSM4216224	GSE141910	NF
GSM4216226	GSE141910	DCM
GSM4216228	GSE141910	DCM
GSM3605111	GSE126569	NF
GSM3605114	GSE126569	NF
GSM3605116	GSE126569	NF
GSM3605120	GSE126569	NF
GSM3605122	GSE126569	NF
GSM3605123	GSE126569	NF
GSM3605126	GSE126569	NF
GSM3605127	GSE126569	NF
GSM3605128	GSE126569	NF
GSM3605129	GSE126569	DCM
GSM3605133	GSE126569	DCM
GSM3605141	GSE126569	DCM
GSM3605142	GSE126569	DCM
GSM3605143	GSE126569	DCM
GSM3605112	GSE126569	NF
GSM3605113	GSE126569	NF
GSM3605115	GSE126569	NF
GSM3605117	GSE126569	NF
GSM3605118	GSE126569	NF
GSM3605119	GSE126569	NF
GSM3605121	GSE126569	NF
GSM3605124	GSE126569	NF
GSM3605125	GSE126569	NF
GSM3605130	GSE126569	DCM
GSM3605131	GSE126569	DCM
GSM3605132	GSE126569	DCM
GSM3605134	GSE126569	DCM
GSM3605135	GSE126569	DCM
GSM3605136	GSE126569	DCM
GSM3605137	GSE126569	DCM
GSM3605138	GSE126569	DCM
GSM3605139	GSE126569	DCM
GSM3605140	GSE126569	DCM
GSM2891378	GSE108157	NF
GSM2891379	GSE108157	NF
GSM2891381	GSE108157	NF
GSM2891385	GSE108157	NF
GSM3518005	GSE123976	NF
GSM3518006	GSE123976	NF
GSM3518007	GSE123976	NF

Table S4. Gene related to the iron metabolism collect from the molecular signatures database (MSigDB).

**Iron-linked genes**							
ABCB6	ATP6V1A	CYBRD1	CYP2U1	ERFE	HYAL2	P4HA3	SMAD4
ABCB7	ATP6V1B1	CYGB	CYP2W1	ETHE1	IFNG	P4HTM	SNCA
ABCE1	ATP6V1B2	CYP11A1	CYP39A1	FA2H	IREB2	PAH	SOD1
ABCG2	ATP6V1C1	CYP11B1	CYP3A4	FAXDC2	ISCA2	PDX1	SRI
ACO1	ATP6V1C2	CYP11B2	CYP3A43	FBXL5	ISCU	PGRMC2	STEAP1
ACO2	ATP6V1D	CYP17A1	CYP3A5	FDX1	JMJD6	PHF2	STEAP2
ACP5	ATP6V1E1	CYP19A1	CYP3A7	FECH	KDM3A	PHF8	STEAP3
ADI1	ATP6V1E2	CYP1A1	CYP46A1	FLVCR1	KDM7A	PHYH	STEAP4
AGMO	ATP6V1F	CYP1A2	CYP4A11	FLVCR2	LCN2	PICALM	TBXAS1
ALAD	ATP6V1G1	CYP1B1	CYP4A22	FTH1	LMTK2	PLOD1	TCIRG1
ALAS2	ATP6V1G2	CYP20A1	CYP4B1	FTH1P19	LTF	PLOD2	TET1
ALKBH1	ATP6V1G3	CYP21A2	CYP4F11	FTHL17	MAP1LC3A	PLOD3	TET2
ALKBH2	ATP6V1H	CYP24A1	CYP4F12	FTL	MCOLN1	PPEF1	TF
ALKBH3	ATP7A	CYP26A1	CYP4F2	FTMT	MELTF	PPEF2	TFAP2A
ALKBH8	B2M	CYP26B1	CYP4F22	FTO	MIOX	PTGIS	TFF1
ALOX12	BBOX1	CYP26C1	CYP4F3	FXN	MIR210	RAB11B	TFR2
ALOX12B	BCL2	CYP27A1	CYP4F8	G6PD	MMGT1	REP15	TFRC
ALOX15	BDH2	CYP27B1	CYP4V2	GDF2	MSMO1	RIOX1	TH
ALOX15B	BECN1	CYP27C1	CYP4X1	GLRX3	MYC	SC5D	TMEM199
ALOX5	BMP6	CYP2A13	CYP4Z1	HAAO	NCOA4	SCARA5	TMLHE
ALOXE3	BOLA2	CYP2A6	CYP4Z2P	HAMP	NDFIP1	SCD	TMPRSS6
AOX1	BOLA2B	CYP2A7	CYP51A1	HBA1	NECTIN1	SCD5	TPH1
ARHGAP1	BTBD9	CYP2B6	CYP7A1	HBA2	NEDD8	SKP1	TPH2
ASIC3	C1orf194	CYP2C18	CYP7B1	HBQ1	NEO1	SLC11A1	TTC7A
ATP13A2	C1QA	CYP2C19	CYP8B1	HBZ	NFU1	SLC11A2	TTYH1
ATP6AP1	CALR	CYP2C8	DNAJC24	HEPH	NOX5	SLC22A17	TYW5
ATP6V0A1	CAND1	CYP2C9	DNM2	HEPHL1	NUBP1	SLC25A28	XDH
ATP6V0A2	CCDC115	CYP2D6	DOHH	HFE	OGFOD1	SLC25A37	
ATP6V0A4	CCNB1	CYP2D7	DRD2	HIF1A	OGFOD2	SLC39A14	
ATP6V0B	CCND1	CYP2E1	EGLN1	HIF1AN	OGFOD3	SLC39A8	
ATP6V0C	CDO1	CYP2F1	EGLN2	HJV	P3H1	SLC40A1	
ATP6V0D1	CH25H	CYP2G1P	EGLN3	HMOX1	P3H2	SLC46A1	
ATP6V0D2	CLTC	CYP2J2	EIF2AK1	HMOX2	P3H3	SLC48A1	
ATP6V0E1	CP	CYP2R1	EPAS1	HPX	P4HA1	SLC6A3	
ATP6V0E2	CUL1	CYP2S1	EPB42	HRG	P4HA2	SLC6A9	
